# MiR-134-5p inhibits the malignant phenotypes of osteosarcoma via ITGB1/MMP2/PI3K/Akt pathway

**DOI:** 10.1038/s41420-024-01946-z

**Published:** 2024-04-25

**Authors:** Lei Yan, Ruhao Zhou, Yi Feng, Ruoqi Li, Long Zhang, Yongchun Pan, Xiaochen Qiao, Pengcui Li, Xiaochun Wei, Chaojian Xu, Yuan Li, Xiaochen Niu, Xiaojuan Sun, Zhi Lv, Zhi Tian

**Affiliations:** 1 Second Clinical Medical College, Shanxi Medical University, 382 Wuyi Road, Taiyuan, Shanxi 030001 China; 2 Department of orthopedics, The Second Hospital of Shanxi Medical University, Shanxi Key laboratory of Bone and Soft Tissue injury repair, 382 Wuyi Road, Taiyuan, Shanxi 030001 China; 3 General Surgery Department, Third Hospital of Shanxi Medical University, Shanxi Bethune Hospital, Shanxi Academy of Medical Sciences, Tongji Shanxi Hospital, Taiyuan, 030032 China; 4 School of Medicine, Xiamen University, Xiamen, 361102 China; 5 Academy of Medical Sciences, Tongji Shanxi Hospital, Taiyuan, 030032 China; 6 Department of Orthopedics, JinZhong Hospital Affiliated to Shanxi Medical University, 689 Huitong South Road, Jinzhong, Shanxi 030600 China; 7 Shanxi Bethune Hospital, Shanxi, China; 8 The Fifth Clinical Medical College of Shanxi Medical University, Shanxi, China

**Keywords:** Bone cancer, Non-coding RNAs

## Abstract

Micro RNAs (miRs) have been implicated in various tumorigenic processes. Osteosarcoma (OS) is a primary bone malignancy seen in adolescents. However, the mechanism of miRs in OS has not been fully demonstrated yet. Here, miR-134-5p was found to inhibit OS progression and was also expressed at significantly lower levels in OS tissues and cells relative to normal controls. miR-134-5p was found to reduce vasculogenic mimicry, proliferation, invasion, and migration of OS cells, with miR-134-5p knockdown having the opposite effects. Mechanistically, miR-134-5p inhibited expression of the ITGB1/MMP2/PI3K/Akt axis, thus reducing the malignant features of OS cells. In summary, miR-134-5p reduced OS tumorigenesis by modulation of the ITGB1/MMP2/PI3K/Akt axis, suggesting the potential for using miR-134-5p as a target for treating OS.

## Introduction

Osteosarcoma (OS) is a primary tumor of bone, most frequent in young patients between the ages of 15 and 19 years, and originating from mesenchymal tissue [1–3]. OS is highly heterogeneous and carries a high risk of metastasis and recurrence [4]. The survival rates of patients with OS have not improved significantly over the past three decades [5]. This is due to difficulties in treatment, resulting from the complexity of the OS karyotype and a high degree of chromosomal and genomic instability [6]. Even with advances in the treatment of surgical resection under general anesthesia combined with chemotherapy and radiotherapy, only 65–70% of patients with OS achieve a curative outcome [7, 8]. Besides, little progress has been made in improving survival in OS patients over the past 40 years [9]. Thus, new developments are urgently required for OS treatment.

MicroRNAs (miRs) have been implicated in numerous physiological activities [10]. MiRNAs interact with the 3′ UTRs of mRNAs, inducing cleavage of the target mRNA or suppression of its translation [11, 12]. Abnormal expression of even one miRNA may influence tumor development [13]. Investigation of the specific roles of miRNAs in tumors is thus important [13]. Specific miRNAs have also been found to be useful biomarkers for different cancers [14, 15]. Vasculogenic mimicry (VM) is an alternative form of angiogenesis derived from tumor cells rather than vascular endothelial cells. VM positivity has been shown to be an independent prognostic factor of OS [16]. To date, the knockdown of lncRNA n340532 [17], MIG-7 [18], and VEGF [19] has been shown to inhibit VM in OS. Besides, *Paris polyphylla* extracts [20] and zoledronic acid [21] have been shown to inhibit VM in OS. However, the mechanism underlying OS VM requires further investigation.

A previous study reported the results of a global microarray analysis of miRNA levels in 19 human OS cell lines and four human bone tissue samples, finding that miR-134-5p was one of 154 miRNAs that were differentially expressed in OS [22]. Several studies have investigated the functions of miR-134-5p. In lung adenocarcinoma, miR-134-5p was shown to promote both chemoresistance and metastasis through the targeting of DAB2 [23], while in gastric cancer, miR-134-5p inhibited tumor progression and invasion by targeting YWHAZ [24]. Our team’s previous studies have demonstrated that miR-134-5p can inhibit the proliferation and metastasis of osteosarcoma [25, 26]. VM is an important malignant phenotype in tumor development. However, the relationship between miR-134-5p and VM has not yet been fully demonstrated. Here, it was demonstrated for the first time that miR-134-5p promoted VM, proliferation, invasion, and migration of OS via the ITGB1/MMP2/PI3K/Akt pathway.

## Results

### miR-134-5p inhibits VM, proliferation, migration, and invasion of OS cells

**Fig. 1:**
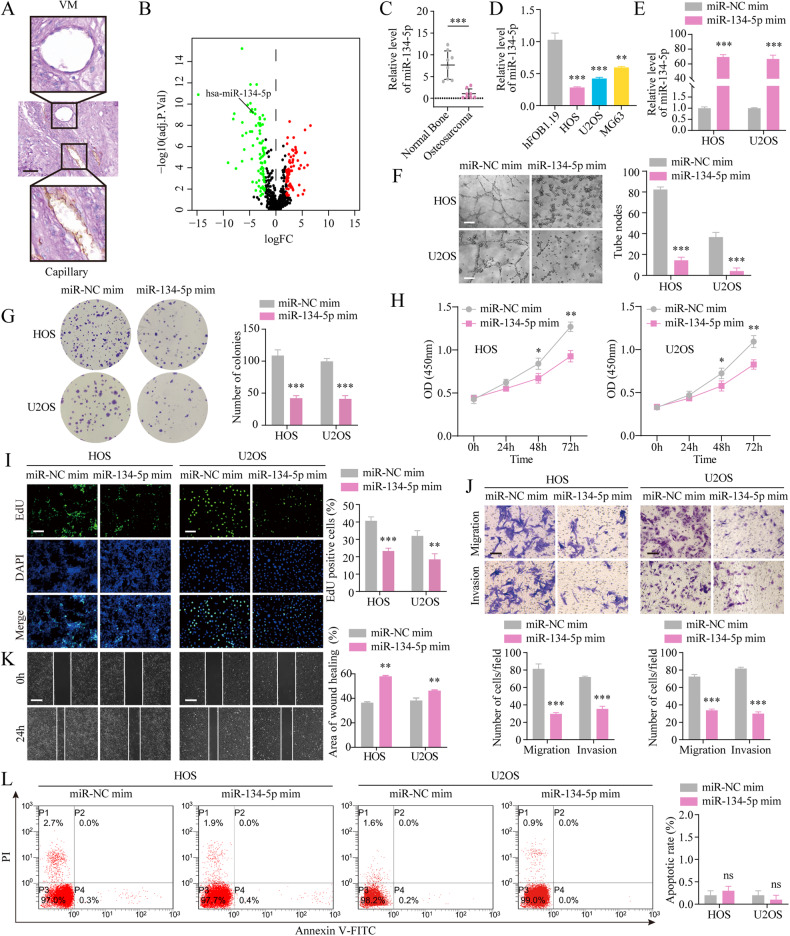
miR-134-5p mimic blocks VM, proliferation, migration, and invasion in OS. **A** CD31/PAS double-staining showing immunohistochemical staining for VM in OS. (Scale bar, 50 μm). **B** Gene expression profiles of GSE28423 are shown as volcano plots. Upregulated DEGs are represented in red and downregulated DEGs are represented in green. **C** Reduced miR-134-5p levels in OS tissue (*n* = 8) vs normal bone (*n* = 8), shown by qRT-PCR. Statistics using independent Student’s *t*-test. **D** Decreased miR-134-5p levels in OS cells versus osteoblasts. **E** Expression of miR-134-5p in transfected cells, shown by qRT-qPCR. **F** Tube formation assays to assess effects of the miR-134-5p mimic on VM. Scale bar, 200 μm. **G**–**I** Colony formation, CCK-8, and EdU assays (scale bar, 400 μm) to evaluate the effects of the miR-134-5p mimic on cell proliferation. **J** Transwell assays to evaluate the effects of the miR-134-5p mimic on cell migration and invasion. Scale bar, 100 μm. **K** Wound-healing assays to evaluate the effects of the miR-134-5p mimic on migration. Scale bar, 100 μm. **L** Flow cytometry to evaluate the effects of the miR-134-5p mimic on apoptosis. The data represent means ± SD. Statistics using one-way ANOVA with Tukey’s test. ns not significant; *p < 0.05; **p < 0.01; ***p < 0.001.

CD31/PAS staining showed the presence of characteristic VM structures in OS tissues, with the establishment of connections between cells forming a vascular lumen containing occasional erythrocytes, and the presence of a purple-colored net-like formation (Fig. 1A). To identify the critical miRNAs involved in OS progression, we examined the miRNA expression profiles in the GSE28423 dataset, which included information from 19 human OS cell lines and four human bone tissues [22]. Using the criteria of *P* value < 0.05 and log2|FC | ≥ 2, miR-134-5p was found to be one of 154 miRNAs identified in the database as differentially expressed (Fig. 1B). Furthermore, lower levels of miR-134-5p were observed in OS compared with normal tissue (Fig. 1C), as well as in OS cells relative to osteoblasts (Fig. 1D). Thus, miR-134-5p was selected for further analyses.

After transfection of cells with lentivirus (Fig. 1E), there were significantly fewer VM structures visible in cells overexpressing miR-134-5p (Fig. 1F), accompanied by a marked reduction in the proliferation of HOS and U2OS cells (Fig. 1G–I). In addition, both invasion and migration were dramatically reduced in OS cells overexpressing miR-134-5p (Fig. 1J, K) However, no significant change in apoptosis levels was observed after overexpression of miR-134-5p (Fig. 1L).

The miR-134-5p knockdown efficiency after transduction of OS cell lines with the miR-134-5p inhibitor or control lentivirus was evaluated using qRT-PCR (Fig. S1A). This showed that miR-134-5p knockdown resulted in markedly increased VM, proliferation, invasion, and migration in the cells, thus supporting (Fig. S1B–G) the anti-cancer function of miR-134-5p in OS.

### Identification of miR-134-5p targets

**Fig. 2:**
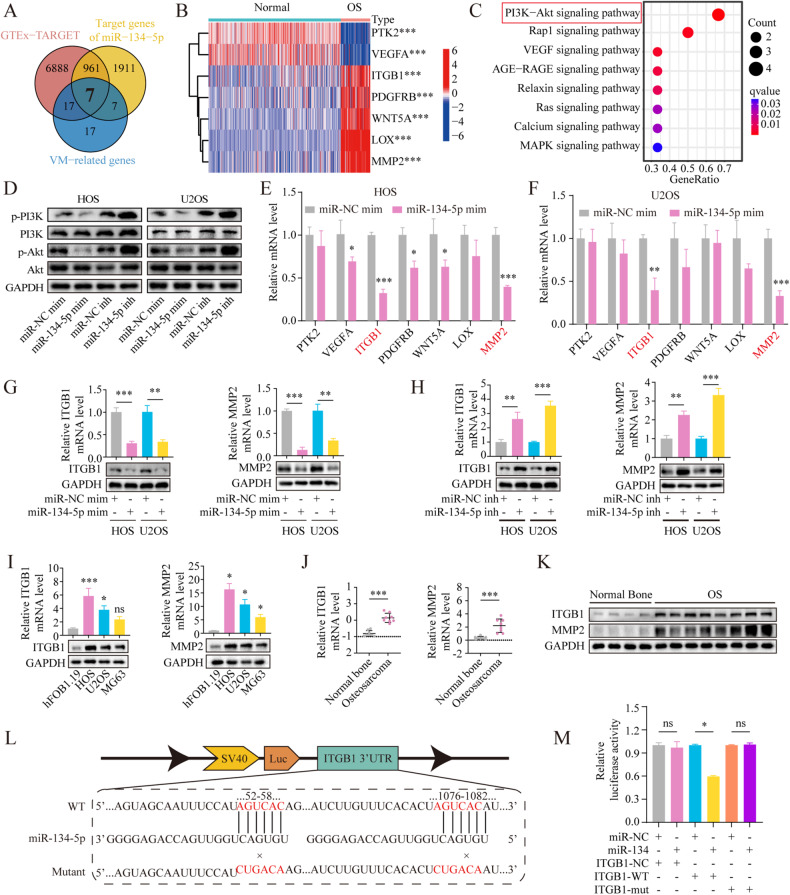
Identification of miR-134-5p target genes in OS. **A** The Venn diagram shows the intersection of three sets: 48 VM-associated genes, 2886 predicted miR-134-5p target genes, and 7873 DEGs. **B** Heatmap showing expression of seven genes between OS and control tissue in the GTEx-TARGET cohort. **C** KEGG enrichment analysis of the seven candidate miR-134-5p target genes. **D** Protein levels of p-PI3K, PI3K, p-Akt, and Akt in OS cells following transfection with the miR-134-5p mimic or miR-134-5p inhibitor or the corresponding negative control. **E**, **F** mRNA expression of seven candidate genes in cells transfected with the miR-134-5p mimic. **G** Protein and mRNA levels of MMP2 and ITGB1 in miR-134-5p mimic-transfected OS cells versus control. **H** Protein and mRNA levels of MMP2 and ITGB1 in miR-134-5p-knockdown OS cells versus control. **I** Protein and mRNA levels of MMP2 and ITGB1 in OS cells versus osteoblasts. Statistics using one-way ANOVA with Tukey’s test. **J**, **K** Protein and mRNA levels of MMP2 and ITGB1 in OS tissues versus normal bone. Statistics using independent Student’s *t*-test. **L** Wild-type and mutant binding sites for ITGB1 in miR-134-5p. **M** Luciferase assay of HOS cells transfected with miR-134-5p mimic (10 nM) or control miR-NC and plasmids containing 3′ UTRs of ITGB1. Statistics using one-way ANOVA with Tukey’s test. The data represent means ± SD. ns not significant; *p < 0.05; **p < 0.01; ***p < 0.001.

After merging three gene sets, including the 7873 DEGs, 48 VM-associated genes [27], and 2886 prediction target genes for miR-134-5p, seven candidate target genes were finally identified (Fig. 2A). The chromosomal localization of the seven genes is shown in Fig. S2A. PPI networks of the seven candidate target genes were constructed (Fig. S2B), and the diagnostic abilities of the genes were examined with receiver operating characteristic (ROC) curves. All seven genes were found to be potential markers for OS diagnosis (Fig. S2C). The comparative expression of these genes between normal and OS tissue in the GTEx-TARGET dataset is shown in the heatmap (Fig. 2B). KEGG analysis (Fig. 2C and Table S6) of the candidate genes showed significant enrichment in the PI3K-Akt pathway while GO analysis (Fig. S2D and Table S7) demonstrated significant enrichment in processes associated with cell chemotaxis and wound healing. Furthermore, MMP2 and ITGB1 were found to be strongly correlated (Fig. S2E). As shown by western blotting, miR-134-5p effectively blocked the phosphorylation of PI3K and Akt (Fig. 2D). All full and uncropped western blots were displayed in a file named “original western blots”. Evaluation of mRNA levels after miR-134-5p mimic transduction (Fig. 2E, F) indicated that MMP2 and ITGB1 were the most significantly downregulated; these two genes were thus used for further analyses. The results of GSEA pathway analysis show that high MMP2 levels were correlated with focal adhesion and oxidative phosphorylation, while high ITGB1 levels were enriched in pathways involved in focal adhesion and cytoskeletal regulation (Fig. S2F, G).

The influence of miR-134-5p on MMP2 and ITGB1 levels was then examined by cell transduction with an miR-134-5p mimic and subsequent measurement of MMP2 or ITGB1 levels. The qRT-PCR and western blotting analyses indicated that miR-134-5p decreased the levels of both MMP2 and ITGB1 (Fig. 2G, H). Besides, both MMP2 and ITGB1 levels are found to be significantly upregulated in human OS cells (Fig. 2I) and tissues (Fig. 2J, K). Potential binding between miR-134-5p, ITGB1, and MMP2 was then examined. By prediction, MMP2 was found to have no binding target for miR-134-5p, while ITGB1 had a binding target for miR-134-5 (Fig. 2L). Luciferase assays showed a decrease in luciferase activity after cell transfection of ITGB1-WT reporter vectors with the miR-134-5p mimic, while no changes were seen after transfection with the scrambled sequences. However, no luciferase activity was observed with mutated binding sequences (Fig. 2M).

### miR-134-5p was found to regulate MMP2 expression by regulating the expression of ITGB1

**Fig. 3:**
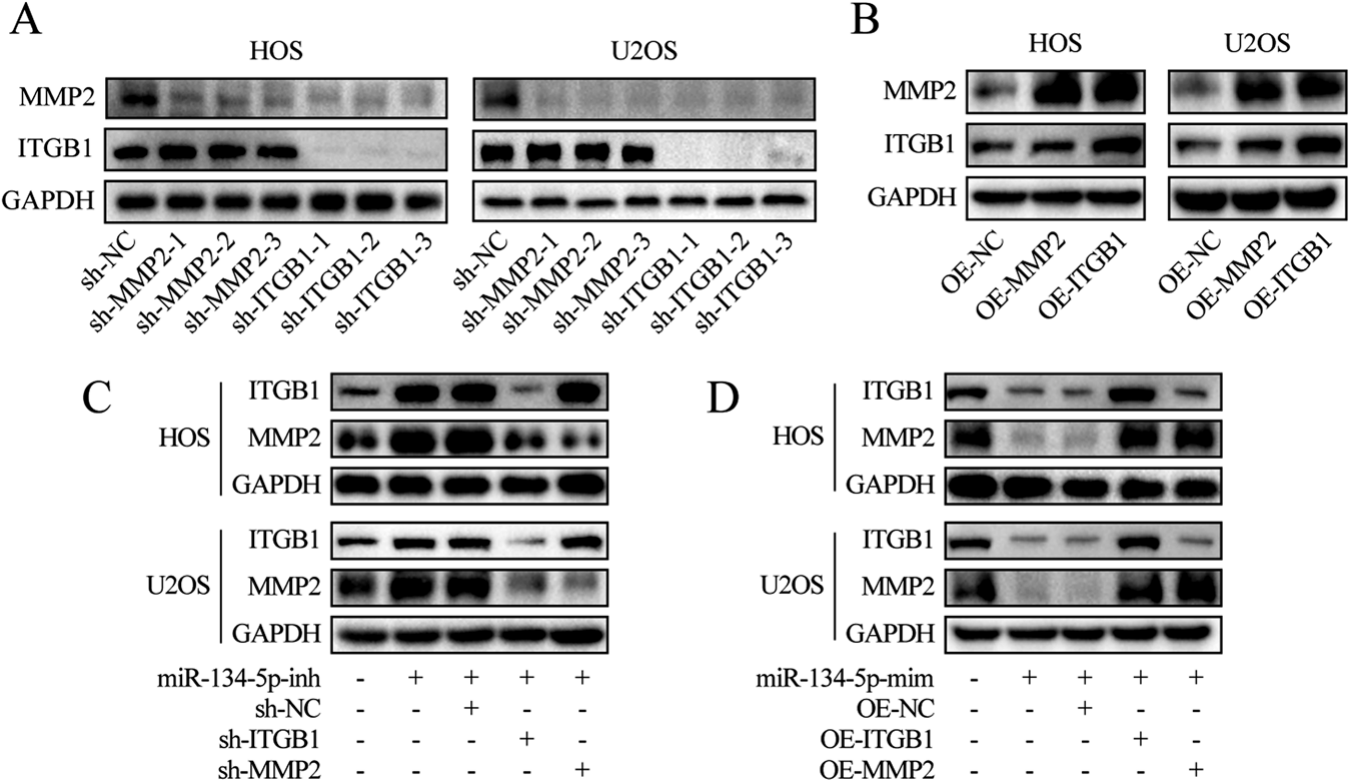
MMP2 changes with ITGB1 in OS cells. **A** Protein levels of MMP2 and ITGB1 in OS cells transfected with knockdown lentivirus versus the corresponding negative control. **B** Protein levels of MMP2 and ITGB1 in OS cells transfected with overexpression lentivirus versus the corresponding negative control. **C**, **D** Protein levels of MMP2 and ITGB1 in different groups of OS cells.

miR-134-5p cannot directly target MMP2 mRNA, but the addition of miR-134-5p mimics results in a decrease in MMP2 expression. We also found a significant positive correlation between MMP2 and ITGB1 by analyzing the correlation between the seven candidate target genes (Fig. S2E). So, we guessed that miR-134-5p may regulate MMP2 expression by regulating the expression of ITGB1. First, three short hairpin RNAs (shRNAs) for MMP2 and ITGB1 were designed. Of these, sh-ITGB1-1 and sh-MMP2-1 were found to be the most efficient and were thus used subsequently for knockdown experiments (Fig. 3A). Surprisingly, decreased MMP2 protein levels were observed in ITGB1 knockdown cells. Furthermore, western blotting showed that the knockdown of ITGB1 rescued the reduced MMP2 protein expression caused by miR-134-5p, although the knockdown of MMP2 was unable to rescue the decrease in ITGB1 caused by miR-134-5p (Fig. 3C). Conversely, overexpression of MMP2 and ITGB1 showed a similar trend (Fig. 3B, D). Together, these findings demonstrate that miR-134-5p reduced MMP2 expression by decreasing the expression of ITGB1.

### miR-134-5p acts through the ITGB1/MMP2/PI3K/Akt axis

**Fig. 4:**
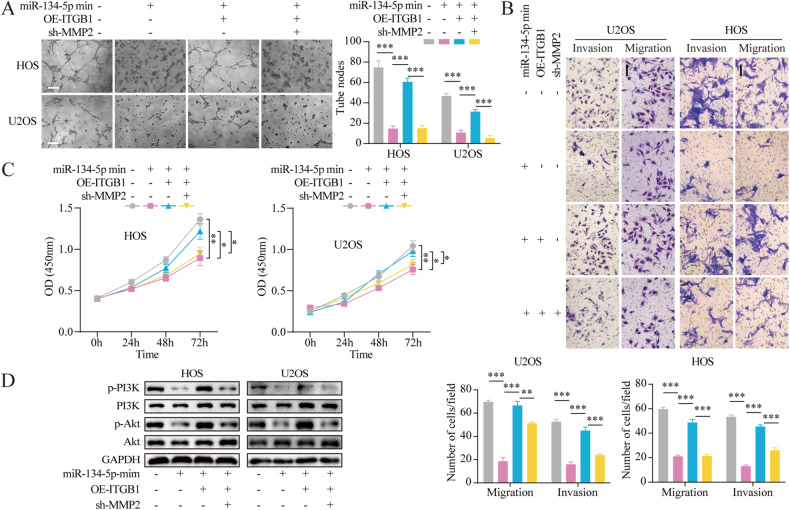
Loss-of-function analyses verifying the involvement of the miR-134-5p/ITGB1/MMP2 axis in OS VM, proliferation, migration, and invasion. **A** Tube formation assays to evaluate the effects of the miR-134-5p/ITGB1/MMP2 axis on VM. Scale bar, 200 μm. **B** Transwell assays to evaluate the effects of the miR-134-5p/ITGB1/MMP2 axis on migration and invasion. Scale bar, 100 μm. **C** CCK-8 assay were to evaluate the effect of miR-134-5p/ITGB1/MMP2 axis on cell proliferation. **D** Protein levels of p-PI3K, PI3K, p-Akt, and Akt, shown by western blotting. Statistics using one-way ANOVA with Tukey’s test. The data represent means ± SD. *p < 0.05; **p < 0.01; ***p < 0.001.

First, cells stably expressing the miR-134-5p mimic were transfected with the overexpression ITGB1 (OE-ITGB1) alone or together with sh-MMP2. The reductions in VM seen in the miR-134-5p mimic-expressing cells were significantly mitigated by OE-ITGB1. However, this effect was counteracted by co-transfection with sh-MMP2 (Fig. 4A). Transwell assay showed that the inhibition of cell invasion and migration induced by miR-134-5p was significantly attenuated by OE-ITGB1. In addition, in cells stably expressing the miR-134-5p mimic, the prevention of cell mobility induced by miR-134-5p recovered after co-transfection with OE-ITGB1 and sh-MMP2 (Fig. 4B). Furthermore, OE-ITGB1 abolished miR-134-5p mimic-induced inhibition of proliferation, while re-transfection with sh-MMP2 reduced these actions (Fig. 4C). Similar effects were seen in the regulatory actions of the miR-134-5p/ITGB1/MMP2 axis on components of the PI3K/Akt pathway (Fig. 4D). The results of functional rescue experiments in miR-134-5p-knockdown cells (Fig. S3) demonstrated that inhibition of miR-134-5p stimulated the PI3K/Akt pathway through activation of the ITGB1/MMP2 axis, thus facilitating the VM, proliferation, migration, and invasion of OS cells.

**Fig. 5:**
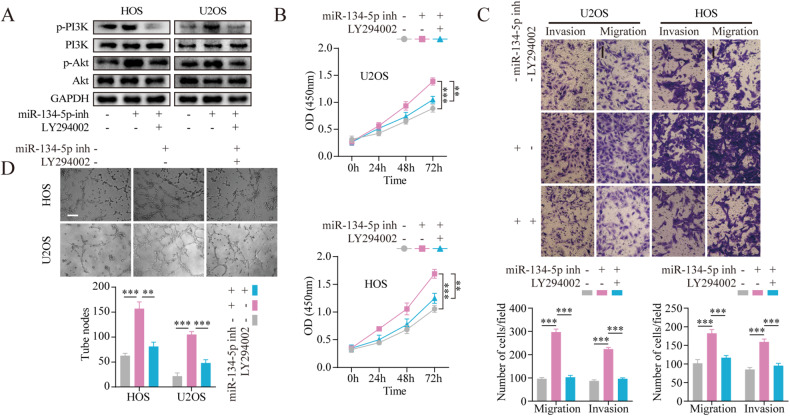
LY294002 rescues miR-134-5p-mediated promotion of OS cell VM, proliferation, migration, and invasion. **A** Protein levels of p-PI3K, PI3K, p-Akt, and Akt in different groups. **B** CCK-8 assay to evaluate the effects of the miR-134-5p/PI3K/Akt axis on cell proliferation. **C** Transwell assays to evaluate the effects of the miR-134-5p/PI3K/Akt axis on migration and invasion. Scale bar, 100 μm. **D** Tube formation assays to evaluate the effects of the miR-134-5p/PI3K/Akt axis on VM. Statistics using one-way ANOVA with Tukey’s test. Scale bar, 200 μm. The data represent means ± SD. **p < 0.01; ***p < 0.001.

To confirm the observed molecular processes, the effects of miR-134-5p knockdown together with inhibition of PI3K (using LY294002 at a concentration of 20 μM) on the malignant phenotype of OS cells were evaluated. Treatment with the miR-134-5p inhibitor was found to raise phosphorylated PI3K and Akt levels in OS cells, while these effects were counteracted by exposure to LY294002 (Fig. 5A). It was also verified that the miR-134-5p inhibitor markedly enhanced the malignant phenotype, which was reversed by treatment with LY294002 (Fig. 5B–D).

**Fig. 6:**
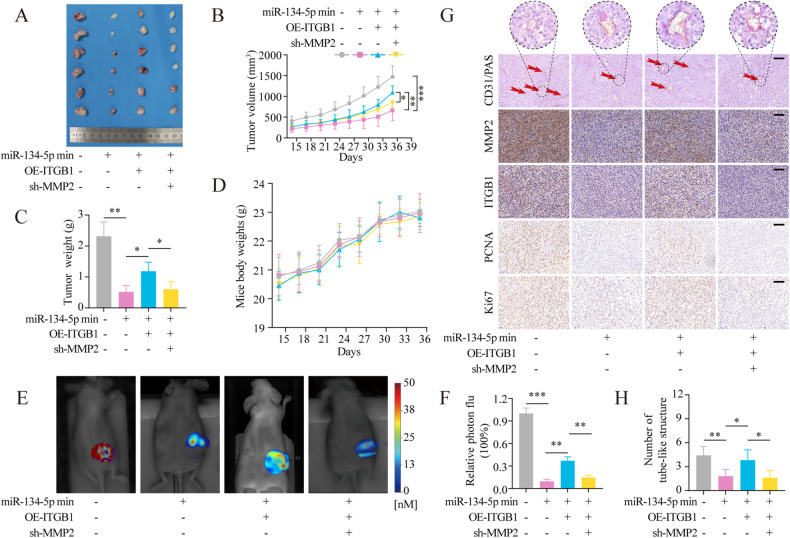
Loss-of-function analyses verifying the involvement of the miR-134-5p/ITGB1/MMP2 axis in mouse xenograft tumor proliferation and VM. **A** Tumor images. **B** Tumor volumes. **C** Tumor weights. **D** Mice body weights. **E**, **F** Bioimaging of tumors using fluorescence molecular tomography and MMPSense 680 to quantify MMP activity in tumors. **G**, **H** Immunohistochemical staining of tumors; the red arrow indicates the VM. Scale bar, 100 μm. Statistics using one-way ANOVA with Tukey’s test. The data represent means ± SD. *p < 0.05; **p < 0.01; ***p < 0.001.

**Fig. 7:**
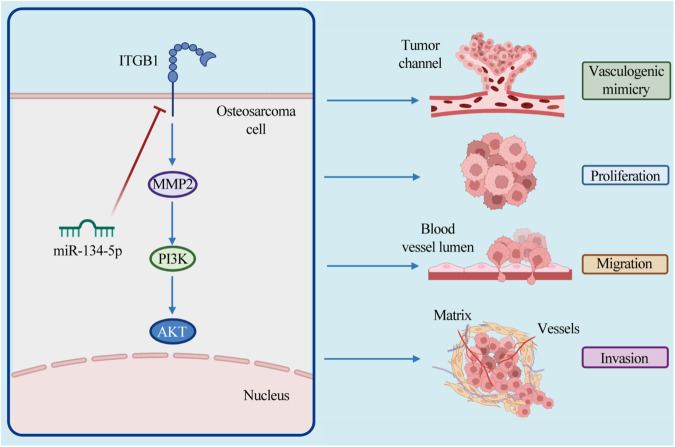
Schematic diagram of the proposed biological function of the miR-134-5p/ITGB1/MMP2/PI3K/Akt axis in OS VM, proliferation, migration, and invasion.

In the in vivo experiments, the miR-134-5p mimic was found to reduce the formation of tumors in mice receiving control cells. Furthermore, ITGB1 overexpression abolished these effects and promoted both growth in OS cells, while this was reversed by MMP2 knockdown (Fig. 6A-C). Mouse body weights were not significantly different between the three groups (Fig. 6D), and no significant toxicity was observed (Fig. S4). The results of in vivo fluorescence in animals followed the same trend as above (Fig. 6E, F). Besides, the results of IHC also showed the same trend in VM, proliferation, ITGB1, and MMP2 (Fig. 6G, H). The schematic shows the role of miR-134-5p in OS by affecting the ITGB1/MMP2/PI3K/Akt axis (Fig. 7).

## Discussion

OS is the most common bone malignancy and is linked to high levels of metastasis and mortality [28]. It has been estimated that about 80% of OS cases have subclinical metastases in the lungs at diagnosis [29]. Management of OS is impeded by an absence of accurate tumor markers. Recent demonstrations of dysregulated noncoding RNAs in OS have attracted much interest. miRNAs have been linked with a variety of functions [30–32] and their abnormal expression is linked to cancer-associated behaviors [33–44]. This highlights the importance of understanding the functions of miRs associated with tumors.

As an important epigenetic factor, miR-134-5p has been shown to inhibit the progression of gastric cancer [24], breast cancer [45], thyroid-like cancer [46], melanoma [47], and esophageal squamous cell carcinoma [48]. However, Zhang et al. reported that miR-134-5p, by targeting DAB2, can promote metastasis and chemoresistance in lung cancer [23]. The different roles played by miR-134-5p in different cancers may be due to the different tumor microenvironments in different cancers. Here, miR-134-5p was shown to contribute to OS tumorigenesis. This was seen in the downregulation of miR-134-5p in OS cells and tissues. Investigations into the function of miR-134-5p showed that it promoted VM, proliferation, migration, and invasion in OS cells. However, interactions between tumor-malignant phenotypes are possible. For example, miR-134-5p can directly inhibit VM by inhibiting ITGB1 and MMP2. Meanwhile, miR-134-5p’s role in indirectly inhibiting VM by inhibiting proliferation may also exist at the same time. Wang et al.‘s study indicates that proliferation can be inhibited by inhibiting tumor VM. So, it is also possible that miR-134-5p indirectly inhibits proliferation by inhibiting VM [49]. At present, we cannot distinguish whether there is a causal relationship between different malignant phenotypes, but our research concludes that miR-134-5p can simultaneously inhibit the proliferation of OS and VM.

MiR acts through its specific targets and target-mediated downstream pathways. Target genes regulated by the same miRNAs may also differ depending on cell and tissue type and different stress or disease environments, possibly due to different gene expression and regulatory profiles [10, 50, 51]. Integrins are cell membrane receptors that mediate mutual recognition and adhesion between cells and cells and between cells and the extracellular matrix, and have a role in linking the external role of cells to their internal structure [52]. ITGB1, as an important member of the integrin family, has previously been linked to VM [53–55], another mode of blood supply to tumors. Besides, miR-134-5p was shown to inhibit osteoclastogenesis by targeting the ITGB1/MAPK pathway [56]. In this study, binding between miR-134-5p and ITGB1 was demonstrated in OS. Overexpression of the ITGB1 was able to counteract the suppression of tumorigenic behaviors by miR-134-5p, whereas silencing of ITGB1 counteracted the effects of the miR-134-5p inhibitor.

Metalloproteinases have been shown to play a crucial role in the formation of VM networks. As a member of the matrix metalloproteinase gene family, MMP2 is a zinc-dependent enzyme and is the most widely recognized regulatory molecule involved in VM formation [57–60]. Microarray gene microarray analyses have shown a significant increase in MMP2 expression in aggressive melanoma cells compared to poorly invasive melanoma cells, which are required for increased MMP2 expression for VM [61, 62]. Our study found that ITGB1 regulates MMP2 expression, which modulates the malignant phenotype of osteosarcoma. Lei Zhang et al. also found that inhibition of ITGB1 suppressed the expression of MMP9 and MMP2 [63]. However, the exact mechanism through which ITGB1 regulates MMP2 expression has not yet been investigated. Zhu et al. reported that ITGB1 can regulate MMP9 expression by regulating the FAK/Src/β-Catenin axis [64]. Thus, the specific mechanism by which ITGB1 regulates MMP2 may have many intermediate variables that need to be explored in future studies.

In addition, we found that the tumorigenic effects mediated by the miR-134-5p/ITGB1/MMP2 axis in OS involved PI3K/Akt signaling. PI3K/Akt signaling contributes to many cellular processes and is often seen to be abnormally activated in cancers [65, 66]. Zhu et al. labeled that MMP2 promotes laryngeal squamous cell carcinoma in a PI3K/Akt/NFκB-dependent manner [67]. Furthermore, activation of the PI3K/ AKT pathway in the tumor microenvironment promotes the formation of VM in melanoma [68] and colorectal cancer [69].

In summary, the above findings together demonstrate that miR-134-5p can inhibit VM and other malignant phenotypes of osteosarcoma by inhibiting the ITGB1/MMP2/PI3K/AKT pathway. This novel axis can aid in appealing treatment modality concerning angiogenesis in osteosarcoma patients.

## Materials and methods

### Clinical tissue specimens

This study was approved by the local ethics committee of the Second Hospital of Shanxi Medical University and was carried out in accordance with the standards set by the Declaration of Helsinki. Eight osteosarcoma samples and eight normal bone tissue samples were obtained from patients. The study protocol was approved by the Ethics Review Committee of the Second Hospital of Shanxi Medical University (approval number DW2022054) in Taiyuan, Shanxi, China. The clinicopathological characteristics are shown in Table S1.

### Cell lines and culture

Human OS cell lines (HOS, U2OS, and MG63) were purchased from the ATCC. Cells were maintained in DMEM containing 10% fetal bovine serum (FBS) and 1% penicillin/ streptomycin (Invitrogen, USA) in a humidified incubator at 37 °C with 5% CO_2_. The osteoblast cell line hFOB1.19 was grown in DMEM/F-12 (Gibco, USA) containing 10% FBS, 2.5 mM l-glutamine (Invitrogen), 0.3 mg/ml geneticin (Gibco), and antibiotics as above.

### Cell transfection

Cells were grown in six-well plates until 40% confluent, after which they were transduced with lentiviral (Genechem) for 24 h and selected with puromycin (3 mg/mL) for 24 h. Transfections with lentivirus were performed with a MOI = 20 in the HOS cell line and a MOI = 10 in the U2OS cell line. The stability of expression was determined.

### RNA isolation and qRT-PCR

RNA (both total and miRNA) was extracted from OS samples, cells, and transfected cells with TRIzol (Thermo Fisher Scientific) and the miRNeasy Mini Kit (Qiagen, MD, USA) according to the provided directions. Gene expression relative to 18S rRNA or U6 was measured by qRT-PCR using an IQ5 Multicolor Real-Time PCR Detection system (Bio-Rad Laboratories, CA, USA). PCR detection of miR-134-5p included reaction at 95 °C (3 min), 95 °C (15 s) for 40 cycles, 56.5 °C (15 s), and 72 °C (30 s). Primer sequences are provided in Table S2. Relative expression was determined by the 2^−ΔΔCT^ method [70].

### Immunohistochemistry

Tissue samples from xenograft tumors were fixed with 4% paraformaldehyde for 14 h, paraffin-embedded, and sectioned. After dewaxing, rehydration, and blocking (5% BSA), the sections were treated with antibodies against MMP2 (1:100) (ab92536, Abcam, UK), ITGB1 (1:50) (ab30394, Abcam), CD31 (1:100) (3528, Cell Signaling Technology, USA), Ki67 (1:1000) (ab15580, Abcam), and PCNA (1:10000) (2586, Cell Signaling Technology) overnight at 4 °C.

### Periodic Acid-Schiff (PAS)/CD31 double-staining

Staining with CD31 was conducted first following the procedure described above. The reactions were visualized with DAB. PAS staining was subsequently performed, in accordance with the provided protocols in a PAS staining kit (G1281, Solarbio, China).

### Measurement of tube formation

Plates (24-well) were coated with Matrigel (354234; Corning). Cells (10^5^/well in medium) were inoculated and grown for 4 h at 37 °C. The numbers of tube nodes were determined using the Angiogenesis Analyzer in ImageJ.

### Cell proliferation assays

CCK-8 assays were performed by seeding and maintaining 6000 cells per well in 96-well plates for 1, 2, or 3 days when the medium was replaced with 100 µL of fresh medium with 10 µL of the CCK-8 reagent (Sigma). After 2 h, absorbances at 450 nm were measured. Cells were fixed and permeabilized after the addition of 10 μM of EdU and incubated for 2 h. The Click-iT reaction was performed following the manufacturer’s manual (Thermo Fisher Scientific). Lastly, nuclei were counterstained with DAPI, and the cells were imaged under a fluorescence microscope (Thermo Fisher Scientific). In colony formation assays, 600 cells/well were inoculated and cultured for 10 days in 6-well plates. After washing with PBS, cells were fixed with 4% paraformaldehyde for 30 min and stained with 0.5% crystal violet for 1 h at room temperature. Colony numbers were assessed using a clone-counter program.

### Wound-healing assay

Cells (10^6^ per well) were inoculated in six-well plates after trypsinization and grown overnight. The cells were scraped with a sterile micropipette tip. At the indicated time point (0, 24 h), three different fields of each wound were randomly photographed using a light microscope. The wound area was measured by ImageJ software, set at 100% for 0 h, and the mean percentage of the total area was calculated.

### Cell migration and invasion assays

Assays were conducted using 8-mm Transwell chamber (Corning). The chambers were precoated with growth factor-reduced Matrigel (354234; Corning) and 200 µL of cell suspension in DMEM without FBS was placed in the upper chamber, with 10% FBS placed in the lower chamber. After incubation for 24 h, the cells were fixed and stained as colony formation assay and examined under an inverted light microscope, evaluating the cells in three random fields.

### Flow cytometry

A tube of digested, EDTA-free trypsin-treated OS cells was collected 48 h after transfection. Cells were treated with Annexin V/PI (Vazyme Biotech Co., Ltd, Nanjing, China) and analyzed on a flow cytometer (Beckman, USA).

### Western blotting

Proteins were extracted from cells with RIPA buffer with a protease and phosphatase inhibitor cocktail (Keygen, Nanjing, China). After the determination of concentrations using BCA, the proteins were separated on SDS-PAGE and transferred to nitrocellulose membranes. The blots were treated with anti-ITGB1 (1:1000) (ab30394, Abcam), anti-MMP2 (1:2000) (ab92536, Abcam), anti-AKT (1:1000) (4685, CST), anti-P-AKT (1:1000) (4228, CST), anti-PI3K (1:1000) (4249, CST), anti-P-PI3K (1:1000) (4228, CST), and anti-GAPDH (1:5000) (ab181602, Abcam). The bands were visualized using enhanced chemiluminescence. The loading control was GAPDH.

### Dual-luciferase reporter assay

HOS cells (2 × 10^4^/well) were seeded in 24-well plates and cultured until 80% confluent after which they were transiently transfected with 0.4 µg of an miR-134-5p mimic or miR-control (NC) and co-transfected with 0.1 µg of plasmid containing the 3′-UTRs of ITGB1 or their mutant (Genechem, Shanghai, China) using Lipofectamine 3000 (Invitrogen) for 48 h. Proteins were extracted and measured as above. Luciferase activity was assessed with a Dual-Luciferase Reporter Assay System (Promega Corp, Madison, USA) and a GLOMAX 20/20 luminometer (Promega). Transfection efficiency was determined by firefly/Renilla luciferase ratios.

### In vivo growth and bioimaging

Animal experiments were carried out in accordance with the Guide for the Care and Use of Laboratory Animals (Ministry of Science and Technology of China, 2006), and were approved by the animal ethics committee of Shanxi Medical University (approval number DW2022054). Five-week-old BALB/c nude mice were purchased from Charles River (China). Randomly selected mice received subcutaneous injections of 10^6^ lentivirus-transfected HOS cells (*n* = 6 per group). Researchers were not blinded to the group of the animals. Tumor growth was assessed each day. After 3 weeks, MMP activity was evaluated by MMPSense 680 (PerkinElmer) and fluorescence molecular tomography (FMT) (PerkinElmer). The mice were euthanized after 5 weeks, and the tumors were removed and measured [71]. Samples were retained in 10% formalin, RNAlater solution, or lysis buffer and stored at − 80 °C.

### Bioinformatics methods

#### Data collection

The functions of miR-134-5p in OS were examined using bioinformatics. Information on gene expression and patient characteristics was acquired from the TARGET database. Information on gene expression in 396 normal tissues was obtained from the Genotype-Tissue Expression (GTEx) database. The TARGET and GTEx datasets were combined and batch-to-batch variations were eliminated using the “sva” package in R. The RNA-seq data was converted from fragment per kilobase million to transcripts per million before analysis, and log2(x + 1) conversions were performed. The detailed information on 88 patients with OS is presented in Table S3. As described in our previous study [27], 48 VM-related genes were found to be expressed in the GTEx-TARGET dataset, and they are listed in Table S4. Potential miR-134-5p targets were predicted by miRWalk 3.0 using the criteria of scores >0.95 or experimental validation. Finally, 2886 candidate target genes were selected (Table S5).

#### Differential expression determination

The “limma” package was used to detect differentially expressed genes (DEGs) with the criteria of log2 |fold change (FC)| ≥1 and *p* value <0.05 in the GTEx-TARGET cohort. Furthermore, Kaplan–Meier curves were compiled using the “survival” and “survminer” packages.

#### Protein–protein interaction (PPI) networks

PPIs were created by STRING version 11.5 [72] using combined scores of 0.4 (medium confidence).

#### Functional enrichment analysis

DEG-enriched pathways were investigated using GO [73] and KEGG [74] enrichment analyses in the clusterProfiler package [75] using a false-discovery threshold of <0.05.

#### Gene set enrichment analysis (GSEA)

Differences in pathways and functions were assessed by GSEA [76] in the gene set “c2.cp.kegg.v7.4.symbols.gmt” from The Molecular Signatures Database (MSigDB), using *P* < 0.05 as the significance threshold.

### Statistical analysis

All data were analyzed using the GraphPad Prism v7.0 software. Data were presented as mean ± standard deviation (SD). All in vitro experiments were repeated three times. The comparisons between experimental and control groups were made by unpaired Student’s *t*-test or one-way ANOVA with Tukey’s test. Pearson correlation analyses were used for assessing relationships between genes. All *P* values were two-sided, and *p* < 0.05 was the significance threshold.

### Supplementary information

Supplemental Tables

Supplemental Figures legends

Fig. S1 Knockdown of miR-134-5p promotes the VM, proliferation, migration and invasion of OS.

Fig. S2 Bioinformatics analysis of the characteristics of seven candidate target genes of miR-134-5p.

Fig. S3 Gain-of-function analyses verifying the involvement of the miR-134-5p/ITGB1/MMP2 axis in OS VM, proliferation, migration, and invasion.

Fig. S4 Toxicity observation.

Original western blots

## Data Availability

The data that support the findings of this study are available from the corresponding author upon reasonable request.
