# Supplementary material for: MiR-134-5p inhibits the malignant phenotypes of osteosarcoma via ITGB1/MMP2/PI3K/Akt pathway
Source: Cell Death Discov. 2024 Apr 25;10:193. doi: 10.1038/s41420-024-01946-z (PMC11045734; doi:10.1038/s41420-024-01946-z)
Supplement: Supplementary file 1 — Supplemental Tables [file 41420_2024_1946_MOESM1_ESM.docx]

**Table S1.** The clinicopathological characteristics of patients with osteosarcoma.

| Parameters | Group | Patients | |
| --- | --- | --- | --- |
|  |  | No. | % |
| Gender | male | 3 | 37.5 |
|  | female | 5 | 62.5 |
| Age (years) | ≤14 | 6 | 75 |
|  | ＞14 | 2 | 25 |
| Subtype | Osteoblastic | 4 | 50 |
|  | Chondroblastic | 2 | 25 |
|  | other | 2 | 25 |
| Clinical stage | Ⅰ | 2 | 25 |
|  | Ⅱ | 3 | 37.5 |
|  | Ⅲ | 3 | 37.5 |
| Tumor size (cm) | ≤ 5 | 3 | 37.5 |
|  | ＞ 5 | 5 | 62.5 |
| Location | Femur | 5 | 62.5 |
|  | Tibia | 2 | 25 |
|  | Arm | 1 | 12.5 |

**Table S2**. Primers used for real time RT-PCR

| Primer | Sequence |
| --- | --- |
| 18S | Forward: 5'-AAACGGCTACCACATCCA-3'  Reverse: 5'-CACCACTTGCCCCTCCA-3' |
| U6 | Forward: 5'-CTCGCTTCGGCAGCACA-3'  Reverse: 5'-AACGCTTCACGAATTTGCGT-3' |
| Has-miR-134-5p | Forward: 5'-GCAGATTGTGACTGGTTGACC-3'  Reverse: 5'-GTGCAGGGTCCGAGGT-3' |
| ITGB1 | Forward: 5'-ATCCCAGAGGCTCCAAAGAT-3'  Reverse: 5'-CCCCTGATCTTAATCGCAAA-3' |
| LOX | Forward: 5'-CTGCTGCTGCGTGACAAC-3'  Reverse: 5'-TCAACCAAAGAGCGGCAG-3' |
| MMP2 | Forward: 5'-TACAGGATCATTGGCTACACACC-3'  Reverse: 5'-GGTCACATCGCTCCAGACT-3' |
| PDGFRB | Forward: 5'-AGCACCTTCGTTCTGACCTG-3'  Reverse: 5'-TATTCTCCCGTGTCTAGCCCA-3' |
| PTK2 | Forward: 5'-AGAGAATCCAGCTTTGGCTG-3'  Reverse: 5'-CAGCACTCGCGTATCTGGAG-3' |
| VEGFA | Fordward: 5'-CCTCCGAAACCATGAACTTT-3'  Reverse: 5'-TTCTTTGGTCTGCATTCACATT-3' |
| WNT5A | Fordward: 5'-GGGAGGTTGGCTTGAACATA-3'  Reverse: 5'-GAATAGGCACGCAATTACCTT-3' |

**Table S3: Clinical information of 88 osteosarcoma patients**

| **ID** | **futime** | **fustat** | **Age** | **Gender** | **Race** |
| --- | --- | --- | --- | --- | --- |
| TARGET-40-PAMHYN-01A | 3900 | 0 | 9.6 | Female | Asian |
| TARGET-40-PANZHX-01A | 1323 | 0 | 14.0 | Female | Asian |
| TARGET-40-PARFTG-01A | 653 | 1 | 13.7 | Female | Asian |
| TARGET-40-PAMTCM-01A | 3002 | 0 | 13.5 | Female | Black or African American |
| TARGET-40-PAPXGT-01A | 2526 | 0 | 11.4 | Female | Black or African American |
| TARGET-40-PAUVUL-01A | 347 | 1 | 5.6 | Female | Black or African American |
| TARGET-40-PAVCLP-01A | 511 | 0 | 11.1 | Female | Black or African American |
| TARGET-40-PALECC-01A | 754 | 1 | 10.0 | Female | Unknown |
| TARGET-40-PAMRHD-01A | 74 | 1 | 10.8 | Female | Unknown |
| TARGET-40-PARBGW-01A | 286 | 0 | 12.7 | Female | Unknown |
| TARGET-40-PASYUK-01A | 1870 | 0 | 16.2 | Female | Unknown |
| TARGET-40-PATMXR-01A | 386 | 1 | 20.9 | Female | Unknown |
| TARGET-40-PATUXZ-01A | 510 | 1 | 17.4 | Female | Unknown |
| TARGET-40-PAUXPZ-01A | 606 | 1 | 12.3 | Female | Unknown |
| TARGET-40-PAKXLD-01A | 2462 | 0 | 14.4 | Female | White |
| TARGET-40-PALFYN-01A | 1003 | 1 | 13.3 | Female | White |
| TARGET-40-PALKDP-01A | 3282 | 0 | 9.3 | Female | White |
| TARGET-40-PALWWX-01A | 2163 | 0 | 12.2 | Female | White |
| TARGET-40-PAMLKS-01A | 180 | 1 | 12.4 | Female | White |
| TARGET-40-PANVJJ-01A | 3095 | 0 | 13.8 | Female | White |
| TARGET-40-PAPIJR-01A | 1719 | 0 | 12.6 | Female | White |
| TARGET-40-PAPNVD-01A | 922 | 1 | 14.5 | Female | White |
| TARGET-40-PAPWWC-01A | 2520 | 0 | 9.8 | Female | White |
| TARGET-40-PARJXU-01A | 1631 | 1 | 12.7 | Female | White |
| TARGET-40-PASFCV-01A | 2062 | 0 | 10.5 | Female | White |
| TARGET-40-PASKZZ-01A | 542 | 0 | 13.2 | Female | White |
| TARGET-40-PASRNE-01A | 1707 | 0 | 3.6 | Female | White |
| TARGET-40-PASUUH-01A | 1625 | 0 | 14.1 | Female | White |
| TARGET-40-PATMIF-01A | 1580 | 0 | 12.0 | Female | White |
| TARGET-40-PAUBIT-01A | 1029 | 0 | 12.4 | Female | White |
| TARGET-40-PAUTYB-01A | 537 | 1 | 12.1 | Female | White |
| TARGET-40-PAUYTT-01A | 579 | 0 | 18.4 | Female | White |
| TARGET-40-PAVECB-01A | 476 | 0 | 9.0 | Female | White |
| TARGET-40-0A4I4M-01A | 708 | 0 | 18.9 | Female | Asian |
| TARGET-40-0A4I0Q-01A | 519 | 1 | 19.9 | Female | Unknown |
| TARGET-40-0A4I42-01A | 619 | 1 | 17.2 | Female | Unknown |
| TARGET-40-0A4I48-01A | 1616 | 0 | 16.0 | Female | Unknown |
| TARGET-40-PAKUZU-01A | 0 | 0 | 19.9 | Male | Asian |
| TARGET-40-PAMEKS-01A | 857 | 1 | 10.8 | Male | Asian |
| TARGET-40-PALHRL-01A | 4374 | 0 | 16.5 | Male | Black or African American |
| TARGET-40-PAMJXS-01A | 3862 | 1 | 11.2 | Male | Black or African American |
| TARGET-40-PAKFVX-01A | 922 | 0 | 14.8 | Male | Unknown |
| TARGET-40-PATMPU-01A | 1579 | 1 | 13.9 | Male | Unknown |
| TARGET-40-PAUUML-01A | 672 | 0 | 6.0 | Male | Unknown |
| TARGET-40-PAKZZK-01A | 1515 | 1 | 13.3 | Male | White |
| TARGET-40-PALKGN-01A | 2122 | 0 | 17.0 | Male | White |
| TARGET-40-PALZGU-01A | 2882 | 1 | 15.4 | Male | White |
| TARGET-40-PAMHLF-01A | 1913 | 0 | 8.9 | Male | White |
| TARGET-40-PAMYYJ-01A | 271 | 1 | 17.7 | Male | White |
| TARGET-40-PANGPE-01A | 3378 | 0 | 13.1 | Male | White |
| TARGET-40-PANGRW-01A | 3378 | 0 | 16.7 | Male | White |
| TARGET-40-PANMIG-01A | 776 | 1 | 14.7 | Male | White |
| TARGET-40-PANPUM-01A | 295 | 1 | 16.4 | Male | White |
| TARGET-40-PANSEN-01A | 2256 | 0 | 14.3 | Male | White |
| TARGET-40-PANXSC-01A | 1217 | 0 | 15.2 | Male | White |
| TARGET-40-PANZZJ-01A | 1538 | 0 | 18.8 | Male | White |
| TARGET-40-PAPFLB-01A | Unknown | Unknown | 15.1 | Male | White |
| TARGET-40-PAPKWD-01A | 422 | 1 | 9.5 | Male | White |
| TARGET-40-PARDAX-01A | 679 | 1 | 10.1 | Male | White |
| TARGET-40-PARGTM-01A | 2610 | 0 | 16.2 | Male | White |
| TARGET-40-PARKAF-01A | 1061 | 1 | 16.7 | Male | White |
| TARGET-40-PASEBY-01A | 2049 | 0 | 11.6 | Male | White |
| TARGET-40-PASEFS-01A | 2112 | 0 | 18.2 | Male | White |
| TARGET-40-PASNZV-01A | 1747 | 0 | 16.3 | Male | White |
| TARGET-40-PASSLM-01A | 1865 | 0 | 9.7 | Male | White |
| TARGET-40-PATAWV-01A | 1702 | 0 | 15.5 | Male | White |
| TARGET-40-PATEEM-01A | 1451 | 0 | 16.1 | Male | White |
| TARGET-40-PATJVI-01A | 1575 | 0 | 15.8 | Male | White |
| TARGET-40-PATKSS-01A | 758 | 1 | 13.4 | Male | White |
| TARGET-40-PATPBS-01A | 1468 | 0 | 15.1 | Male | White |
| TARGET-40-PAUTWB-01A | 687 | 0 | 13.9 | Male | White |
| TARGET-40-PAVALD-01A | 551 | 0 | 16.2 | Male | White |
| TARGET-40-PAVDTY-01A | 369 | 0 | 13.4 | Male | White |
| TARGET-40-0A4I65-01A | 5840 | 0 | 17.9 | Male | Unknown |
| TARGET-40-0A4I9K-01A | 2257 | 0 | 22.6 | Male | Unknown |
| TARGET-40-0A4I0W-01A | 683 | 0 | 20.1 | Male | Asian |
| TARGET-40-0A4I3S-01A | 1844 | 0 | 22.8 | Male | Black or African American |
| TARGET-40-0A4HMC-01A | 377 | 0 | 28.0 | Male | White |
| TARGET-40-0A4HX8-01A | 1750 | 0 | 29.1 | Male | White |
| TARGET-40-0A4HXS-01A | 2948 | 0 | 23.2 | Male | White |
| TARGET-40-0A4I4O-01A | 627 | 1 | 32.4 | Male | White |
| TARGET-40-0A4HLD-01A | 3946 | 0 | 19.9 | Male | Unknown |
| TARGET-40-0A4HY5-01A | 290 | 1 | 19.9 | Male | Unknown |
| TARGET-40-0A4I4E-01A | Unknown | Unknown | 16.4 | Male | Unknown |
| TARGET-40-0A4I5B-01A | 619 | 0 | 20.9 | Male | Unknown |
| TARGET-40-0A4I6O-01A | 1906 | 1 | 18.5 | Male | Unknown |
| TARGET-40-0A4I8U-01A | 983 | 0 | 20.1 | Male | Unknown |
| TARGET-40-0A4I0S-01A | 603 | 0 | Unknown | Unknown | Unknown |

**Table S4: Summary of 48 vasculogenic mimicry related genes**

| **Gene** | **Type** |
| --- | --- |
| ANGPT2 | vasculogenic mimicry |
| TFAP2A | vasculogenic mimicry |
| BCL2 | vasculogenic mimicry |
| BMI1 | vasculogenic mimicry |
| BMP4 | vasculogenic mimicry |
| PROM1 | vasculogenic mimicry |
| CDH5 | vasculogenic mimicry |
| DSG2 | vasculogenic mimicry |
| CDH1 | vasculogenic mimicry |
| EPHA2 | vasculogenic mimicry |
| PTK2 | vasculogenic mimicry |
| HIF1A | vasculogenic mimicry |
| ITGB1 | vasculogenic mimicry |
| LAMC2 | vasculogenic mimicry |
| LOX | vasculogenic mimicry |
| LOXL2 | vasculogenic mimicry |
| MACC1 | vasculogenic mimicry |
| MIF | vasculogenic mimicry |
| MMP14 | vasculogenic mimicry |
| MMP2 | vasculogenic mimicry |
| MMP9 | vasculogenic mimicry |
| MTOR | vasculogenic mimicry |
| NODAL | vasculogenic mimicry |
| NRP1 | vasculogenic mimicry |
| PDGFRB | vasculogenic mimicry |
| PECAM1 | vasculogenic mimicry |
| SERPINF1 | vasculogenic mimicry |
| RHOC | vasculogenic mimicry |
| SNAI2 | vasculogenic mimicry |
| SNAI1 | vasculogenic mimicry |
| STAT3 | vasculogenic mimicry |
| TGFB1 | vasculogenic mimicry |
| TIMP2 | vasculogenic mimicry |
| TNF | vasculogenic mimicry |
| TWIST1 | vasculogenic mimicry |
| TWIST2 | vasculogenic mimicry |
| VEGFA | vasculogenic mimicry |
| FLT1 | vasculogenic mimicry |
| KDR | vasculogenic mimicry |
| FLT4 | vasculogenic mimicry |
| VIM | vasculogenic mimicry |
| VCL | vasculogenic mimicry |
| WNT3A | vasculogenic mimicry |
| WNT5A | vasculogenic mimicry |
| YAP1 | vasculogenic mimicry |
| ZEB1 | vasculogenic mimicry |
| ZEB2 | vasculogenic mimicry |
| ACTA2 | vasculogenic mimicry |

**Table S5:Predicted target genes of miR-134-5p by miRWalk 3.0**

| **Gene** | **Type** | |
| --- | --- | --- |
| AMOTL1 | Target gene of miR-134-5p | |
| BCL2 | Target gene of miR-134-5p | |
| CD34 | Target gene of miR-134-5p | |
| CEACAM1 | Target gene of miR-134-5p | |
| DDAH1 | Target gene of miR-134-5p | |
| ID1 | Target gene of miR-134-5p | |
| IL6 | Target gene of miR-134-5p | |
| ITGB1 | Target gene of miR-134-5p | |
| MMP2 | Target gene of miR-134-5p | |
| NR2C2 | Target gene of miR-134-5p | |
| STMN1 | Target gene of miR-134-5p | |
| VEGFA | Target gene of miR-134-5p | |
| WNT5A | Target gene of miR-134-5p | |
| YAP1 | Target gene of miR-134-5p | |
| DEPTOR | Target gene of miR-134-5p | |
| FGFR2 | Target gene of miR-134-5p | |
| FOXM1 | Target gene of miR-134-5p | |
| HSP90AA1 | Target gene of miR-134-5p | |
| LEP | Target gene of miR-134-5p | |
| MACC1 | Target gene of miR-134-5p | |
| NR3C2 | Target gene of miR-134-5p | |
| PDGFRB | Target gene of miR-134-5p | |
| PKM | Target gene of miR-134-5p | |
| PRRX1 | Target gene of miR-134-5p |  |
| PUM2 | Target gene of miR-134-5p |  |
| SEMA4D | Target gene of miR-134-5p |  |
| SOX7 | Target gene of miR-134-5p |  |
| TP53INP1 | Target gene of miR-134-5p |  |
| WT1 | Target gene of miR-134-5p |  |
| AAK1 | Target gene of miR-134-5p |  |
| ACER3 | Target gene of miR-134-5p |  |
| ACOT11 | Target gene of miR-134-5p |  |
| ACOT9 | Target gene of miR-134-5p |  |
| ACSL3 | Target gene of miR-134-5p |  |
| ACSS1 | Target gene of miR-134-5p |  |
| ADAMDEC1 | Target gene of miR-134-5p |  |
| ADAMTS6 | Target gene of miR-134-5p |  |
| ADAP2 | Target gene of miR-134-5p |  |
| ADAT1 | Target gene of miR-134-5p |  |
| ADD1 | Target gene of miR-134-5p |  |
| ADD2 | Target gene of miR-134-5p |  |
| ADORA2A | Target gene of miR-134-5p |  |
| ADRA2A | Target gene of miR-134-5p |  |
| AFG3L2 | Target gene of miR-134-5p |  |
| AGBL3 | Target gene of miR-134-5p |  |
| AGBL4 | Target gene of miR-134-5p |  |
| AGK | Target gene of miR-134-5p |  |
| AGPAT4 | Target gene of miR-134-5p |  |
| AGPAT5 | Target gene of miR-134-5p | |
| AHCY | Target gene of miR-134-5p | |
| AHCYL1 | Target gene of miR-134-5p | |
| AHCYL2 | Target gene of miR-134-5p | |
| AHSG | Target gene of miR-134-5p | |
| AICDA | Target gene of miR-134-5p | |
| AIMP1 | Target gene of miR-134-5p | |
| AIPL1 | Target gene of miR-134-5p | |
| AK4 | Target gene of miR-134-5p | |
| AK5 | Target gene of miR-134-5p | |
| AKAP10 | Target gene of miR-134-5p | |
| AKT1 | Target gene of miR-134-5p | |
| ALDH16A1 | Target gene of miR-134-5p | |
| ALDH18A1 | Target gene of miR-134-5p | |
| ALDH3B2 | Target gene of miR-134-5p | |
| ALDH6A1 | Target gene of miR-134-5p | |
| AMMECR1L | Target gene of miR-134-5p | |
| AMOTL2 | Target gene of miR-134-5p | |
| ANAPC11 | Target gene of miR-134-5p | |
| ANK3 | Target gene of miR-134-5p | |
| ANKFY1 | Target gene of miR-134-5p | |
| ANKRD62 | Target gene of miR-134-5p | |
| ANO6 | Target gene of miR-134-5p | |
| AP1G1 | Target gene of miR-134-5p | |
| AP4S1 | Target gene of miR-134-5p | |
| APBB2 | Target gene of miR-134-5p | |
| APH1A | Target gene of miR-134-5p | |
| API5 | Target gene of miR-134-5p | |
| APOL6 | Target gene of miR-134-5p | |
| APPBP2 | Target gene of miR-134-5p | |
| AQP10 | Target gene of miR-134-5p | |
| ARCN1 | Target gene of miR-134-5p | |
| ARHGAP30 | Target gene of miR-134-5p | |
| ARHGDIB | Target gene of miR-134-5p | |
| ARHGEF37 | Target gene of miR-134-5p | |
| ARID5B | Target gene of miR-134-5p | |
| ARIH1 | Target gene of miR-134-5p | |
| ARIH2 | Target gene of miR-134-5p | |
| ARL1 | Target gene of miR-134-5p | |
| ARL5B | Target gene of miR-134-5p | |
| ARL8B | Target gene of miR-134-5p | |
| ARRDC4 | Target gene of miR-134-5p | |
| ARSA | Target gene of miR-134-5p | |
| ART4 | Target gene of miR-134-5p | |
| ASAP2 | Target gene of miR-134-5p | |
| ASB7 | Target gene of miR-134-5p | |
| ASCC1 | Target gene of miR-134-5p | |
| ASCC3 | Target gene of miR-134-5p | |
| ASPH | Target gene of miR-134-5p | |
| ASXL1 | Target gene of miR-134-5p | |
| ATF5 | Target gene of miR-134-5p | |
| ATG4D | Target gene of miR-134-5p | |
| ATP1B2 | Target gene of miR-134-5p | |
| ATP2A2 | Target gene of miR-134-5p | |
| ATP2B3 | Target gene of miR-134-5p | |
| ATP2B4 | Target gene of miR-134-5p | |
| ATP6V0A1 | Target gene of miR-134-5p | |
| ATP6V0D1 | Target gene of miR-134-5p | |
| ATP8B2 | Target gene of miR-134-5p | |
| ATP8B3 | Target gene of miR-134-5p | |
| ATP8B4 | Target gene of miR-134-5p | |
| ATXN10 | Target gene of miR-134-5p | |
| ATXN7L3 | Target gene of miR-134-5p | |
| AUTS2 | Target gene of miR-134-5p | |
| AVL9 | Target gene of miR-134-5p | |
| AZIN1 | Target gene of miR-134-5p | |
| B3GALNT2 | Target gene of miR-134-5p | |
| B4GALT6 | Target gene of miR-134-5p | |
| BARX2 | Target gene of miR-134-5p | |
| BCAP29 | Target gene of miR-134-5p | |
| BCL2L15 | Target gene of miR-134-5p | |
| BEAN1 | Target gene of miR-134-5p | |
| BEND2 | Target gene of miR-134-5p | |
| BGN | Target gene of miR-134-5p | |
| BIRC6 | Target gene of miR-134-5p | |
| BMI1 | Target gene of miR-134-5p | |
| BMP7 | Target gene of miR-134-5p | |
| BMS1 | Target gene of miR-134-5p | |
| BRAF | Target gene of miR-134-5p | |
| BRWD1 | Target gene of miR-134-5p | |
| BTG2 | Target gene of miR-134-5p | |
| BTN3A1 | Target gene of miR-134-5p | |
| BTRC | Target gene of miR-134-5p | |
| BUB3 | Target gene of miR-134-5p | |
| BZW1 | Target gene of miR-134-5p | |
| C10orf82 | Target gene of miR-134-5p | |
| C10orf88 | Target gene of miR-134-5p | |
| C15orf62 | Target gene of miR-134-5p | |
| C17orf80 | Target gene of miR-134-5p | |
| C20orf203 | Target gene of miR-134-5p | |
| C2orf68 | Target gene of miR-134-5p | |
| C2orf88 | Target gene of miR-134-5p | |
| C3orf18 | Target gene of miR-134-5p | |
| C5AR1 | Target gene of miR-134-5p | |
| CA12 | Target gene of miR-134-5p | |
| CABLES2 | Target gene of miR-134-5p | |
| CABP4 | Target gene of miR-134-5p | |
| CADM1 | Target gene of miR-134-5p | |
| CARD8 | Target gene of miR-134-5p | |
| CBFB | Target gene of miR-134-5p | |
| CCDC102A | Target gene of miR-134-5p | |
| CCDC134 | Target gene of miR-134-5p | |
| CCDC141 | Target gene of miR-134-5p | |
| CCDC149 | Target gene of miR-134-5p | |
| CCDC69 | Target gene of miR-134-5p | |
| CCDC77 | Target gene of miR-134-5p | |
| CCDC85C | Target gene of miR-134-5p | |
| CCND2 | Target gene of miR-134-5p | |
| CCR1 | Target gene of miR-134-5p | |
| CCR6 | Target gene of miR-134-5p | |
| CD164 | Target gene of miR-134-5p | |
| CD226 | Target gene of miR-134-5p | |
| CD46 | Target gene of miR-134-5p | |
| CD84 | Target gene of miR-134-5p | |
| CD8B | Target gene of miR-134-5p | |
| CDC37L1 | Target gene of miR-134-5p | |
| CDC42BPA | Target gene of miR-134-5p | |
| CDKL5 | Target gene of miR-134-5p | |
| CDS1 | Target gene of miR-134-5p | |
| CELSR1 | Target gene of miR-134-5p | |
| CEP41 | Target gene of miR-134-5p | |
| CEP78 | Target gene of miR-134-5p | |
| CERCAM | Target gene of miR-134-5p | |
| CERS6 | Target gene of miR-134-5p | |
| CHM | Target gene of miR-134-5p | |
| CHMP1B | Target gene of miR-134-5p | |
| CHORDC1 | Target gene of miR-134-5p | |
| CHRNB2 | Target gene of miR-134-5p | |
| CHST3 | Target gene of miR-134-5p | |
| CHST9 | Target gene of miR-134-5p | |
| CIB2 | Target gene of miR-134-5p | |
| CIITA | Target gene of miR-134-5p | |
| CILP | Target gene of miR-134-5p | |
| CKAP5 | Target gene of miR-134-5p | |
| CLCN4 | Target gene of miR-134-5p | |
| CLDN1 | Target gene of miR-134-5p | |
| CLEC1B | Target gene of miR-134-5p | |
| CMTM4 | Target gene of miR-134-5p | |
| CMTM6 | Target gene of miR-134-5p | |
| CNST | Target gene of miR-134-5p | |
| COBL | Target gene of miR-134-5p | |
| COBLL1 | Target gene of miR-134-5p | |
| COG3 | Target gene of miR-134-5p | |
| COPA | Target gene of miR-134-5p | |
| COPS4 | Target gene of miR-134-5p | |
| COPS7B | Target gene of miR-134-5p | |
| COPZ1 | Target gene of miR-134-5p | |
| COQ6 | Target gene of miR-134-5p | |
| CORO2A | Target gene of miR-134-5p | |
| COX10 | Target gene of miR-134-5p | |
| COX11 | Target gene of miR-134-5p | |
| CPS1 | Target gene of miR-134-5p | |
| CREM | Target gene of miR-134-5p | |
| CRIPT | Target gene of miR-134-5p | |
| CRKL | Target gene of miR-134-5p | |
| CRYBA4 | Target gene of miR-134-5p | |
| CSH2 | Target gene of miR-134-5p | |
| CSNK1A1 | Target gene of miR-134-5p | |
| CSNK1E | Target gene of miR-134-5p | |
| CSNK1G1 | Target gene of miR-134-5p | |
| CSNK1G3 | Target gene of miR-134-5p | |
| CTBP2 | Target gene of miR-134-5p | |
| CTDSPL2 | Target gene of miR-134-5p | |
| CTPS2 | Target gene of miR-134-5p | |
| CTTNBP2NL | Target gene of miR-134-5p | |
| CUEDC1 | Target gene of miR-134-5p | |
| CUL5 | Target gene of miR-134-5p | |
| CUTC | Target gene of miR-134-5p | |
| CX3CR1 | Target gene of miR-134-5p | |
| CXCL14 | Target gene of miR-134-5p | |
| CXCL9 | Target gene of miR-134-5p | |
| CXCR5 | Target gene of miR-134-5p | |
| CYB5B | Target gene of miR-134-5p | |
| CYCS | Target gene of miR-134-5p | |
| CYP11B1 | Target gene of miR-134-5p | |
| CYP4F3 | Target gene of miR-134-5p | |
| DAB2IP | Target gene of miR-134-5p | |
| DAG1 | Target gene of miR-134-5p | |
| DAPK2 | Target gene of miR-134-5p | |
| DCAF17 | Target gene of miR-134-5p | |
| DCBLD2 | Target gene of miR-134-5p | |
| DCTD | Target gene of miR-134-5p | |
| DCX | Target gene of miR-134-5p | |
| DDX19B | Target gene of miR-134-5p | |
| DDX21 | Target gene of miR-134-5p | |
| DEDD | Target gene of miR-134-5p | |
| DENND3 | Target gene of miR-134-5p | |
| DENND5B | Target gene of miR-134-5p | |
| DGKH | Target gene of miR-134-5p | |
| DHDDS | Target gene of miR-134-5p | |
| DHH | Target gene of miR-134-5p | |
| DHPS | Target gene of miR-134-5p | |
| DHRS2 | Target gene of miR-134-5p | |
| DISC1 | Target gene of miR-134-5p | |
| DKC1 | Target gene of miR-134-5p | |
| DLGAP2 | Target gene of miR-134-5p | |
| DMBX1 | Target gene of miR-134-5p | |
| DMKN | Target gene of miR-134-5p | |
| DNAJA1 | Target gene of miR-134-5p | |
| DNASE1L3 | Target gene of miR-134-5p | |
| DNMT1 | Target gene of miR-134-5p | |
| DNMT3A | Target gene of miR-134-5p | |
| DOC2A | Target gene of miR-134-5p | |
| DOCK8 | Target gene of miR-134-5p | |
| DOK4 | Target gene of miR-134-5p | |
| DOK7 | Target gene of miR-134-5p | |
| DPAGT1 | Target gene of miR-134-5p | |
| DPF3 | Target gene of miR-134-5p | |
| DPP10 | Target gene of miR-134-5p | |
| DPP4 | Target gene of miR-134-5p | |
| DPP9 | Target gene of miR-134-5p | |
| DPYSL3 | Target gene of miR-134-5p | |
| DPYSL4 | Target gene of miR-134-5p | |
| DR1 | Target gene of miR-134-5p | |
| DSC2 | Target gene of miR-134-5p | |
| DSPP | Target gene of miR-134-5p | |
| DST | Target gene of miR-134-5p | |
| DSTN | Target gene of miR-134-5p | |
| DTWD1 | Target gene of miR-134-5p | |
| DYDC2 | Target gene of miR-134-5p | |
| DYNC1H1 | Target gene of miR-134-5p | |
| DYNC1LI2 | Target gene of miR-134-5p | |
| DYRK3 | Target gene of miR-134-5p | |
| EBF3 | Target gene of miR-134-5p | |
| ECHDC2 | Target gene of miR-134-5p | |
| ECSIT | Target gene of miR-134-5p | |
| EDEM1 | Target gene of miR-134-5p | |
| EFR3A | Target gene of miR-134-5p | |
| EGR3 | Target gene of miR-134-5p | |
| EIF2D | Target gene of miR-134-5p | |
| EIF2S1 | Target gene of miR-134-5p | |
| ELAVL4 | Target gene of miR-134-5p | |
| ELL2 | Target gene of miR-134-5p | |
| ELP4 | Target gene of miR-134-5p | |
| EMG1 | Target gene of miR-134-5p | |
| ENAM | Target gene of miR-134-5p | |
| ENDOV | Target gene of miR-134-5p | |
| ENPP5 | Target gene of miR-134-5p | |
| ENSA | Target gene of miR-134-5p | |
| ENTPD7 | Target gene of miR-134-5p | |
| EPB41L5 | Target gene of miR-134-5p | |
| EPOR | Target gene of miR-134-5p | |
| EPS15 | Target gene of miR-134-5p | |
| EPSTI1 | Target gene of miR-134-5p | |
| ERCC8 | Target gene of miR-134-5p | |
| ERGIC1 | Target gene of miR-134-5p | |
| ERLIN2 | Target gene of miR-134-5p | |
| ERP44 | Target gene of miR-134-5p | |
| ETV4 | Target gene of miR-134-5p | |
| ETV5 | Target gene of miR-134-5p | |
| EVC | Target gene of miR-134-5p | |
| EXOC4 | Target gene of miR-134-5p | |
| FAM110C | Target gene of miR-134-5p | |
| FAM124B | Target gene of miR-134-5p | |
| FAM136A | Target gene of miR-134-5p | |
| FAM13B | Target gene of miR-134-5p | |
| FAM169A | Target gene of miR-134-5p | |
| FAM43A | Target gene of miR-134-5p | |
| FAM78A | Target gene of miR-134-5p | |
| FAM8A1 | Target gene of miR-134-5p | |
| FAM91A1 | Target gene of miR-134-5p | |
| FAM9A | Target gene of miR-134-5p | |
| FASLG | Target gene of miR-134-5p | |
| FASTKD2 | Target gene of miR-134-5p | |
| FBXL20 | Target gene of miR-134-5p | |
| FBXO25 | Target gene of miR-134-5p | |
| FBXO28 | Target gene of miR-134-5p | |
| FBXO45 | Target gene of miR-134-5p | |
| FCN1 | Target gene of miR-134-5p | |
| FCRL5 | Target gene of miR-134-5p | |
| FER | Target gene of miR-134-5p | |
| FGD4 | Target gene of miR-134-5p | |
| FGD5 | Target gene of miR-134-5p | |
| FGF13 | Target gene of miR-134-5p | |
| FIP1L1 | Target gene of miR-134-5p | |
| FKBP9 | Target gene of miR-134-5p | |
| FLRT2 | Target gene of miR-134-5p | |
| FMO2 | Target gene of miR-134-5p | |
| FNBP1L | Target gene of miR-134-5p | |
| FNDC5 | Target gene of miR-134-5p | |
| FOXO4 | Target gene of miR-134-5p | |
| FYCO1 | Target gene of miR-134-5p | |
| GAB1 | Target gene of miR-134-5p | |
| GABRE | Target gene of miR-134-5p | |
| GABRQ | Target gene of miR-134-5p | |
| GALNT10 | Target gene of miR-134-5p | |
| GAPT | Target gene of miR-134-5p | |
| GBP2 | Target gene of miR-134-5p | |
| GDAP1 | Target gene of miR-134-5p | |
| GDAP2 | Target gene of miR-134-5p | |
| GDF7 | Target gene of miR-134-5p | |
| GEM | Target gene of miR-134-5p | |
| GEMIN8 | Target gene of miR-134-5p | |
| GFPT1 | Target gene of miR-134-5p | |
| GFRA3 | Target gene of miR-134-5p | |
| GHRHR | Target gene of miR-134-5p | |
| GJA5 | Target gene of miR-134-5p | |
| GLRX | Target gene of miR-134-5p | |
| GMDS | Target gene of miR-134-5p | |
| GMEB2 | Target gene of miR-134-5p | |
| GMFB | Target gene of miR-134-5p | |
| GNA12 | Target gene of miR-134-5p | |
| GNAI3 | Target gene of miR-134-5p | |
| GNB5 | Target gene of miR-134-5p | |
| GNG7 | Target gene of miR-134-5p | |
| GNL1 | Target gene of miR-134-5p | |
| GOLIM4 | Target gene of miR-134-5p | |
| GORASP2 | Target gene of miR-134-5p | |
| GPC6 | Target gene of miR-134-5p | |
| GPR141 | Target gene of miR-134-5p | |
| GPR68 | Target gene of miR-134-5p | |
| GPRC5A | Target gene of miR-134-5p | |
| GRAMD1B | Target gene of miR-134-5p | |
| GRM7 | Target gene of miR-134-5p | |
| GSPT1 | Target gene of miR-134-5p | |
| GSTA4 | Target gene of miR-134-5p | |
| GUCA1B | Target gene of miR-134-5p | |
| GUCA2B | Target gene of miR-134-5p | |
| GZMK | Target gene of miR-134-5p | |
| HAND2 | Target gene of miR-134-5p | |
| HDAC2 | Target gene of miR-134-5p | |
| HIGD1A | Target gene of miR-134-5p | |
| HIP1 | Target gene of miR-134-5p | |
| HIRIP3 | Target gene of miR-134-5p | |
| HLA-DOA | Target gene of miR-134-5p | |
| HMG20A | Target gene of miR-134-5p | |
| HMGB3 | Target gene of miR-134-5p | |
| HMGCLL1 | Target gene of miR-134-5p | |
| HMGCR | Target gene of miR-134-5p | |
| HOXA4 | Target gene of miR-134-5p | |
| HOXC13 | Target gene of miR-134-5p | |
| HPGDS | Target gene of miR-134-5p | |
| HRH1 | Target gene of miR-134-5p | |
| HS3ST3A1 | Target gene of miR-134-5p | |
| HSF5 | Target gene of miR-134-5p | |
| HSPA4L | Target gene of miR-134-5p | |
| HSPA9 | Target gene of miR-134-5p | |
| HTR6 | Target gene of miR-134-5p | |
| ICOS | Target gene of miR-134-5p | |
| ICOSLG | Target gene of miR-134-5p | |
| IDH1 | Target gene of miR-134-5p | |
| IDS | Target gene of miR-134-5p | |
| IFI44L | Target gene of miR-134-5p | |
| IFIT1 | Target gene of miR-134-5p | |
| IKBKG | Target gene of miR-134-5p | |
| IL12RB2 | Target gene of miR-134-5p | |
| IL16 | Target gene of miR-134-5p | |
| IL1RAP | Target gene of miR-134-5p | |
| IL6ST | Target gene of miR-134-5p | |
| IMMP2L | Target gene of miR-134-5p | |
| INA | Target gene of miR-134-5p | |
| INSIG2 | Target gene of miR-134-5p | |
| INSM2 | Target gene of miR-134-5p | |
| INTS4 | Target gene of miR-134-5p | |
| INTS7 | Target gene of miR-134-5p | |
| IPCEF1 | Target gene of miR-134-5p | |
| IPO5 | Target gene of miR-134-5p | |
| IPO8 | Target gene of miR-134-5p | |
| IRAK3 | Target gene of miR-134-5p | |
| IRS1 | Target gene of miR-134-5p | |
| ISX | Target gene of miR-134-5p | |
| ITGB1BP1 | Target gene of miR-134-5p | |
| ITGB8 | Target gene of miR-134-5p | |
| ITGBL1 | Target gene of miR-134-5p | |
| ITPKB | Target gene of miR-134-5p | |
| JAGN1 | Target gene of miR-134-5p | |
| JMJD8 | Target gene of miR-134-5p | |
| JRK | Target gene of miR-134-5p | |
| KBTBD2 | Target gene of miR-134-5p | |
| KCNAB2 | Target gene of miR-134-5p | |
| KCNJ1 | Target gene of miR-134-5p | |
| KCNJ15 | Target gene of miR-134-5p | |
| KCNMA1 | Target gene of miR-134-5p | |
| KCNV1 | Target gene of miR-134-5p | |
| KCTD5 | Target gene of miR-134-5p | |
| KDELR2 | Target gene of miR-134-5p | |
| KDM5B | Target gene of miR-134-5p | |
| KIAA0040 | Target gene of miR-134-5p | |
| KIAA0319 | Target gene of miR-134-5p | |
| KIF15 | Target gene of miR-134-5p | |
| KIF18B | Target gene of miR-134-5p | |
| KIF5A | Target gene of miR-134-5p | |
| KLF14 | Target gene of miR-134-5p | |
| KLHDC10 | Target gene of miR-134-5p | |
| KMO | Target gene of miR-134-5p | |
| KRAS | Target gene of miR-134-5p | |
| KREMEN1 | Target gene of miR-134-5p | |
| KRT73 | Target gene of miR-134-5p | |
| KRT80 | Target gene of miR-134-5p | |
| KRTAP2-1 | Target gene of miR-134-5p | |
| KYNU | Target gene of miR-134-5p | |
| L2HGDH | Target gene of miR-134-5p | |
| LAIR1 | Target gene of miR-134-5p | |
| LARS2 | Target gene of miR-134-5p | |
| LAT2 | Target gene of miR-134-5p | |
| LCORL | Target gene of miR-134-5p | |
| LCP2 | Target gene of miR-134-5p | |
| LDB3 | Target gene of miR-134-5p | |
| LDLRAD3 | Target gene of miR-134-5p | |
| LENG8 | Target gene of miR-134-5p | |
| LGALS8 | Target gene of miR-134-5p | |
| LIF | Target gene of miR-134-5p | |
| LIMCH1 | Target gene of miR-134-5p | |
| LIMD1 | Target gene of miR-134-5p | |
| LIMD2 | Target gene of miR-134-5p | |
| LIPG | Target gene of miR-134-5p | |
| LLPH | Target gene of miR-134-5p | |
| LMBR1 | Target gene of miR-134-5p | |
| LMO3 | Target gene of miR-134-5p | |
| LOC389831 | Target gene of miR-134-5p | |
| LOX | Target gene of miR-134-5p | |
| LPCAT1 | Target gene of miR-134-5p | |
| LPO | Target gene of miR-134-5p | |
| LRP11 | Target gene of miR-134-5p | |
| LRRC20 | Target gene of miR-134-5p | |
| LRRC3 | Target gene of miR-134-5p | |
| LRRC59 | Target gene of miR-134-5p | |
| LRRK2 | Target gene of miR-134-5p | |
| LSM12 | Target gene of miR-134-5p | |
| LST1 | Target gene of miR-134-5p | |
| LUC7L3 | Target gene of miR-134-5p | |
| LYPD6 | Target gene of miR-134-5p | |
| LYPLAL1 | Target gene of miR-134-5p | |
| LZIC | Target gene of miR-134-5p | |
| LZTFL1 | Target gene of miR-134-5p | |
| MAD2L1 | Target gene of miR-134-5p | |
| MALT1 | Target gene of miR-134-5p | |
| MANBAL | Target gene of miR-134-5p | |
| MANEAL | Target gene of miR-134-5p | |
| MAP1B | Target gene of miR-134-5p | |
| MAP3K13 | Target gene of miR-134-5p | |
| MAP4 | Target gene of miR-134-5p | |
| MAP4K2 | Target gene of miR-134-5p | |
| MAPK1 | Target gene of miR-134-5p | |
| MAPK6 | Target gene of miR-134-5p | |
| MAPKAPK5 | Target gene of miR-134-5p | |
| MCCC2 | Target gene of miR-134-5p | |
| MCF2L2 | Target gene of miR-134-5p | |
| MCM9 | Target gene of miR-134-5p | |
| MCTP2 | Target gene of miR-134-5p | |
| MDM2 | Target gene of miR-134-5p | |
| MDM4 | Target gene of miR-134-5p | |
| METAP1D | Target gene of miR-134-5p | |
| METTL6 | Target gene of miR-134-5p | |
| MFAP3 | Target gene of miR-134-5p | |
| MFSD9 | Target gene of miR-134-5p | |
| MGMT | Target gene of miR-134-5p | |
| MGRN1 | Target gene of miR-134-5p | |
| MIOX | Target gene of miR-134-5p | |
| MLX | Target gene of miR-134-5p | |
| MMACHC | Target gene of miR-134-5p | |
| MPI | Target gene of miR-134-5p | |
| MPPED2 | Target gene of miR-134-5p | |
| MPZL3 | Target gene of miR-134-5p | |
| MR1 | Target gene of miR-134-5p | |
| MRFAP1 | Target gene of miR-134-5p | |
| MRPL13 | Target gene of miR-134-5p | |
| MRPL42 | Target gene of miR-134-5p | |
| MRPS14 | Target gene of miR-134-5p | |
| MRPS23 | Target gene of miR-134-5p | |
| MRPS25 | Target gene of miR-134-5p | |
| MRPS26 | Target gene of miR-134-5p | |
| MRPS33 | Target gene of miR-134-5p | |
| MRPS6 | Target gene of miR-134-5p | |
| MRRF | Target gene of miR-134-5p | |
| MS4A2 | Target gene of miR-134-5p | |
| MTMR1 | Target gene of miR-134-5p | |
| MTMR12 | Target gene of miR-134-5p | |
| MTMR9 | Target gene of miR-134-5p | |
| MTX3 | Target gene of miR-134-5p | |
| MXD1 | Target gene of miR-134-5p | |
| MXRA7 | Target gene of miR-134-5p | |
| MYB | Target gene of miR-134-5p | |
| MYCBP | Target gene of miR-134-5p | |
| MYH10 | Target gene of miR-134-5p | |
| MYNN | Target gene of miR-134-5p | |
| MYO10 | Target gene of miR-134-5p | |
| MYO1G | Target gene of miR-134-5p | |
| MYO5C | Target gene of miR-134-5p | |
| MYO9A | Target gene of miR-134-5p | |
| MZB1 | Target gene of miR-134-5p | |
| N6AMT1 | Target gene of miR-134-5p | |
| NAA35 | Target gene of miR-134-5p | |
| NCK1 | Target gene of miR-134-5p | |
| NCKAP1 | Target gene of miR-134-5p | |
| NDFIP1 | Target gene of miR-134-5p | |
| NDUFA10 | Target gene of miR-134-5p | |
| NDUFA5 | Target gene of miR-134-5p | |
| NDUFAF4 | Target gene of miR-134-5p | |
| NDUFS1 | Target gene of miR-134-5p | |
| NEBL | Target gene of miR-134-5p | |
| NECAB3 | Target gene of miR-134-5p | |
| NEDD4L | Target gene of miR-134-5p | |
| NEK7 | Target gene of miR-134-5p | |
| NEO1 | Target gene of miR-134-5p | |
| NF2 | Target gene of miR-134-5p | |
| NFAM1 | Target gene of miR-134-5p | |
| NFATC2IP | Target gene of miR-134-5p | |
| NFIC | Target gene of miR-134-5p | |
| NFYA | Target gene of miR-134-5p | |
| NIT2 | Target gene of miR-134-5p | |
| NKAIN3 | Target gene of miR-134-5p | |
| NKAP | Target gene of miR-134-5p | |
| NME7 | Target gene of miR-134-5p | |
| NOD2 | Target gene of miR-134-5p | |
| NOL10 | Target gene of miR-134-5p | |
| NOL3 | Target gene of miR-134-5p | |
| NOVA2 | Target gene of miR-134-5p | |
| NPAS3 | Target gene of miR-134-5p | |
| NPRL3 | Target gene of miR-134-5p | |
| NR1D2 | Target gene of miR-134-5p | |
| NR2F2 | Target gene of miR-134-5p | |
| NRAS | Target gene of miR-134-5p | |
| NRBP2 | Target gene of miR-134-5p | |
| NRXN1 | Target gene of miR-134-5p | |
| NSA2 | Target gene of miR-134-5p | |
| NSDHL | Target gene of miR-134-5p | |
| NSL1 | Target gene of miR-134-5p | |
| NTPCR | Target gene of miR-134-5p | |
| NTRK2 | Target gene of miR-134-5p | |
| NTSR1 | Target gene of miR-134-5p | |
| NUDCD2 | Target gene of miR-134-5p | |
| NUDT15 | Target gene of miR-134-5p | |
| NUDT9 | Target gene of miR-134-5p | |
| OCRL | Target gene of miR-134-5p | |
| OGN | Target gene of miR-134-5p | |
| OPA3 | Target gene of miR-134-5p | |
| OPRK1 | Target gene of miR-134-5p | |
| OR1D2 | Target gene of miR-134-5p | |
| OR2F1 | Target gene of miR-134-5p | |
| OR5H1 | Target gene of miR-134-5p | |
| ORC3 | Target gene of miR-134-5p | |
| OSBPL9 | Target gene of miR-134-5p | |
| OTUD7B | Target gene of miR-134-5p | |
| OTX2 | Target gene of miR-134-5p | |
| P2RY8 | Target gene of miR-134-5p | |
| PACRGL | Target gene of miR-134-5p | |
| PADI1 | Target gene of miR-134-5p | |
| PAFAH1B2 | Target gene of miR-134-5p | |
| PAG1 | Target gene of miR-134-5p | |
| PALLD | Target gene of miR-134-5p | |
| PANK3 | Target gene of miR-134-5p | |
| PANX1 | Target gene of miR-134-5p | |
| PAQR9 | Target gene of miR-134-5p | |
| PARN | Target gene of miR-134-5p | |
| PARP11 | Target gene of miR-134-5p | |
| PARP9 | Target gene of miR-134-5p | |
| PATE1 | Target gene of miR-134-5p | |
| PATL1 | Target gene of miR-134-5p | |
| PBX1 | Target gene of miR-134-5p | |
| PCGF6 | Target gene of miR-134-5p | |
| PCYT1B | Target gene of miR-134-5p | |
| PDCD2 | Target gene of miR-134-5p | |
| PDE12 | Target gene of miR-134-5p | |
| PDE2A | Target gene of miR-134-5p | |
| PDLIM4 | Target gene of miR-134-5p | |
| PDLIM5 | Target gene of miR-134-5p | |
| PDP1 | Target gene of miR-134-5p | |
| PDPK1 | Target gene of miR-134-5p | |
| PDPR | Target gene of miR-134-5p | |
| PEA15 | Target gene of miR-134-5p | |
| PELO | Target gene of miR-134-5p | |
| PEX5L | Target gene of miR-134-5p | |
| PFKFB2 | Target gene of miR-134-5p | |
| PFKM | Target gene of miR-134-5p | |
| PGGT1B | Target gene of miR-134-5p | |
| PGK1 | Target gene of miR-134-5p | |
| PGM2L1 | Target gene of miR-134-5p | |
| PHF8 | Target gene of miR-134-5p | |
| PHTF2 | Target gene of miR-134-5p | |
| PIK3CG | Target gene of miR-134-5p | |
| PIM3 | Target gene of miR-134-5p | |
| PKP2 | Target gene of miR-134-5p | |
| PLA2G12B | Target gene of miR-134-5p | |
| PLA2G5 | Target gene of miR-134-5p | |
| PLAG1 | Target gene of miR-134-5p | |
| PLAUR | Target gene of miR-134-5p | |
| PLCG2 | Target gene of miR-134-5p | |
| PLEKHA2 | Target gene of miR-134-5p | |
| PLEKHA3 | Target gene of miR-134-5p | |
| PLEKHA8 | Target gene of miR-134-5p | |
| PLEKHB1 | Target gene of miR-134-5p | |
| PLEKHO2 | Target gene of miR-134-5p | |
| PLS1 | Target gene of miR-134-5p | |
| PLSCR3 | Target gene of miR-134-5p | |
| PLXNA4 | Target gene of miR-134-5p | |
| PMS2 | Target gene of miR-134-5p | |
| PNP | Target gene of miR-134-5p | |
| POC5 | Target gene of miR-134-5p | |
| POFUT1 | Target gene of miR-134-5p | |
| POLA2 | Target gene of miR-134-5p | |
| POLR2F | Target gene of miR-134-5p | |
| POLR3C | Target gene of miR-134-5p | |
| POU2F2 | Target gene of miR-134-5p | |
| PPFIBP2 | Target gene of miR-134-5p | |
| PPIH | Target gene of miR-134-5p | |
| PPIP5K2 | Target gene of miR-134-5p | |
| PPM1K | Target gene of miR-134-5p | |
| PPM1L | Target gene of miR-134-5p | |
| PPM1M | Target gene of miR-134-5p | |
| PPP1R12B | Target gene of miR-134-5p | |
| PPP1R17 | Target gene of miR-134-5p | |
| PPP1R7 | Target gene of miR-134-5p | |
| PPP1R9A | Target gene of miR-134-5p | |
| PREP | Target gene of miR-134-5p | |
| PRKAA1 | Target gene of miR-134-5p | |
| PRKAG3 | Target gene of miR-134-5p | |
| PRKCB | Target gene of miR-134-5p | |
| PRKCE | Target gene of miR-134-5p | |
| PRKG2 | Target gene of miR-134-5p | |
| PRKRA | Target gene of miR-134-5p | |
| PRMT7 | Target gene of miR-134-5p | |
| PRR5 | Target gene of miR-134-5p | |
| PRSS12 | Target gene of miR-134-5p | |
| PSEN1 | Target gene of miR-134-5p | |
| PSENEN | Target gene of miR-134-5p | |
| PSMB5 | Target gene of miR-134-5p | |
| PSMC3IP | Target gene of miR-134-5p | |
| PSME3 | Target gene of miR-134-5p | |
| PTCRA | Target gene of miR-134-5p | |
| PTGFR | Target gene of miR-134-5p | |
| PTGR1 | Target gene of miR-134-5p | |
| PTK2 | Target gene of miR-134-5p | |
| PTP4A1 | Target gene of miR-134-5p | |
| PTP4A3 | Target gene of miR-134-5p | |
| PTRHD1 | Target gene of miR-134-5p | |
| PTTG1IP | Target gene of miR-134-5p | |
| PUS7 | Target gene of miR-134-5p | |
| PUS7L | Target gene of miR-134-5p | |
| RAB11B | Target gene of miR-134-5p | |
| RAB15 | Target gene of miR-134-5p | |
| RAB1B | Target gene of miR-134-5p | |
| RAB25 | Target gene of miR-134-5p | |
| RAB2A | Target gene of miR-134-5p | |
| RAB34 | Target gene of miR-134-5p | |
| RAB3C | Target gene of miR-134-5p | |
| RAB3IL1 | Target gene of miR-134-5p | |
| RAB42 | Target gene of miR-134-5p | |
| RABGEF1 | Target gene of miR-134-5p | |
| RAET1E | Target gene of miR-134-5p | |
| RALGAPB | Target gene of miR-134-5p | |
| RALGPS2 | Target gene of miR-134-5p | |
| RANGRF | Target gene of miR-134-5p | |
| RAP1GDS1 | Target gene of miR-134-5p | |
| RAP2A | Target gene of miR-134-5p | |
| RAPGEF5 | Target gene of miR-134-5p | |
| RASEF | Target gene of miR-134-5p | |
| RASL10B | Target gene of miR-134-5p | |
| RASL12 | Target gene of miR-134-5p | |
| RASSF2 | Target gene of miR-134-5p | |
| RASSF6 | Target gene of miR-134-5p | |
| RBFOX2 | Target gene of miR-134-5p | |
| RBM19 | Target gene of miR-134-5p | |
| RBM23 | Target gene of miR-134-5p | |
| RBM38 | Target gene of miR-134-5p | |
| RBM41 | Target gene of miR-134-5p | |
| RBP1 | Target gene of miR-134-5p | |
| RCAN3 | Target gene of miR-134-5p | |
| RCHY1 | Target gene of miR-134-5p | |
| RDH11 | Target gene of miR-134-5p | |
| RDX | Target gene of miR-134-5p | |
| RECQL5 | Target gene of miR-134-5p | |
| REG3A | Target gene of miR-134-5p | |
| RFC1 | Target gene of miR-134-5p | |
| RGP1 | Target gene of miR-134-5p | |
| RGS14 | Target gene of miR-134-5p | |
| RGS4 | Target gene of miR-134-5p | |
| RGS6 | Target gene of miR-134-5p | |
| RHBG | Target gene of miR-134-5p | |
| RHCE | Target gene of miR-134-5p | |
| RIC8A | Target gene of miR-134-5p | |
| RIMS3 | Target gene of miR-134-5p | |
| RNASEH1 | Target gene of miR-134-5p | |
| RNF114 | Target gene of miR-134-5p | |
| RNF150 | Target gene of miR-134-5p | |
| RNF212 | Target gene of miR-134-5p | |
| RNFT2 | Target gene of miR-134-5p | |
| RNLS | Target gene of miR-134-5p | |
| RP2 | Target gene of miR-134-5p | |
| RPAIN | Target gene of miR-134-5p | |
| RPAP2 | Target gene of miR-134-5p | |
| RPAP3 | Target gene of miR-134-5p | |
| RPH3A | Target gene of miR-134-5p | |
| RPP30 | Target gene of miR-134-5p | |
| RPRD1A | Target gene of miR-134-5p | |
| RPRD1B | Target gene of miR-134-5p | |
| RPS6KC1 | Target gene of miR-134-5p | |
| RRAS2 | Target gene of miR-134-5p | |
| RSF1 | Target gene of miR-134-5p | |
| RSRC1 | Target gene of miR-134-5p | |
| RXFP2 | Target gene of miR-134-5p | |
| RYK | Target gene of miR-134-5p | |
| SAMD8 | Target gene of miR-134-5p | |
| SART3 | Target gene of miR-134-5p | |
| SAV1 | Target gene of miR-134-5p | |
| SBF2 | Target gene of miR-134-5p | |
| SBNO1 | Target gene of miR-134-5p | |
| SCN8A | Target gene of miR-134-5p | |
| SCOC | Target gene of miR-134-5p | |
| SDC4 | Target gene of miR-134-5p | |
| SDF2 | Target gene of miR-134-5p | |
| SDHC | Target gene of miR-134-5p | |
| SEC14L5 | Target gene of miR-134-5p | |
| SEC61A2 | Target gene of miR-134-5p | |
| SEH1L | Target gene of miR-134-5p | |
| SELP | Target gene of miR-134-5p | |
| SEMA7A | Target gene of miR-134-5p | |
| SENP2 | Target gene of miR-134-5p | |
| SENP7 | Target gene of miR-134-5p | |
| SERINC5 | Target gene of miR-134-5p | |
| SERPINH1 | Target gene of miR-134-5p | |
| SETD4 | Target gene of miR-134-5p | |
| SETD7 | Target gene of miR-134-5p | |
| SFT2D2 | Target gene of miR-134-5p | |
| SFXN1 | Target gene of miR-134-5p | |
| SGCZ | Target gene of miR-134-5p | |
| SH2D1A | Target gene of miR-134-5p | |
| SH3TC2 | Target gene of miR-134-5p | |
| SHH | Target gene of miR-134-5p | |
| SHISA2 | Target gene of miR-134-5p | |
| SHISA9 | Target gene of miR-134-5p | |
| SHMT2 | Target gene of miR-134-5p | |
| SIGLEC10 | Target gene of miR-134-5p | |
| SIRPB1 | Target gene of miR-134-5p | |
| SLAIN2 | Target gene of miR-134-5p | |
| SLAMF1 | Target gene of miR-134-5p | |
| SLC14A1 | Target gene of miR-134-5p | |
| SLC15A4 | Target gene of miR-134-5p | |
| SLC16A14 | Target gene of miR-134-5p | |
| SLC16A7 | Target gene of miR-134-5p | |
| SLC19A1 | Target gene of miR-134-5p | |
| SLC19A3 | Target gene of miR-134-5p | |
| SLC25A23 | Target gene of miR-134-5p | |
| SLC25A42 | Target gene of miR-134-5p | |
| SLC25A44 | Target gene of miR-134-5p | |
| SLC26A2 | Target gene of miR-134-5p | |
| SLC27A1 | Target gene of miR-134-5p | |
| SLC2A12 | Target gene of miR-134-5p | |
| SLC30A9 | Target gene of miR-134-5p | |
| SLC32A1 | Target gene of miR-134-5p | |
| SLC36A1 | Target gene of miR-134-5p | |
| SLC38A1 | Target gene of miR-134-5p | |
| SLC39A14 | Target gene of miR-134-5p | |
| SLC39A9 | Target gene of miR-134-5p | |
| SLC45A4 | Target gene of miR-134-5p | |
| SLC4A7 | Target gene of miR-134-5p | |
| SLC4A8 | Target gene of miR-134-5p | |
| SLC6A6 | Target gene of miR-134-5p | |
| SLC6A7 | Target gene of miR-134-5p | |
| SLC7A1 | Target gene of miR-134-5p | |
| SLC7A11 | Target gene of miR-134-5p | |
| SLC7A14 | Target gene of miR-134-5p | |
| SLC7A8 | Target gene of miR-134-5p | |
| SLC9A5 | Target gene of miR-134-5p | |
| SLIT1 | Target gene of miR-134-5p | |
| SLMAP | Target gene of miR-134-5p | |
| SMAD2 | Target gene of miR-134-5p | |
| SMAGP | Target gene of miR-134-5p | |
| SMAP2 | Target gene of miR-134-5p | |
| SMARCAD1 | Target gene of miR-134-5p | |
| SMARCE1 | Target gene of miR-134-5p | |
| SMU1 | Target gene of miR-134-5p | |
| SNAPC5 | Target gene of miR-134-5p | |
| SNCG | Target gene of miR-134-5p | |
| SNTB1 | Target gene of miR-134-5p | |
| SNX12 | Target gene of miR-134-5p | |
| SNX13 | Target gene of miR-134-5p | |
| SNX19 | Target gene of miR-134-5p | |
| SNX20 | Target gene of miR-134-5p | |
| SNX24 | Target gene of miR-134-5p | |
| SNX33 | Target gene of miR-134-5p | |
| SOD2 | Target gene of miR-134-5p | |
| SOHLH2 | Target gene of miR-134-5p | |
| SOX11 | Target gene of miR-134-5p | |
| SPARCL1 | Target gene of miR-134-5p | |
| SPATA17 | Target gene of miR-134-5p | |
| SPATA5 | Target gene of miR-134-5p | |
| SPCS3 | Target gene of miR-134-5p | |
| SPIB | Target gene of miR-134-5p | |
| SPON2 | Target gene of miR-134-5p | |
| SPPL3 | Target gene of miR-134-5p | |
| SPRR4 | Target gene of miR-134-5p | |
| SPRY4 | Target gene of miR-134-5p | |
| SPTLC1 | Target gene of miR-134-5p | |
| SPTSSB | Target gene of miR-134-5p | |
| SRGAP3 | Target gene of miR-134-5p | |
| SRP19 | Target gene of miR-134-5p | |
| SRP68 | Target gene of miR-134-5p | |
| SRPK2 | Target gene of miR-134-5p | |
| SRXN1 | Target gene of miR-134-5p | |
| SS18L1 | Target gene of miR-134-5p | |
| ST3GAL2 | Target gene of miR-134-5p | |
| ST6GAL2 | Target gene of miR-134-5p | |
| ST7L | Target gene of miR-134-5p | |
| ST8SIA5 | Target gene of miR-134-5p | |
| STARD8 | Target gene of miR-134-5p | |
| STK17B | Target gene of miR-134-5p | |
| STOML1 | Target gene of miR-134-5p | |
| STOX2 | Target gene of miR-134-5p | |
| STX11 | Target gene of miR-134-5p | |
| STX12 | Target gene of miR-134-5p | |
| STX17 | Target gene of miR-134-5p | |
| STX1B | Target gene of miR-134-5p | |
| SULT1B1 | Target gene of miR-134-5p | |
| SULT1C4 | Target gene of miR-134-5p | |
| SULT2A1 | Target gene of miR-134-5p | |
| SURF4 | Target gene of miR-134-5p | |
| SUSD3 | Target gene of miR-134-5p | |
| SV2C | Target gene of miR-134-5p | |
| SYAP1 | Target gene of miR-134-5p | |
| SYNGR2 | Target gene of miR-134-5p | |
| SYNJ2 | Target gene of miR-134-5p | |
| SZT2 | Target gene of miR-134-5p | |
| TAS2R4 | Target gene of miR-134-5p | |
| TBC1D23 | Target gene of miR-134-5p | |
| TBL1XR1 | Target gene of miR-134-5p | |
| TCERG1 | Target gene of miR-134-5p | |
| TCF25 | Target gene of miR-134-5p | |
| TCTN3 | Target gene of miR-134-5p | |
| TDRD6 | Target gene of miR-134-5p | |
| TEAD2 | Target gene of miR-134-5p | |
| TEF | Target gene of miR-134-5p | |
| TEK | Target gene of miR-134-5p | |
| TFAP2A | Target gene of miR-134-5p | |
| TFB2M | Target gene of miR-134-5p | |
| TGFB3 | Target gene of miR-134-5p | |
| TGFBRAP1 | Target gene of miR-134-5p | |
| TGIF1 | Target gene of miR-134-5p | |
| THAP6 | Target gene of miR-134-5p | |
| THUMPD3 | Target gene of miR-134-5p | |
| TIFA | Target gene of miR-134-5p | |
| TIGD6 | Target gene of miR-134-5p | |
| TIMM23 | Target gene of miR-134-5p | |
| TIPIN | Target gene of miR-134-5p | |
| TLR4 | Target gene of miR-134-5p | |
| TM2D2 | Target gene of miR-134-5p | |
| TM4SF4 | Target gene of miR-134-5p | |
| TMC5 | Target gene of miR-134-5p | |
| TMCO1 | Target gene of miR-134-5p | |
| TMCO5A | Target gene of miR-134-5p | |
| TMED10 | Target gene of miR-134-5p | |
| TMED3 | Target gene of miR-134-5p | |
| TMED4 | Target gene of miR-134-5p | |
| TMED5 | Target gene of miR-134-5p | |
| TMEM106B | Target gene of miR-134-5p | |
| TMEM132C | Target gene of miR-134-5p | |
| TMEM143 | Target gene of miR-134-5p | |
| TMEM170B | Target gene of miR-134-5p | |
| TMEM184A | Target gene of miR-134-5p | |
| TMEM196 | Target gene of miR-134-5p | |
| TMEM203 | Target gene of miR-134-5p | |
| TMEM217 | Target gene of miR-134-5p | |
| TMEM229B | Target gene of miR-134-5p | |
| TMEM237 | Target gene of miR-134-5p | |
| TMEM33 | Target gene of miR-134-5p | |
| TMEM65 | Target gene of miR-134-5p | |
| TMEM87B | Target gene of miR-134-5p | |
| TMEM92 | Target gene of miR-134-5p | |
| TMEM97 | Target gene of miR-134-5p | |
| TMEM98 | Target gene of miR-134-5p | |
| TMX4 | Target gene of miR-134-5p | |
| TNFRSF25 | Target gene of miR-134-5p | |
| TNFSF18 | Target gene of miR-134-5p | |
| TNIP2 | Target gene of miR-134-5p | |
| TOB2 | Target gene of miR-134-5p | |
| TOMM20 | Target gene of miR-134-5p | |
| TOP2A | Target gene of miR-134-5p | |
| TOR1A | Target gene of miR-134-5p | |
| TOR1B | Target gene of miR-134-5p | |
| TOX | Target gene of miR-134-5p | |
| TPD52L1 | Target gene of miR-134-5p | |
| TPD52L2 | Target gene of miR-134-5p | |
| TPRG1 | Target gene of miR-134-5p | |
| TRABD | Target gene of miR-134-5p | |
| TRAF3 | Target gene of miR-134-5p | |
| TRAPPC3 | Target gene of miR-134-5p | |
| TRAT1 | Target gene of miR-134-5p | |
| TRERF1 | Target gene of miR-134-5p | |
| TRIM37 | Target gene of miR-134-5p | |
| TRIM44 | Target gene of miR-134-5p | |
| TRIM72 | Target gene of miR-134-5p | |
| TRIM9 | Target gene of miR-134-5p | |
| TRMT12 | Target gene of miR-134-5p | |
| TRPC4 | Target gene of miR-134-5p | |
| TRPM7 | Target gene of miR-134-5p | |
| TRPS1 | Target gene of miR-134-5p | |
| TSKU | Target gene of miR-134-5p | |
| TSLP | Target gene of miR-134-5p | |
| TSN | Target gene of miR-134-5p | |
| TSPAN2 | Target gene of miR-134-5p | |
| TSPAN6 | Target gene of miR-134-5p | |
| TTC26 | Target gene of miR-134-5p | |
| TUB | Target gene of miR-134-5p | |
| TUBGCP2 | Target gene of miR-134-5p | |
| TUBGCP3 | Target gene of miR-134-5p | |
| TUSC3 | Target gene of miR-134-5p | |
| TXLNG | Target gene of miR-134-5p | |
| TXNDC12 | Target gene of miR-134-5p | |
| TYSND1 | Target gene of miR-134-5p | |
| U2SURP | Target gene of miR-134-5p | |
| UBE2H | Target gene of miR-134-5p | |
| UBE2J1 | Target gene of miR-134-5p | |
| UBE2K | Target gene of miR-134-5p | |
| UBE2L6 | Target gene of miR-134-5p | |
| UBE2W | Target gene of miR-134-5p | |
| UBE3A | Target gene of miR-134-5p | |
| UBFD1 | Target gene of miR-134-5p | |
| UBXN7 | Target gene of miR-134-5p | |
| ULK1 | Target gene of miR-134-5p | |
| UMPS | Target gene of miR-134-5p | |
| UNC5D | Target gene of miR-134-5p | |
| UNG | Target gene of miR-134-5p | |
| UPF3A | Target gene of miR-134-5p | |
| URM1 | Target gene of miR-134-5p | |
| USP15 | Target gene of miR-134-5p | |
| USP20 | Target gene of miR-134-5p | |
| USP36 | Target gene of miR-134-5p | |
| USP40 | Target gene of miR-134-5p | |
| USP42 | Target gene of miR-134-5p | |
| USP8 | Target gene of miR-134-5p | |
| UTP23 | Target gene of miR-134-5p | |
| UTP6 | Target gene of miR-134-5p | |
| VAMP3 | Target gene of miR-134-5p | |
| VAV2 | Target gene of miR-134-5p | |
| VCPIP1 | Target gene of miR-134-5p | |
| VGLL4 | Target gene of miR-134-5p | |
| VOPP1 | Target gene of miR-134-5p | |
| VSTM4 | Target gene of miR-134-5p | |
| WDR41 | Target gene of miR-134-5p | |
| WDR73 | Target gene of miR-134-5p | |
| WWC2 | Target gene of miR-134-5p | |
| WWP1 | Target gene of miR-134-5p | |
| YARS2 | Target gene of miR-134-5p | |
| YPEL3 | Target gene of miR-134-5p | |
| YTHDF3 | Target gene of miR-134-5p | |
| ZBTB16 | Target gene of miR-134-5p | |
| ZBTB20 | Target gene of miR-134-5p | |
| ZBTB33 | Target gene of miR-134-5p | |
| ZBTB7C | Target gene of miR-134-5p | |
| ZFP1 | Target gene of miR-134-5p | |
| ZFP3 | Target gene of miR-134-5p | |
| ZFP90 | Target gene of miR-134-5p | |
| ZHX1 | Target gene of miR-134-5p | |
| ZMAT3 | Target gene of miR-134-5p | |
| ZMAT5 | Target gene of miR-134-5p | |
| ZMYM1 | Target gene of miR-134-5p | |
| ZMYND11 | Target gene of miR-134-5p | |
| ZNF131 | Target gene of miR-134-5p | |
| ZNF19 | Target gene of miR-134-5p | |
| ZNF211 | Target gene of miR-134-5p | |
| ZNF24 | Target gene of miR-134-5p | |
| ZNF248 | Target gene of miR-134-5p | |
| ZNF26 | Target gene of miR-134-5p | |
| ZNF275 | Target gene of miR-134-5p | |
| ZNF276 | Target gene of miR-134-5p | |
| ZNF28 | Target gene of miR-134-5p | |
| ZNF281 | Target gene of miR-134-5p | |
| ZNF286A | Target gene of miR-134-5p | |
| ZNF292 | Target gene of miR-134-5p | |
| ZNF330 | Target gene of miR-134-5p | |
| ZNF347 | Target gene of miR-134-5p | |
| ZNF35 | Target gene of miR-134-5p | |
| ZNF382 | Target gene of miR-134-5p | |
| ZNF383 | Target gene of miR-134-5p | |
| ZNF415 | Target gene of miR-134-5p | |
| ZNF420 | Target gene of miR-134-5p | |
| ZNF431 | Target gene of miR-134-5p | |
| ZNF468 | Target gene of miR-134-5p | |
| ZNF491 | Target gene of miR-134-5p | |
| ZNF548 | Target gene of miR-134-5p | |
| ZNF555 | Target gene of miR-134-5p | |
| ZNF570 | Target gene of miR-134-5p | |
| ZNF578 | Target gene of miR-134-5p | |
| ZNF583 | Target gene of miR-134-5p | |
| ZNF607 | Target gene of miR-134-5p | |
| ZNF664 | Target gene of miR-134-5p | |
| ZNF669 | Target gene of miR-134-5p | |
| ZNF695 | Target gene of miR-134-5p | |
| ZNF706 | Target gene of miR-134-5p | |
| ZNF736 | Target gene of miR-134-5p | |
| ZNF737 | Target gene of miR-134-5p | |
| ZNF746 | Target gene of miR-134-5p | |
| ZNF831 | Target gene of miR-134-5p | |
| ZNRF3 | Target gene of miR-134-5p | |
| ZSCAN29 | Target gene of miR-134-5p | |
| ZW10 | Target gene of miR-134-5p | |
| A1CF | Target gene of miR-134-5p | |
| AAGAB | Target gene of miR-134-5p | |
| ABAT | Target gene of miR-134-5p | |
| ABCA2 | Target gene of miR-134-5p | |
| ABCG4 | Target gene of miR-134-5p | |
| ABHD2 | Target gene of miR-134-5p | |
| ABI2 | Target gene of miR-134-5p | |
| ABITRAM | Target gene of miR-134-5p | |
| ABO | Target gene of miR-134-5p | |
| ABT1 | Target gene of miR-134-5p | |
| ACE2 | Target gene of miR-134-5p | |
| ACKR1 | Target gene of miR-134-5p | |
| ACP3 | Target gene of miR-134-5p | |
| ACTG2 | Target gene of miR-134-5p | |
| ACTN1 | Target gene of miR-134-5p | |
| ACTR8 | Target gene of miR-134-5p | |
| ADA2 | Target gene of miR-134-5p | |
| ADAM11 | Target gene of miR-134-5p | |
| ADAM19 | Target gene of miR-134-5p | |
| ADAM23 | Target gene of miR-134-5p | |
| ADAT3 | Target gene of miR-134-5p | |
| ADCY1 | Target gene of miR-134-5p | |
| ADCYAP1 | Target gene of miR-134-5p | |
| ADCYAP1R1 | Target gene of miR-134-5p | |
| ADD3 | Target gene of miR-134-5p | |
| ADGRA1 | Target gene of miR-134-5p | |
| ADGRB1 | Target gene of miR-134-5p | |
| ADGRD1 | Target gene of miR-134-5p | |
| ADGRG6 | Target gene of miR-134-5p | |
| ADGRL2 | Target gene of miR-134-5p | |
| ADGRL3 | Target gene of miR-134-5p | |
| AFF1 | Target gene of miR-134-5p | |
| AFF3 | Target gene of miR-134-5p | |
| AGFG1 | Target gene of miR-134-5p | |
| AGFG2 | Target gene of miR-134-5p | |
| AGO1 | Target gene of miR-134-5p | |
| AGPAT1 | Target gene of miR-134-5p | |
| AGPAT3 | Target gene of miR-134-5p | |
| AHI1 | Target gene of miR-134-5p | |
| AIF1L | Target gene of miR-134-5p | |
| AKAP4 | Target gene of miR-134-5p | |
| AKAP6 | Target gene of miR-134-5p | |
| AKR1C2 | Target gene of miR-134-5p | |
| AKR7A2 | Target gene of miR-134-5p | |
| AKT3 | Target gene of miR-134-5p | |
| ALDH1L2 | Target gene of miR-134-5p | |
| ALDH2 | Target gene of miR-134-5p | |
| ALDH3A2 | Target gene of miR-134-5p | |
| ALDH4A1 | Target gene of miR-134-5p | |
| ALG11 | Target gene of miR-134-5p | |
| ALG13 | Target gene of miR-134-5p | |
| ALG9 | Target gene of miR-134-5p | |
| ALPL | Target gene of miR-134-5p | |
| ALS2 | Target gene of miR-134-5p | |
| ALS2CL | Target gene of miR-134-5p | |
| AMER2 | Target gene of miR-134-5p | |
| AMIGO1 | Target gene of miR-134-5p | |
| ANAPC15 | Target gene of miR-134-5p | |
| ANGPTL4 | Target gene of miR-134-5p | |
| ANKMY1 | Target gene of miR-134-5p | |
| ANKRD12 | Target gene of miR-134-5p | |
| ANKRD13B | Target gene of miR-134-5p | |
| ANKRD29 | Target gene of miR-134-5p | |
| ANKRD30B | Target gene of miR-134-5p | |
| ANKRD6 | Target gene of miR-134-5p | |
| ANKS1A | Target gene of miR-134-5p | |
| ANO3 | Target gene of miR-134-5p | |
| ANP32B | Target gene of miR-134-5p | |
| ANPEP | Target gene of miR-134-5p | |
| ANTXR2 | Target gene of miR-134-5p | |
| AOPEP | Target gene of miR-134-5p | |
| AP1S2 | Target gene of miR-134-5p | |
| AP3M1 | Target gene of miR-134-5p | |
| AP3M2 | Target gene of miR-134-5p | |
| AP4E1 | Target gene of miR-134-5p | |
| AP5M1 | Target gene of miR-134-5p | |
| APAF1 | Target gene of miR-134-5p | |
| APBA1 | Target gene of miR-134-5p | |
| APCDD1 | Target gene of miR-134-5p | |
| APOL2 | Target gene of miR-134-5p | |
| APOL4 | Target gene of miR-134-5p | |
| APOOL | Target gene of miR-134-5p | |
| AQP7 | Target gene of miR-134-5p | |
| AREL1 | Target gene of miR-134-5p | |
| ARF3 | Target gene of miR-134-5p | |
| ARF6 | Target gene of miR-134-5p | |
| ARFIP1 | Target gene of miR-134-5p | |
| ARHGAP36 | Target gene of miR-134-5p | |
| ARHGAP44 | Target gene of miR-134-5p | |
| ARHGAP8 | Target gene of miR-134-5p | |
| ARHGEF26 | Target gene of miR-134-5p | |
| ARHGEF40 | Target gene of miR-134-5p | |
| ARHGEF6 | Target gene of miR-134-5p | |
| ARHGEF7 | Target gene of miR-134-5p | |
| ARHGEF9 | Target gene of miR-134-5p | |
| ARL4A | Target gene of miR-134-5p | |
| ARMC5 | Target gene of miR-134-5p | |
| ARMC9 | Target gene of miR-134-5p | |
| ARMH3 | Target gene of miR-134-5p | |
| ARPIN | Target gene of miR-134-5p | |
| ARRB1 | Target gene of miR-134-5p | |
| ARSB | Target gene of miR-134-5p | |
| ASB13 | Target gene of miR-134-5p | |
| ASB18 | Target gene of miR-134-5p | |
| ASIC1 | Target gene of miR-134-5p | |
| ASIC2 | Target gene of miR-134-5p | |
| ASPHD2 | Target gene of miR-134-5p | |
| ASXL2 | Target gene of miR-134-5p | |
| ASXL3 | Target gene of miR-134-5p | |
| ATF7IP2 | Target gene of miR-134-5p | |
| ATG5 | Target gene of miR-134-5p | |
| ATL2 | Target gene of miR-134-5p | |
| ATM | Target gene of miR-134-5p | |
| ATP10B | Target gene of miR-134-5p | |
| ATP1B3 | Target gene of miR-134-5p | |
| ATP1B4 | Target gene of miR-134-5p | |
| ATP5MF-PTCD1 | Target gene of miR-134-5p | |
| ATP5PB | Target gene of miR-134-5p | |
| ATXN7 | Target gene of miR-134-5p | |
| ATXN7L3B | Target gene of miR-134-5p | |
| AUNIP | Target gene of miR-134-5p | |
| AVEN | Target gene of miR-134-5p | |
| AVPR2 | Target gene of miR-134-5p | |
| AWAT2 | Target gene of miR-134-5p | |
| AZIN2 | Target gene of miR-134-5p | |
| B3GALT5 | Target gene of miR-134-5p | |
| B4GALT1 | Target gene of miR-134-5p | |
| B4GAT1 | Target gene of miR-134-5p | |
| B9D1 | Target gene of miR-134-5p | |
| B9D2 | Target gene of miR-134-5p | |
| BACE1 | Target gene of miR-134-5p | |
| BACH1 | Target gene of miR-134-5p | |
| BACH2 | Target gene of miR-134-5p | |
| BAIAP2 | Target gene of miR-134-5p | |
| BAIAP3 | Target gene of miR-134-5p | |
| BANF1 | Target gene of miR-134-5p | |
| BARD1 | Target gene of miR-134-5p | |
| BAX | Target gene of miR-134-5p | |
| BAZ2A | Target gene of miR-134-5p | |
| BCAS1 | Target gene of miR-134-5p | |
| BCAS3 | Target gene of miR-134-5p | |
| BCL2L11 | Target gene of miR-134-5p | |
| BCL6B | Target gene of miR-134-5p | |
| BDNF | Target gene of miR-134-5p | |
| BEND4 | Target gene of miR-134-5p | |
| BICRAL | Target gene of miR-134-5p | |
| BLM | Target gene of miR-134-5p | |
| BLMH | Target gene of miR-134-5p | |
| BLOC1S2 | Target gene of miR-134-5p | |
| BLOC1S3 | Target gene of miR-134-5p | |
| BMP10 | Target gene of miR-134-5p | |
| BMT2 | Target gene of miR-134-5p | |
| BNIP3L | Target gene of miR-134-5p | |
| BOLL | Target gene of miR-134-5p | |
| BORCS5 | Target gene of miR-134-5p | |
| BPIFA2 | Target gene of miR-134-5p | |
| BRAP | Target gene of miR-134-5p | |
| BRD7 | Target gene of miR-134-5p | |
| BRI3BP | Target gene of miR-134-5p | |
| BROX | Target gene of miR-134-5p | |
| BRPF1 | Target gene of miR-134-5p | |
| BRWD3 | Target gene of miR-134-5p | |
| BTBD9 | Target gene of miR-134-5p | |
| BTG1 | Target gene of miR-134-5p | |
| BTN3A3 | Target gene of miR-134-5p | |
| BTNL3 | Target gene of miR-134-5p | |
| BVES | Target gene of miR-134-5p | |
| C10orf143 | Target gene of miR-134-5p | |
| C12orf76 | Target gene of miR-134-5p | |
| C16orf72 | Target gene of miR-134-5p | |
| C16orf74 | Target gene of miR-134-5p | |
| C16orf82 | Target gene of miR-134-5p | |
| C18orf54 | Target gene of miR-134-5p | |
| C1orf112 | Target gene of miR-134-5p | |
| C1QC | Target gene of miR-134-5p | |
| C1QTNF6 | Target gene of miR-134-5p | |
| C20orf27 | Target gene of miR-134-5p | |
| C22orf39 | Target gene of miR-134-5p | |
| C2CD2 | Target gene of miR-134-5p | |
| C2orf50 | Target gene of miR-134-5p | |
| C3orf22 | Target gene of miR-134-5p | |
| C3orf70 | Target gene of miR-134-5p | |
| C3orf80 | Target gene of miR-134-5p | |
| C4orf33 | Target gene of miR-134-5p | |
| C5AR2 | Target gene of miR-134-5p | |
| C5orf22 | Target gene of miR-134-5p | |
| C5orf46 | Target gene of miR-134-5p | |
| C5orf63 | Target gene of miR-134-5p | |
| C6orf120 | Target gene of miR-134-5p | |
| C6orf62 | Target gene of miR-134-5p | |
| C8orf82 | Target gene of miR-134-5p | |
| CA7 | Target gene of miR-134-5p | |
| CAB39 | Target gene of miR-134-5p | |
| CACNA1C | Target gene of miR-134-5p | |
| CACNA1D | Target gene of miR-134-5p | |
| CACNA1I | Target gene of miR-134-5p | |
| CACNG1 | Target gene of miR-134-5p | |
| CACNG4 | Target gene of miR-134-5p | |
| CALCA | Target gene of miR-134-5p | |
| CALCOCO1 | Target gene of miR-134-5p | |
| CALCOCO2 | Target gene of miR-134-5p | |
| CALCRL | Target gene of miR-134-5p | |
| CALHM5 | Target gene of miR-134-5p | |
| CALM3 | Target gene of miR-134-5p | |
| CAMK1D | Target gene of miR-134-5p | |
| CAMK2A | Target gene of miR-134-5p | |
| CAMK2G | Target gene of miR-134-5p | |
| CAMK4 | Target gene of miR-134-5p | |
| CAMKK2 | Target gene of miR-134-5p | |
| CAPN12 | Target gene of miR-134-5p | |
| CAPN13 | Target gene of miR-134-5p | |
| CAPN7 | Target gene of miR-134-5p | |
| CAPZA1 | Target gene of miR-134-5p | |
| CAPZB | Target gene of miR-134-5p | |
| CARHSP1 | Target gene of miR-134-5p | |
| CASC3 | Target gene of miR-134-5p | |
| CASP3 | Target gene of miR-134-5p | |
| CASQ2 | Target gene of miR-134-5p | |
| CASR | Target gene of miR-134-5p | |
| CAVIN4 | Target gene of miR-134-5p | |
| CBFA2T2 | Target gene of miR-134-5p | |
| CBL | Target gene of miR-134-5p | |
| CBR4 | Target gene of miR-134-5p | |
| CBX5 | Target gene of miR-134-5p | |
| CC2D2B | Target gene of miR-134-5p | |
| CCDC102B | Target gene of miR-134-5p | |
| CCDC115 | Target gene of miR-134-5p | |
| CCDC116 | Target gene of miR-134-5p | |
| CCDC117 | Target gene of miR-134-5p | |
| CCDC144A | Target gene of miR-134-5p | |
| CCDC152 | Target gene of miR-134-5p | |
| CCDC170 | Target gene of miR-134-5p | |
| CCDC174 | Target gene of miR-134-5p | |
| CCDC198 | Target gene of miR-134-5p | |
| CCDC3 | Target gene of miR-134-5p | |
| CCDC32 | Target gene of miR-134-5p | |
| CCDC62 | Target gene of miR-134-5p | |
| CCDC71L | Target gene of miR-134-5p | |
| CCDC9B | Target gene of miR-134-5p | |
| CCL16 | Target gene of miR-134-5p | |
| CCNJL | Target gene of miR-134-5p | |
| CCSAP | Target gene of miR-134-5p | |
| CD200R1 | Target gene of miR-134-5p | |
| CD37 | Target gene of miR-134-5p | |
| CD4 | Target gene of miR-134-5p | |
| CD47 | Target gene of miR-134-5p | |
| CD53 | Target gene of miR-134-5p | |
| CD59 | Target gene of miR-134-5p | |
| CD74 | Target gene of miR-134-5p | |
| CD83 | Target gene of miR-134-5p | |
| CD8B2 | Target gene of miR-134-5p | |
| CD99 | Target gene of miR-134-5p | |
| CD99L2 | Target gene of miR-134-5p | |
| CDC14A | Target gene of miR-134-5p | |
| CDC14B | Target gene of miR-134-5p | |
| CDC27 | Target gene of miR-134-5p | |
| CDC42SE2 | Target gene of miR-134-5p | |
| CDC6 | Target gene of miR-134-5p | |
| CDCA8 | Target gene of miR-134-5p | |
| CDH11 | Target gene of miR-134-5p | |
| CDH17 | Target gene of miR-134-5p | |
| CDH20 | Target gene of miR-134-5p | |
| CDH7 | Target gene of miR-134-5p | |
| CDH8 | Target gene of miR-134-5p | |
| CDHR3 | Target gene of miR-134-5p | |
| CDK14 | Target gene of miR-134-5p | |
| CDK15 | Target gene of miR-134-5p | |
| CDK19 | Target gene of miR-134-5p | |
| CDK6 | Target gene of miR-134-5p | |
| CDKN1A | Target gene of miR-134-5p | |
| CDKN2AIP | Target gene of miR-134-5p | |
| CDON | Target gene of miR-134-5p | |
| CDS2 | Target gene of miR-134-5p | |
| CELF1 | Target gene of miR-134-5p | |
| CELF2 | Target gene of miR-134-5p | |
| CELF5 | Target gene of miR-134-5p | |
| CEMP1 | Target gene of miR-134-5p | |
| CENPC | Target gene of miR-134-5p | |
| CENPE | Target gene of miR-134-5p | |
| CENPO | Target gene of miR-134-5p | |
| CEP164 | Target gene of miR-134-5p | |
| CEP43 | Target gene of miR-134-5p | |
| CEP57L1 | Target gene of miR-134-5p | |
| CEP63 | Target gene of miR-134-5p | |
| CERKL | Target gene of miR-134-5p | |
| CERS2 | Target gene of miR-134-5p | |
| CES4A | Target gene of miR-134-5p | |
| CFAP157 | Target gene of miR-134-5p | |
| CFAP57 | Target gene of miR-134-5p | |
| CFAP77 | Target gene of miR-134-5p | |
| CFAP92 | Target gene of miR-134-5p | |
| CFAP97D1 | Target gene of miR-134-5p | |
| CFL1 | Target gene of miR-134-5p | |
| CFLAR | Target gene of miR-134-5p | |
| CHDH | Target gene of miR-134-5p | |
| CHIC1 | Target gene of miR-134-5p | |
| CHMP1A | Target gene of miR-134-5p | |
| CHMP3 | Target gene of miR-134-5p | |
| CHMP4B | Target gene of miR-134-5p | |
| CHODL | Target gene of miR-134-5p | |
| CHRFAM7A | Target gene of miR-134-5p | |
| CHRM4 | Target gene of miR-134-5p | |
| CHRNA7 | Target gene of miR-134-5p | |
| CHST15 | Target gene of miR-134-5p | |
| CHST6 | Target gene of miR-134-5p | |
| CIAO2A | Target gene of miR-134-5p | |
| CIRBP | Target gene of miR-134-5p | |
| CKAP4 | Target gene of miR-134-5p | |
| CLCN6 | Target gene of miR-134-5p | |
| CLDN18 | Target gene of miR-134-5p | |
| CLEC16A | Target gene of miR-134-5p | |
| CLEC4M | Target gene of miR-134-5p | |
| CLIC5 | Target gene of miR-134-5p | |
| CLK3 | Target gene of miR-134-5p | |
| CLNS1A | Target gene of miR-134-5p | |
| CLSPN | Target gene of miR-134-5p | |
| CLSTN2 | Target gene of miR-134-5p | |
| CLUAP1 | Target gene of miR-134-5p | |
| CLVS1 | Target gene of miR-134-5p | |
| CLYBL | Target gene of miR-134-5p | |
| CMA1 | Target gene of miR-134-5p | |
| CMAS | Target gene of miR-134-5p | |
| CMC2 | Target gene of miR-134-5p | |
| CMIP | Target gene of miR-134-5p | |
| CMSS1 | Target gene of miR-134-5p | |
| CNIH1 | Target gene of miR-134-5p | |
| CNNM2 | Target gene of miR-134-5p | |
| CNOT8 | Target gene of miR-134-5p | |
| CNPPD1 | Target gene of miR-134-5p | |
| CNR1 | Target gene of miR-134-5p | |
| CNRIP1 | Target gene of miR-134-5p | |
| CNTLN | Target gene of miR-134-5p | |
| CNTN2 | Target gene of miR-134-5p | |
| CNTN5 | Target gene of miR-134-5p | |
| CNTNAP5 | Target gene of miR-134-5p | |
| COA1 | Target gene of miR-134-5p | |
| COG7 | Target gene of miR-134-5p | |
| COL20A1 | Target gene of miR-134-5p | |
| COL4A6 | Target gene of miR-134-5p | |
| COL6A3 | Target gene of miR-134-5p | |
| COL9A2 | Target gene of miR-134-5p | |
| COLGALT2 | Target gene of miR-134-5p | |
| COMMD3-BMI1 | Target gene of miR-134-5p | |
| COMMD7 | Target gene of miR-134-5p | |
| COPRS | Target gene of miR-134-5p | |
| CORO1C | Target gene of miR-134-5p | |
| COX6B2 | Target gene of miR-134-5p | |
| COX8A | Target gene of miR-134-5p | |
| CPEB3 | Target gene of miR-134-5p | |
| CPEB4 | Target gene of miR-134-5p | |
| CPLX4 | Target gene of miR-134-5p | |
| CPNE4 | Target gene of miR-134-5p | |
| CPPED1 | Target gene of miR-134-5p | |
| CPSF2 | Target gene of miR-134-5p | |
| CRCP | Target gene of miR-134-5p | |
| CREB1 | Target gene of miR-134-5p | |
| CREB5 | Target gene of miR-134-5p | |
| CREBZF | Target gene of miR-134-5p | |
| CRELD2 | Target gene of miR-134-5p | |
| CRHR2 | Target gene of miR-134-5p | |
| CRLS1 | Target gene of miR-134-5p | |
| CRMP1 | Target gene of miR-134-5p | |
| CRTAP | Target gene of miR-134-5p | |
| CRYGN | Target gene of miR-134-5p | |
| CSGALNACT2 | Target gene of miR-134-5p | |
| CST3 | Target gene of miR-134-5p | |
| CTC1 | Target gene of miR-134-5p | |
| CTDSPL | Target gene of miR-134-5p | |
| CTIF | Target gene of miR-134-5p | |
| CTNS | Target gene of miR-134-5p | |
| CTRC | Target gene of miR-134-5p | |
| CTSC | Target gene of miR-134-5p | |
| CUX2 | Target gene of miR-134-5p | |
| CXXC4 | Target gene of miR-134-5p | |
| CYB561A3 | Target gene of miR-134-5p | |
| CYB5D1 | Target gene of miR-134-5p | |
| CYB5D2 | Target gene of miR-134-5p | |
| CYB5RL | Target gene of miR-134-5p | |
| CYBRD1 | Target gene of miR-134-5p | |
| CYFIP2 | Target gene of miR-134-5p | |
| CYLC2 | Target gene of miR-134-5p | |
| CYP11A1 | Target gene of miR-134-5p | |
| CYP1B1 | Target gene of miR-134-5p | |
| CYP20A1 | Target gene of miR-134-5p | |
| CYP26B1 | Target gene of miR-134-5p | |
| CYP4A22 | Target gene of miR-134-5p | |
| CYREN | Target gene of miR-134-5p | |
| CYRIB | Target gene of miR-134-5p | |
| DAAM2 | Target gene of miR-134-5p | |
| DACT2 | Target gene of miR-134-5p | |
| DAND5 | Target gene of miR-134-5p | |
| DAOA | Target gene of miR-134-5p | |
| DAZ1 | Target gene of miR-134-5p | |
| DAZ3 | Target gene of miR-134-5p | |
| DAZ4 | Target gene of miR-134-5p | |
| DAZL | Target gene of miR-134-5p | |
| DBNL | Target gene of miR-134-5p | |
| DCAF12 | Target gene of miR-134-5p | |
| DCAF12L2 | Target gene of miR-134-5p | |
| DCAF5 | Target gene of miR-134-5p | |
| DCC | Target gene of miR-134-5p | |
| DCDC2C | Target gene of miR-134-5p | |
| DCLK2 | Target gene of miR-134-5p | |
| DCN | Target gene of miR-134-5p | |
| DCP1A | Target gene of miR-134-5p | |
| DCP2 | Target gene of miR-134-5p | |
| DCPS | Target gene of miR-134-5p | |
| DCUN1D1 | Target gene of miR-134-5p | |
| DCUN1D3 | Target gene of miR-134-5p | |
| DDI2 | Target gene of miR-134-5p | |
| DDR2 | Target gene of miR-134-5p | |
| DEF8 | Target gene of miR-134-5p | |
| DEFB134 | Target gene of miR-134-5p | |
| DEK | Target gene of miR-134-5p | |
| DENND11 | Target gene of miR-134-5p | |
| DENND1A | Target gene of miR-134-5p | |
| DENND6A | Target gene of miR-134-5p | |
| DERL2 | Target gene of miR-134-5p | |
| DESI2 | Target gene of miR-134-5p | |
| DFFB | Target gene of miR-134-5p | |
| DGAT2 | Target gene of miR-134-5p | |
| DGKB | Target gene of miR-134-5p | |
| DHFR2 | Target gene of miR-134-5p | |
| DHRS13 | Target gene of miR-134-5p | |
| DIAPH2 | Target gene of miR-134-5p | |
| DIO2 | Target gene of miR-134-5p | |
| DIP2B | Target gene of miR-134-5p | |
| DIPK2B | Target gene of miR-134-5p | |
| DIRAS1 | Target gene of miR-134-5p | |
| DISP2 | Target gene of miR-134-5p | |
| DLG2 | Target gene of miR-134-5p | |
| DLK1 | Target gene of miR-134-5p | |
| DLX4 | Target gene of miR-134-5p | |
| DMAC2 | Target gene of miR-134-5p | |
| DMD | Target gene of miR-134-5p | |
| DNAAF11 | Target gene of miR-134-5p | |
| DNAJB5 | Target gene of miR-134-5p | |
| DNAJC15 | Target gene of miR-134-5p | |
| DNAJC18 | Target gene of miR-134-5p | |
| DNAL1 | Target gene of miR-134-5p | |
| DNALI1 | Target gene of miR-134-5p | |
| DNASE1L1 | Target gene of miR-134-5p | |
| DND1 | Target gene of miR-134-5p | |
| DOLPP1 | Target gene of miR-134-5p | |
| DPF2 | Target gene of miR-134-5p | |
| DPH6 | Target gene of miR-134-5p | |
| DPY19L3 | Target gene of miR-134-5p | |
| DRAXIN | Target gene of miR-134-5p | |
| DRD3 | Target gene of miR-134-5p | |
| DRP2 | Target gene of miR-134-5p | |
| DSC3 | Target gene of miR-134-5p | |
| DTD2 | Target gene of miR-134-5p | |
| DTHD1 | Target gene of miR-134-5p | |
| DTL | Target gene of miR-134-5p | |
| DTNA | Target gene of miR-134-5p | |
| DTX3 | Target gene of miR-134-5p | |
| DTX3L | Target gene of miR-134-5p | |
| DTX4 | Target gene of miR-134-5p | |
| DUS4L-BCAP29 | Target gene of miR-134-5p | |
| DUSP1 | Target gene of miR-134-5p | |
| DUSP13 | Target gene of miR-134-5p | |
| DUSP18 | Target gene of miR-134-5p | |
| DUSP28 | Target gene of miR-134-5p | |
| DYRK1A | Target gene of miR-134-5p | |
| DZANK1 | Target gene of miR-134-5p | |
| DZIP1 | Target gene of miR-134-5p | |
| EBF1 | Target gene of miR-134-5p | |
| ECI1 | Target gene of miR-134-5p | |
| EDA2R | Target gene of miR-134-5p | |
| EDAR | Target gene of miR-134-5p | |
| EDNRA | Target gene of miR-134-5p | |
| EEF1A1 | Target gene of miR-134-5p | |
| EEPD1 | Target gene of miR-134-5p | |
| EFCAB1 | Target gene of miR-134-5p | |
| EFCAB5 | Target gene of miR-134-5p | |
| EFCC1 | Target gene of miR-134-5p | |
| EFEMP2 | Target gene of miR-134-5p | |
| EFHD2 | Target gene of miR-134-5p | |
| EFNB1 | Target gene of miR-134-5p | |
| EFS | Target gene of miR-134-5p | |
| EGR2 | Target gene of miR-134-5p | |
| EHMT1 | Target gene of miR-134-5p | |
| EID2 | Target gene of miR-134-5p | |
| EIF2AK1 | Target gene of miR-134-5p | |
| EIF2B1 | Target gene of miR-134-5p | |
| EIF3H | Target gene of miR-134-5p | |
| EIF4E2 | Target gene of miR-134-5p | |
| EIF4EBP2 | Target gene of miR-134-5p | |
| EIF4G2 | Target gene of miR-134-5p | |
| EIF5 | Target gene of miR-134-5p | |
| EIF5A | Target gene of miR-134-5p | |
| EIF5A2 | Target gene of miR-134-5p | |
| EIPR1 | Target gene of miR-134-5p | |
| ELAPOR1 | Target gene of miR-134-5p | |
| ELFN2 | Target gene of miR-134-5p | |
| ELMO1 | Target gene of miR-134-5p | |
| ELMO2 | Target gene of miR-134-5p | |
| ELMOD1 | Target gene of miR-134-5p | |
| ELOA | Target gene of miR-134-5p | |
| ELOC | Target gene of miR-134-5p | |
| ELOF1 | Target gene of miR-134-5p | |
| ELOVL2 | Target gene of miR-134-5p | |
| EMD | Target gene of miR-134-5p | |
| EME2 | Target gene of miR-134-5p | |
| EML5 | Target gene of miR-134-5p | |
| EML6 | Target gene of miR-134-5p | |
| EMP1 | Target gene of miR-134-5p | |
| ENDOD1 | Target gene of miR-134-5p | |
| ENOSF1 | Target gene of miR-134-5p | |
| ENOX2 | Target gene of miR-134-5p | |
| ENPP1 | Target gene of miR-134-5p | |
| ENPP4 | Target gene of miR-134-5p | |
| ENTPD3 | Target gene of miR-134-5p | |
| EPAS1 | Target gene of miR-134-5p | |
| EPB41L2 | Target gene of miR-134-5p | |
| EPC1 | Target gene of miR-134-5p | |
| EPHA4 | Target gene of miR-134-5p | |
| EPHA6 | Target gene of miR-134-5p | |
| EPHB2 | Target gene of miR-134-5p | |
| EPN1 | Target gene of miR-134-5p | |
| ERC1 | Target gene of miR-134-5p | |
| ERCC6L2 | Target gene of miR-134-5p | |
| EREG | Target gene of miR-134-5p | |
| ERG28 | Target gene of miR-134-5p | |
| ERMAP | Target gene of miR-134-5p | |
| ERO1A | Target gene of miR-134-5p | |
| ERVV-2 | Target gene of miR-134-5p | |
| ESM1 | Target gene of miR-134-5p | |
| ESR1 | Target gene of miR-134-5p | |
| ESR2 | Target gene of miR-134-5p | |
| ESRRB | Target gene of miR-134-5p | |
| ESYT2 | Target gene of miR-134-5p | |
| ETFBKMT | Target gene of miR-134-5p | |
| ETFRF1 | Target gene of miR-134-5p | |
| EVI5L | Target gene of miR-134-5p | |
| EVL | Target gene of miR-134-5p | |
| EXO1 | Target gene of miR-134-5p | |
| EXOC5 | Target gene of miR-134-5p | |
| EXOSC2 | Target gene of miR-134-5p | |
| EXOSC6 | Target gene of miR-134-5p | |
| EYA3 | Target gene of miR-134-5p | |
| F11R | Target gene of miR-134-5p | |
| F2RL2 | Target gene of miR-134-5p | |
| FABP3 | Target gene of miR-134-5p | |
| FAH | Target gene of miR-134-5p | |
| FAHD2A | Target gene of miR-134-5p | |
| FAM107B | Target gene of miR-134-5p | |
| FAM114A2 | Target gene of miR-134-5p | |
| FAM120B | Target gene of miR-134-5p | |
| FAM124A | Target gene of miR-134-5p | |
| FAM126A | Target gene of miR-134-5p | |
| FAM149B1 | Target gene of miR-134-5p | |
| FAM167A | Target gene of miR-134-5p | |
| FAM172A | Target gene of miR-134-5p | |
| FAM180A | Target gene of miR-134-5p | |
| FAM185A | Target gene of miR-134-5p | |
| FAM204A | Target gene of miR-134-5p | |
| FAM210A | Target gene of miR-134-5p | |
| FAM222A | Target gene of miR-134-5p | |
| FAM229B | Target gene of miR-134-5p | |
| FAM236C | Target gene of miR-134-5p | |
| FAM236D | Target gene of miR-134-5p | |
| FAM240A | Target gene of miR-134-5p | |
| FAM241A | Target gene of miR-134-5p | |
| FAM3A | Target gene of miR-134-5p | |
| FAM71F2 | Target gene of miR-134-5p | |
| FAM76A | Target gene of miR-134-5p | |
| FAM78B | Target gene of miR-134-5p | |
| FAM83C | Target gene of miR-134-5p | |
| FAM83F | Target gene of miR-134-5p | |
| FAT2 | Target gene of miR-134-5p | |
| FAXC | Target gene of miR-134-5p | |
| FBLN1 | Target gene of miR-134-5p | |
| FBLN2 | Target gene of miR-134-5p | |
| FBN1 | Target gene of miR-134-5p | |
| FBRSL1 | Target gene of miR-134-5p | |
| FBXL19 | Target gene of miR-134-5p | |
| FBXL2 | Target gene of miR-134-5p | |
| FBXL22 | Target gene of miR-134-5p | |
| FBXL5 | Target gene of miR-134-5p | |
| FBXL7 | Target gene of miR-134-5p | |
| FBXO31 | Target gene of miR-134-5p | |
| FBXO40 | Target gene of miR-134-5p | |
| FBXO41 | Target gene of miR-134-5p | |
| FBXO47 | Target gene of miR-134-5p | |
| FCMR | Target gene of miR-134-5p | |
| FERMT1 | Target gene of miR-134-5p | |
| FGF1 | Target gene of miR-134-5p | |
| FGF11 | Target gene of miR-134-5p | |
| FGF14 | Target gene of miR-134-5p | |
| FGF18 | Target gene of miR-134-5p | |
| FGF7 | Target gene of miR-134-5p | |
| FHAD1 | Target gene of miR-134-5p | |
| FHDC1 | Target gene of miR-134-5p | |
| FHIP1A | Target gene of miR-134-5p | |
| FHIP2A | Target gene of miR-134-5p | |
| FHIP2B | Target gene of miR-134-5p | |
| FIBCD1 | Target gene of miR-134-5p | |
| FICD | Target gene of miR-134-5p | |
| FILIP1L | Target gene of miR-134-5p | |
| FKBP5 | Target gene of miR-134-5p | |
| FLOT2 | Target gene of miR-134-5p | |
| FLRT3 | Target gene of miR-134-5p | |
| FLT4 | Target gene of miR-134-5p | |
| FLVCR2 | Target gene of miR-134-5p | |
| FMN2 | Target gene of miR-134-5p | |
| FMNL3 | Target gene of miR-134-5p | |
| FMOD | Target gene of miR-134-5p | |
| FOXA3 | Target gene of miR-134-5p | |
| FOXJ3 | Target gene of miR-134-5p | |
| FOXL2 | Target gene of miR-134-5p | |
| FOXO3 | Target gene of miR-134-5p | |
| FOXP1 | Target gene of miR-134-5p | |
| FOXP2 | Target gene of miR-134-5p | |
| FRA10AC1 | Target gene of miR-134-5p | |
| FRAT1 | Target gene of miR-134-5p | |
| FRK | Target gene of miR-134-5p | |
| FRMD1 | Target gene of miR-134-5p | |
| FRMD3 | Target gene of miR-134-5p | |
| FRMD7 | Target gene of miR-134-5p | |
| FRMPD2 | Target gene of miR-134-5p | |
| FRS2 | Target gene of miR-134-5p | |
| FSCN3 | Target gene of miR-134-5p | |
| FSTL1 | Target gene of miR-134-5p | |
| FSTL4 | Target gene of miR-134-5p | |
| FTCD | Target gene of miR-134-5p | |
| FTO | Target gene of miR-134-5p | |
| FTSJ1 | Target gene of miR-134-5p | |
| FUNDC2 | Target gene of miR-134-5p | |
| G6PC1 | Target gene of miR-134-5p | |
| GAB2 | Target gene of miR-134-5p | |
| GABRA2 | Target gene of miR-134-5p | |
| GABRA4 | Target gene of miR-134-5p | |
| GABRB2 | Target gene of miR-134-5p | |
| GADL1 | Target gene of miR-134-5p | |
| GALNT1 | Target gene of miR-134-5p | |
| GALNT16 | Target gene of miR-134-5p | |
| GALNT2 | Target gene of miR-134-5p | |
| GAPVD1 | Target gene of miR-134-5p | |
| GAS7 | Target gene of miR-134-5p | |
| GATAD2B | Target gene of miR-134-5p | |
| GATD1 | Target gene of miR-134-5p | |
| GATD3A | Target gene of miR-134-5p | |
| GCH1 | Target gene of miR-134-5p | |
| GCSAM | Target gene of miR-134-5p | |
| GCSAML | Target gene of miR-134-5p | |
| GEMIN7 | Target gene of miR-134-5p | |
| GFOD2 | Target gene of miR-134-5p | |
| GFRA4 | Target gene of miR-134-5p | |
| GGA2 | Target gene of miR-134-5p | |
| GID8 | Target gene of miR-134-5p | |
| GJB5 | Target gene of miR-134-5p | |
| GLP2R | Target gene of miR-134-5p | |
| GLRA2 | Target gene of miR-134-5p | |
| GLRX5 | Target gene of miR-134-5p | |
| GLUL | Target gene of miR-134-5p | |
| GNB4 | Target gene of miR-134-5p | |
| GNG4 | Target gene of miR-134-5p | |
| GOLGA3 | Target gene of miR-134-5p | |
| GOLGA6L9 | Target gene of miR-134-5p | |
| GOLGA8H | Target gene of miR-134-5p | |
| GOLGA8J | Target gene of miR-134-5p | |
| GOLGA8K | Target gene of miR-134-5p | |
| GOLGA8M | Target gene of miR-134-5p | |
| GOLGA8N | Target gene of miR-134-5p | |
| GOLGA8O | Target gene of miR-134-5p | |
| GOLGA8Q | Target gene of miR-134-5p | |
| GOLGA8R | Target gene of miR-134-5p | |
| GOLGA8T | Target gene of miR-134-5p | |
| GPALPP1 | Target gene of miR-134-5p | |
| GPAT4 | Target gene of miR-134-5p | |
| GPATCH11 | Target gene of miR-134-5p | |
| GPATCH2L | Target gene of miR-134-5p | |
| GPATCH8 | Target gene of miR-134-5p | |
| GPBP1L1 | Target gene of miR-134-5p | |
| GPC4 | Target gene of miR-134-5p | |
| GPKOW | Target gene of miR-134-5p | |
| GPN1 | Target gene of miR-134-5p | |
| GPN2 | Target gene of miR-134-5p | |
| GPR12 | Target gene of miR-134-5p | |
| GPR137 | Target gene of miR-134-5p | |
| GPR137C | Target gene of miR-134-5p | |
| GPR139 | Target gene of miR-134-5p | |
| GPR161 | Target gene of miR-134-5p | |
| GPR176 | Target gene of miR-134-5p | |
| GPR63 | Target gene of miR-134-5p | |
| GPRC5C | Target gene of miR-134-5p | |
| GPRIN2 | Target gene of miR-134-5p | |
| GPRIN3 | Target gene of miR-134-5p | |
| GREB1L | Target gene of miR-134-5p | |
| GRHL1 | Target gene of miR-134-5p | |
| GRIK1 | Target gene of miR-134-5p | |
| GRIK5 | Target gene of miR-134-5p | |
| GRIN2C | Target gene of miR-134-5p | |
| GRK5 | Target gene of miR-134-5p | |
| GRM4 | Target gene of miR-134-5p | |
| GSDMC | Target gene of miR-134-5p | |
| GSG1 | Target gene of miR-134-5p | |
| GSKIP | Target gene of miR-134-5p | |
| GSR | Target gene of miR-134-5p | |
| GSTT2B | Target gene of miR-134-5p | |
| GSX1 | Target gene of miR-134-5p | |
| GTF2A1 | Target gene of miR-134-5p | |
| GTF2H2C | Target gene of miR-134-5p | |
| GTF3C5 | Target gene of miR-134-5p | |
| GTPBP2 | Target gene of miR-134-5p | |
| GUCY1A1 | Target gene of miR-134-5p | |
| GVQW3 | Target gene of miR-134-5p | |
| GYG1 | Target gene of miR-134-5p | |
| GZF1 | Target gene of miR-134-5p | |
| H2AZ2 | Target gene of miR-134-5p | |
| H2BW2 | Target gene of miR-134-5p | |
| HAP1 | Target gene of miR-134-5p | |
| HCFC1 | Target gene of miR-134-5p | |
| HDAC11 | Target gene of miR-134-5p | |
| HDAC6 | Target gene of miR-134-5p | |
| HDAC9 | Target gene of miR-134-5p | |
| HDC | Target gene of miR-134-5p | |
| HDGFL3 | Target gene of miR-134-5p | |
| HDLBP | Target gene of miR-134-5p | |
| HECTD3 | Target gene of miR-134-5p | |
| HECW2 | Target gene of miR-134-5p | |
| HEG1 | Target gene of miR-134-5p | |
| HEMK1 | Target gene of miR-134-5p | |
| HES6 | Target gene of miR-134-5p | |
| HEXIM1 | Target gene of miR-134-5p | |
| HFE | Target gene of miR-134-5p | |
| HHIP | Target gene of miR-134-5p | |
| HIC1 | Target gene of miR-134-5p | |
| HIF1AN | Target gene of miR-134-5p | |
| HIF3A | Target gene of miR-134-5p | |
| HINFP | Target gene of miR-134-5p | |
| HIP1R | Target gene of miR-134-5p | |
| HIVEP2 | Target gene of miR-134-5p | |
| HIVEP3 | Target gene of miR-134-5p | |
| HMGN1 | Target gene of miR-134-5p | |
| HNF1A | Target gene of miR-134-5p | |
| HNF1B | Target gene of miR-134-5p | |
| HNRNPA2B1 | Target gene of miR-134-5p | |
| HNRNPC | Target gene of miR-134-5p | |
| HNRNPDL | Target gene of miR-134-5p | |
| HNRNPH1 | Target gene of miR-134-5p | |
| HNRNPU | Target gene of miR-134-5p | |
| HOOK3 | Target gene of miR-134-5p | |
| HOXB8 | Target gene of miR-134-5p | |
| HOXC11 | Target gene of miR-134-5p | |
| HOXD10 | Target gene of miR-134-5p | |
| HOXD3 | Target gene of miR-134-5p | |
| HPDL | Target gene of miR-134-5p | |
| HR | Target gene of miR-134-5p | |
| HRH2 | Target gene of miR-134-5p | |
| HRH4 | Target gene of miR-134-5p | |
| HRK | Target gene of miR-134-5p | |
| HS2ST1 | Target gene of miR-134-5p | |
| HS3ST3B1 | Target gene of miR-134-5p | |
| HS3ST4 | Target gene of miR-134-5p | |
| HS6ST3 | Target gene of miR-134-5p | |
| HSPA12A | Target gene of miR-134-5p | |
| HTD2 | Target gene of miR-134-5p | |
| HTR2C | Target gene of miR-134-5p | |
| HTR3B | Target gene of miR-134-5p | |
| IBA57 | Target gene of miR-134-5p | |
| IDE | Target gene of miR-134-5p | |
| IFNAR1 | Target gene of miR-134-5p | |
| IFNGR2 | Target gene of miR-134-5p | |
| IFNL3 | Target gene of miR-134-5p | |
| IFT74 | Target gene of miR-134-5p | |
| IGF2R | Target gene of miR-134-5p | |
| IGFBP5 | Target gene of miR-134-5p | |
| IGSF9B | Target gene of miR-134-5p | |
| IKZF2 | Target gene of miR-134-5p | |
| IKZF5 | Target gene of miR-134-5p | |
| IL10 | Target gene of miR-134-5p | |
| IL17D | Target gene of miR-134-5p | |
| IL17REL | Target gene of miR-134-5p | |
| IL1B | Target gene of miR-134-5p | |
| IL1R1 | Target gene of miR-134-5p | |
| IL27 | Target gene of miR-134-5p | |
| IL2RB | Target gene of miR-134-5p | |
| IL36RN | Target gene of miR-134-5p | |
| IL5RA | Target gene of miR-134-5p | |
| ILDR2 | Target gene of miR-134-5p | |
| IMPA1 | Target gene of miR-134-5p | |
| IMPACT | Target gene of miR-134-5p | |
| INAFM1 | Target gene of miR-134-5p | |
| INAFM2 | Target gene of miR-134-5p | |
| INMT | Target gene of miR-134-5p | |
| INPP5B | Target gene of miR-134-5p | |
| IQCE | Target gene of miR-134-5p | |
| IQCJ | Target gene of miR-134-5p | |
| IQGAP1 | Target gene of miR-134-5p | |
| IRAG1 | Target gene of miR-134-5p | |
| IRAK4 | Target gene of miR-134-5p | |
| IRF4 | Target gene of miR-134-5p | |
| ISCA2 | Target gene of miR-134-5p | |
| ISCU | Target gene of miR-134-5p | |
| ISY1 | Target gene of miR-134-5p | |
| ITGA1 | Target gene of miR-134-5p | |
| ITGA8 | Target gene of miR-134-5p | |
| ITM2C | Target gene of miR-134-5p | |
| IYD | Target gene of miR-134-5p | |
| JADE1 | Target gene of miR-134-5p | |
| JAKMIP2 | Target gene of miR-134-5p | |
| JAM2 | Target gene of miR-134-5p | |
| JCAD | Target gene of miR-134-5p | |
| JHY | Target gene of miR-134-5p | |
| JPH4 | Target gene of miR-134-5p | |
| KALRN | Target gene of miR-134-5p | |
| KANSL1L | Target gene of miR-134-5p | |
| KAT14 | Target gene of miR-134-5p | |
| KAT7 | Target gene of miR-134-5p | |
| KBTBD3 | Target gene of miR-134-5p | |
| KBTBD6 | Target gene of miR-134-5p | |
| KCNA1 | Target gene of miR-134-5p | |
| KCNG4 | Target gene of miR-134-5p | |
| KCNH1 | Target gene of miR-134-5p | |
| KCNJ11 | Target gene of miR-134-5p | |
| KCNK10 | Target gene of miR-134-5p | |
| KCNK12 | Target gene of miR-134-5p | |
| KCNMB1 | Target gene of miR-134-5p | |
| KCNMB4 | Target gene of miR-134-5p | |
| KCNN1 | Target gene of miR-134-5p | |
| KCNQ4 | Target gene of miR-134-5p | |
| KCNQ5 | Target gene of miR-134-5p | |
| KCNS1 | Target gene of miR-134-5p | |
| KCTD10 | Target gene of miR-134-5p | |
| KCTD20 | Target gene of miR-134-5p | |
| KDM5A | Target gene of miR-134-5p | |
| KEAP1 | Target gene of miR-134-5p | |
| KHDC4 | Target gene of miR-134-5p | |
| KIAA0753 | Target gene of miR-134-5p | |
| KIAA0930 | Target gene of miR-134-5p | |
| KIAA1671 | Target gene of miR-134-5p | |
| KIDINS220 | Target gene of miR-134-5p | |
| KIF1A | Target gene of miR-134-5p | |
| KIF1C | Target gene of miR-134-5p | |
| KIF27 | Target gene of miR-134-5p | |
| KIF5C | Target gene of miR-134-5p | |
| KIR2DS4 | Target gene of miR-134-5p | |
| KIRREL1 | Target gene of miR-134-5p | |
| KITLG | Target gene of miR-134-5p | |
| KLF12 | Target gene of miR-134-5p | |
| KLF7 | Target gene of miR-134-5p | |
| KLHL13 | Target gene of miR-134-5p | |
| KLHL14 | Target gene of miR-134-5p | |
| KLHL21 | Target gene of miR-134-5p | |
| KLHL3 | Target gene of miR-134-5p | |
| KLHL38 | Target gene of miR-134-5p | |
| KNG1 | Target gene of miR-134-5p | |
| KNL1 | Target gene of miR-134-5p | |
| KNOP1 | Target gene of miR-134-5p | |
| KNSTRN | Target gene of miR-134-5p | |
| KPNA6 | Target gene of miR-134-5p | |
| KPNB1 | Target gene of miR-134-5p | |
| KRT13 | Target gene of miR-134-5p | |
| KRT3 | Target gene of miR-134-5p | |
| KRTAP4-1 | Target gene of miR-134-5p | |
| KRTAP4-5 | Target gene of miR-134-5p | |
| KSR2 | Target gene of miR-134-5p | |
| L3MBTL4 | Target gene of miR-134-5p | |
| LAMP1 | Target gene of miR-134-5p | |
| LARGE1 | Target gene of miR-134-5p | |
| LCOR | Target gene of miR-134-5p | |
| LDAH | Target gene of miR-134-5p | |
| LEXM | Target gene of miR-134-5p | |
| LGI2 | Target gene of miR-134-5p | |
| LHX6 | Target gene of miR-134-5p | |
| LHX9 | Target gene of miR-134-5p | |
| LIN52 | Target gene of miR-134-5p | |
| LINGO2 | Target gene of miR-134-5p | |
| LLCFC1 | Target gene of miR-134-5p | |
| LMCD1 | Target gene of miR-134-5p | |
| LMTK2 | Target gene of miR-134-5p | |
| LOC102723502 | Target gene of miR-134-5p | |
| LOC102723996 | Target gene of miR-134-5p | |
| LOC102724250 | Target gene of miR-134-5p | |
| LOC102724488 | Target gene of miR-134-5p | |
| LOC389895 | Target gene of miR-134-5p | |
| LOC401040 | Target gene of miR-134-5p | |
| LPIN2 | Target gene of miR-134-5p | |
| LRATD1 | Target gene of miR-134-5p | |
| LRIG2 | Target gene of miR-134-5p | |
| LRP6 | Target gene of miR-134-5p | |
| LRPAP1 | Target gene of miR-134-5p | |
| LRRC10 | Target gene of miR-134-5p | |
| LRRC14 | Target gene of miR-134-5p | |
| LRRC19 | Target gene of miR-134-5p | |
| LRRC2 | Target gene of miR-134-5p | |
| LRRC37A3 | Target gene of miR-134-5p | |
| LRRC39 | Target gene of miR-134-5p | |
| LRRC40 | Target gene of miR-134-5p | |
| LRRC55 | Target gene of miR-134-5p | |
| LRRC57 | Target gene of miR-134-5p | |
| LRRTM2 | Target gene of miR-134-5p | |
| LRTM2 | Target gene of miR-134-5p | |
| LSAMP | Target gene of miR-134-5p | |
| LSM11 | Target gene of miR-134-5p | |
| LSM6 | Target gene of miR-134-5p | |
| LTO1 | Target gene of miR-134-5p | |
| LUC7L2 | Target gene of miR-134-5p | |
| LY6G5C | Target gene of miR-134-5p | |
| LYPD6B | Target gene of miR-134-5p | |
| LYRM7 | Target gene of miR-134-5p | |
| MADD | Target gene of miR-134-5p | |
| MAFB | Target gene of miR-134-5p | |
| MAGEH1 | Target gene of miR-134-5p | |
| MAN1A1 | Target gene of miR-134-5p | |
| MAP1LC3B | Target gene of miR-134-5p | |
| MAP1LC3B2 | Target gene of miR-134-5p | |
| MAP2K6 | Target gene of miR-134-5p | |
| MAP3K20 | Target gene of miR-134-5p | |
| MAP3K9 | Target gene of miR-134-5p | |
| MAP6 | Target gene of miR-134-5p | |
| MAP6D1 | Target gene of miR-134-5p | |
| MAPK13 | Target gene of miR-134-5p | |
| MAPK14 | Target gene of miR-134-5p | |
| MAPK9 | Target gene of miR-134-5p | |
| MAPKBP1 | Target gene of miR-134-5p | |
| MAPRE2 | Target gene of miR-134-5p | |
| MAPRE3 | Target gene of miR-134-5p | |
| MAPT | Target gene of miR-134-5p | |
| MARCHF3 | Target gene of miR-134-5p | |
| MARK2 | Target gene of miR-134-5p | |
| MARK3 | Target gene of miR-134-5p | |
| MARS2 | Target gene of miR-134-5p | |
| MARVELD2 | Target gene of miR-134-5p | |
| MASP1 | Target gene of miR-134-5p | |
| MAVS | Target gene of miR-134-5p | |
| MBD1 | Target gene of miR-134-5p | |
| MBD3 | Target gene of miR-134-5p | |
| MBOAT7 | Target gene of miR-134-5p | |
| MCC | Target gene of miR-134-5p | |
| MCIDAS | Target gene of miR-134-5p | |
| MCMBP | Target gene of miR-134-5p | |
| MDN1 | Target gene of miR-134-5p | |
| MECR | Target gene of miR-134-5p | |
| MED22 | Target gene of miR-134-5p | |
| MED28 | Target gene of miR-134-5p | |
| MEF2A | Target gene of miR-134-5p | |
| MEGF9 | Target gene of miR-134-5p | |
| MEP1A | Target gene of miR-134-5p | |
| METAP2 | Target gene of miR-134-5p | |
| METTL22 | Target gene of miR-134-5p | |
| METTL4 | Target gene of miR-134-5p | |
| METTL7B | Target gene of miR-134-5p | |
| MEX3A | Target gene of miR-134-5p | |
| MFAP3L | Target gene of miR-134-5p | |
| MFN2 | Target gene of miR-134-5p | |
| MFSD4B | Target gene of miR-134-5p | |
| MGLL | Target gene of miR-134-5p | |
| MGME1 | Target gene of miR-134-5p | |
| MICAL3 | Target gene of miR-134-5p | |
| MICOS10 | Target gene of miR-134-5p | |
| MID2 | Target gene of miR-134-5p | |
| MIEF1 | Target gene of miR-134-5p | |
| MIGA2 | Target gene of miR-134-5p | |
| MINAR2 | Target gene of miR-134-5p | |
| MIS12 | Target gene of miR-134-5p | |
| MKLN1 | Target gene of miR-134-5p | |
| MKNK2 | Target gene of miR-134-5p | |
| MKS1 | Target gene of miR-134-5p | |
| MLEC | Target gene of miR-134-5p | |
| MLIP | Target gene of miR-134-5p | |
| MMAB | Target gene of miR-134-5p | |
| MMP15 | Target gene of miR-134-5p | |
| MMP24 | Target gene of miR-134-5p | |
| MMP28 | Target gene of miR-134-5p | |
| MOK | Target gene of miR-134-5p | |
| MON1B | Target gene of miR-134-5p | |
| MON2 | Target gene of miR-134-5p | |
| MORC3 | Target gene of miR-134-5p | |
| MOSMO | Target gene of miR-134-5p | |
| MPIG6B | Target gene of miR-134-5p | |
| MPP2 | Target gene of miR-134-5p | |
| MRAS | Target gene of miR-134-5p | |
| MRM1 | Target gene of miR-134-5p | |
| MRM2 | Target gene of miR-134-5p | |
| MRO | Target gene of miR-134-5p | |
| MRPS11 | Target gene of miR-134-5p | |
| MRPS27 | Target gene of miR-134-5p | |
| MSANTD3 | Target gene of miR-134-5p | |
| MSI1 | Target gene of miR-134-5p | |
| MSMB | Target gene of miR-134-5p | |
| MSR1 | Target gene of miR-134-5p | |
| MSRB1 | Target gene of miR-134-5p | |
| MTA3 | Target gene of miR-134-5p | |
| MTARC1 | Target gene of miR-134-5p | |
| MTERF1 | Target gene of miR-134-5p | |
| MTHFSD | Target gene of miR-134-5p | |
| MTMR2 | Target gene of miR-134-5p | |
| MTMR6 | Target gene of miR-134-5p | |
| MTO1 | Target gene of miR-134-5p | |
| MUC7 | Target gene of miR-134-5p | |
| MYADM | Target gene of miR-134-5p | |
| MYCBPAP | Target gene of miR-134-5p | |
| MYCN | Target gene of miR-134-5p | |
| MYEF2 | Target gene of miR-134-5p | |
| MYEOV | Target gene of miR-134-5p | |
| MYH9 | Target gene of miR-134-5p | |
| MYO18A | Target gene of miR-134-5p | |
| MYO19 | Target gene of miR-134-5p | |
| MYO1C | Target gene of miR-134-5p | |
| MYO5A | Target gene of miR-134-5p | |
| MYO7A | Target gene of miR-134-5p | |
| MYOCD | Target gene of miR-134-5p | |
| NAA40 | Target gene of miR-134-5p | |
| NACA | Target gene of miR-134-5p | |
| NAGA | Target gene of miR-134-5p | |
| NANOG | Target gene of miR-134-5p | |
| NAPB | Target gene of miR-134-5p | |
| NARF | Target gene of miR-134-5p | |
| NAT9 | Target gene of miR-134-5p | |
| NATD1 | Target gene of miR-134-5p | |
| NBPF10 | Target gene of miR-134-5p | |
| NBPF11 | Target gene of miR-134-5p | |
| NBPF12 | Target gene of miR-134-5p | |
| NBPF14 | Target gene of miR-134-5p | |
| NBPF15 | Target gene of miR-134-5p | |
| NBPF19 | Target gene of miR-134-5p | |
| NBPF3 | Target gene of miR-134-5p | |
| NBPF9 | Target gene of miR-134-5p | |
| NCAPH2 | Target gene of miR-134-5p | |
| NCBP3 | Target gene of miR-134-5p | |
| NCEH1 | Target gene of miR-134-5p | |
| NCKAP1L | Target gene of miR-134-5p | |
| NCMAP | Target gene of miR-134-5p | |
| NCSTN | Target gene of miR-134-5p | |
| NDRG4 | Target gene of miR-134-5p | |
| NDUFAF7 | Target gene of miR-134-5p | |
| NECTIN1 | Target gene of miR-134-5p | |
| NECTIN4 | Target gene of miR-134-5p | |
| NEGR1 | Target gene of miR-134-5p | |
| NEK2 | Target gene of miR-134-5p | |
| NEK5 | Target gene of miR-134-5p | |
| NEK6 | Target gene of miR-134-5p | |
| NEMP1 | Target gene of miR-134-5p | |
| NEPRO | Target gene of miR-134-5p | |
| NETO1 | Target gene of miR-134-5p | |
| NEU3 | Target gene of miR-134-5p | |
| NEURL1 | Target gene of miR-134-5p | |
| NFATC1 | Target gene of miR-134-5p | |
| NFATC2 | Target gene of miR-134-5p | |
| NHEJ1 | Target gene of miR-134-5p | |
| NIBAN2 | Target gene of miR-134-5p | |
| NIPA1 | Target gene of miR-134-5p | |
| NIPAL1 | Target gene of miR-134-5p | |
| NIPAL2 | Target gene of miR-134-5p | |
| NIPAL4 | Target gene of miR-134-5p | |
| NKAIN4 | Target gene of miR-134-5p | |
| NKX1-2 | Target gene of miR-134-5p | |
| NKX2-6 | Target gene of miR-134-5p | |
| NKX3-1 | Target gene of miR-134-5p | |
| NLRP2B | Target gene of miR-134-5p | |
| NLRP4 | Target gene of miR-134-5p | |
| NME4 | Target gene of miR-134-5p | |
| NME6 | Target gene of miR-134-5p | |
| NMUR2 | Target gene of miR-134-5p | |
| NNAT | Target gene of miR-134-5p | |
| NOL12 | Target gene of miR-134-5p | |
| NOL4 | Target gene of miR-134-5p | |
| NOL7 | Target gene of miR-134-5p | |
| NOP14 | Target gene of miR-134-5p | |
| NOP9 | Target gene of miR-134-5p | |
| NOPCHAP1 | Target gene of miR-134-5p | |
| NOTO | Target gene of miR-134-5p | |
| NPHS2 | Target gene of miR-134-5p | |
| NPR3 | Target gene of miR-134-5p | |
| NPTXR | Target gene of miR-134-5p | |
| NR2E1 | Target gene of miR-134-5p | |
| NRG1 | Target gene of miR-134-5p | |
| NRL | Target gene of miR-134-5p | |
| NSD1 | Target gene of miR-134-5p | |
| NSMCE3 | Target gene of miR-134-5p | |
| NSUN4 | Target gene of miR-134-5p | |
| NSUN5 | Target gene of miR-134-5p | |
| NT5C1A | Target gene of miR-134-5p | |
| NTRK3 | Target gene of miR-134-5p | |
| NUDT4 | Target gene of miR-134-5p | |
| NUPR2 | Target gene of miR-134-5p | |
| NUSAP1 | Target gene of miR-134-5p | |
| NVL | Target gene of miR-134-5p | |
| NXPE1 | Target gene of miR-134-5p | |
| OAF | Target gene of miR-134-5p | |
| OASL | Target gene of miR-134-5p | |
| OGFOD3 | Target gene of miR-134-5p | |
| OLFM1 | Target gene of miR-134-5p | |
| OLFM2 | Target gene of miR-134-5p | |
| OLR1 | Target gene of miR-134-5p | |
| ONECUT2 | Target gene of miR-134-5p | |
| OPALIN | Target gene of miR-134-5p | |
| OPRM1 | Target gene of miR-134-5p | |
| OR14I1 | Target gene of miR-134-5p | |
| OR14J1 | Target gene of miR-134-5p | |
| OR2AG1 | Target gene of miR-134-5p | |
| OR2C3 | Target gene of miR-134-5p | |
| OR2T1 | Target gene of miR-134-5p | |
| OR2T10 | Target gene of miR-134-5p | |
| OR2T12 | Target gene of miR-134-5p | |
| OR2T2 | Target gene of miR-134-5p | |
| OR2T35 | Target gene of miR-134-5p | |
| OR2V1 | Target gene of miR-134-5p | |
| OR4D1 | Target gene of miR-134-5p | |
| OR52A1 | Target gene of miR-134-5p | |
| OR52K1 | Target gene of miR-134-5p | |
| OR56A1 | Target gene of miR-134-5p | |
| OR5H14 | Target gene of miR-134-5p | |
| OR5K1 | Target gene of miR-134-5p | |
| OR6J1 | Target gene of miR-134-5p | |
| OR6Y1 | Target gene of miR-134-5p | |
| OR7G2 | Target gene of miR-134-5p | |
| OR8B8 | Target gene of miR-134-5p | |
| OR9I1 | Target gene of miR-134-5p | |
| OR9K2 | Target gene of miR-134-5p | |
| ORMDL1 | Target gene of miR-134-5p | |
| OS9 | Target gene of miR-134-5p | |
| OSBPL10 | Target gene of miR-134-5p | |
| OSER1 | Target gene of miR-134-5p | |
| OSTF1 | Target gene of miR-134-5p | |
| OTULIN | Target gene of miR-134-5p | |
| OTX1 | Target gene of miR-134-5p | |
| OXLD1 | Target gene of miR-134-5p | |
| PABIR3 | Target gene of miR-134-5p | |
| PABPC1 | Target gene of miR-134-5p | |
| PALM2AKAP2 | Target gene of miR-134-5p | |
| PAN2 | Target gene of miR-134-5p | |
| PANX2 | Target gene of miR-134-5p | |
| PAOX | Target gene of miR-134-5p | |
| PAPLN | Target gene of miR-134-5p | |
| PAQR4 | Target gene of miR-134-5p | |
| PAQR7 | Target gene of miR-134-5p | |
| PARD3B | Target gene of miR-134-5p | |
| PARVB | Target gene of miR-134-5p | |
| PAX7 | Target gene of miR-134-5p | |
| PBDC1 | Target gene of miR-134-5p | |
| PCCA | Target gene of miR-134-5p | |
| PCDH12 | Target gene of miR-134-5p | |
| PCDH9 | Target gene of miR-134-5p | |
| PCDHA4 | Target gene of miR-134-5p | |
| PCDHB4 | Target gene of miR-134-5p | |
| PCDHGA1 | Target gene of miR-134-5p | |
| PCDHGA8 | Target gene of miR-134-5p | |
| PCDHGC4 | Target gene of miR-134-5p | |
| PCDHGC5 | Target gene of miR-134-5p | |
| PCGF3 | Target gene of miR-134-5p | |
| PCLO | Target gene of miR-134-5p | |
| PCMTD2 | Target gene of miR-134-5p | |
| PCNX1 | Target gene of miR-134-5p | |
| PCNX4 | Target gene of miR-134-5p | |
| PCSK5 | Target gene of miR-134-5p | |
| PCYT1A | Target gene of miR-134-5p | |
| PDCD7 | Target gene of miR-134-5p | |
| PDCL | Target gene of miR-134-5p | |
| PDE1C | Target gene of miR-134-5p | |
| PDE4DIP | Target gene of miR-134-5p | |
| PDE7A | Target gene of miR-134-5p | |
| PDGFB | Target gene of miR-134-5p | |
| PDGFD | Target gene of miR-134-5p | |
| PDHA1 | Target gene of miR-134-5p | |
| PDIA3 | Target gene of miR-134-5p | |
| PDK2 | Target gene of miR-134-5p | |
| PDK3 | Target gene of miR-134-5p | |
| PEX26 | Target gene of miR-134-5p | |
| PFKFB4 | Target gene of miR-134-5p | |
| PGBD5 | Target gene of miR-134-5p | |
| PHACTR1 | Target gene of miR-134-5p | |
| PHETA2 | Target gene of miR-134-5p | |
| PHF12 | Target gene of miR-134-5p | |
| PHF19 | Target gene of miR-134-5p | |
| PIAS1 | Target gene of miR-134-5p | |
| PIGL | Target gene of miR-134-5p | |
| PIGM | Target gene of miR-134-5p | |
| PIK3C3 | Target gene of miR-134-5p | |
| PIN4 | Target gene of miR-134-5p | |
| PIRT | Target gene of miR-134-5p | |
| PITPNA | Target gene of miR-134-5p | |
| PLA2G15 | Target gene of miR-134-5p | |
| PLA2G4E | Target gene of miR-134-5p | |
| PLAGL2 | Target gene of miR-134-5p | |
| PLAT | Target gene of miR-134-5p | |
| PLCXD1 | Target gene of miR-134-5p | |
| PLEKHA6 | Target gene of miR-134-5p | |
| PLEKHD1 | Target gene of miR-134-5p | |
| PLEKHG2 | Target gene of miR-134-5p | |
| PLPBP | Target gene of miR-134-5p | |
| PLPP7 | Target gene of miR-134-5p | |
| PLSCR4 | Target gene of miR-134-5p | |
| PLTP | Target gene of miR-134-5p | |
| PLXNA3 | Target gene of miR-134-5p | |
| PMPCB | Target gene of miR-134-5p | |
| PMVK | Target gene of miR-134-5p | |
| PODNL1 | Target gene of miR-134-5p | |
| PODXL | Target gene of miR-134-5p | |
| POGZ | Target gene of miR-134-5p | |
| POLD3 | Target gene of miR-134-5p | |
| POLD4 | Target gene of miR-134-5p | |
| POLL | Target gene of miR-134-5p | |
| POLR3G | Target gene of miR-134-5p | |
| POTEA | Target gene of miR-134-5p | |
| POTEB3 | Target gene of miR-134-5p | |
| POTEC | Target gene of miR-134-5p | |
| POTED | Target gene of miR-134-5p | |
| POTEM | Target gene of miR-134-5p | |
| POU2F1 | Target gene of miR-134-5p | |
| PPARA | Target gene of miR-134-5p | |
| PPARG | Target gene of miR-134-5p | |
| PPIA | Target gene of miR-134-5p | |
| PPIAL4A | Target gene of miR-134-5p | |
| PPIAL4D | Target gene of miR-134-5p | |
| PPIAL4E | Target gene of miR-134-5p | |
| PPIAL4F | Target gene of miR-134-5p | |
| PPIAL4H | Target gene of miR-134-5p | |
| PPIL2 | Target gene of miR-134-5p | |
| PPM1A | Target gene of miR-134-5p | |
| PPP1R18 | Target gene of miR-134-5p | |
| PPP2R2B | Target gene of miR-134-5p | |
| PPP4R3A | Target gene of miR-134-5p | |
| PPP6C | Target gene of miR-134-5p | |
| PRCP | Target gene of miR-134-5p | |
| PRDM5 | Target gene of miR-134-5p | |
| PRELID2 | Target gene of miR-134-5p | |
| PRLHR | Target gene of miR-134-5p | |
| PROP1 | Target gene of miR-134-5p | |
| PRORP | Target gene of miR-134-5p | |
| PROSER2 | Target gene of miR-134-5p | |
| PROSER3 | Target gene of miR-134-5p | |
| PROX2 | Target gene of miR-134-5p | |
| PRPH | Target gene of miR-134-5p | |
| PRR5L | Target gene of miR-134-5p | |
| PRSS16 | Target gene of miR-134-5p | |
| PRUNE2 | Target gene of miR-134-5p | |
| PRXL2A | Target gene of miR-134-5p | |
| PSD4 | Target gene of miR-134-5p | |
| PSIP1 | Target gene of miR-134-5p | |
| PSMD12 | Target gene of miR-134-5p | |
| PSMD5 | Target gene of miR-134-5p | |
| PSMF1 | Target gene of miR-134-5p | |
| PTAR1 | Target gene of miR-134-5p | |
| PTBP3 | Target gene of miR-134-5p | |
| PTGDR2 | Target gene of miR-134-5p | |
| PTHLH | Target gene of miR-134-5p | |
| PTPN14 | Target gene of miR-134-5p | |
| PTPRJ | Target gene of miR-134-5p | |
| PTPRT | Target gene of miR-134-5p | |
| PUDP | Target gene of miR-134-5p | |
| PWWP3B | Target gene of miR-134-5p | |
| PXMP4 | Target gene of miR-134-5p | |
| PYHIN1 | Target gene of miR-134-5p | |
| QKI | Target gene of miR-134-5p | |
| QRFPR | Target gene of miR-134-5p | |
| QSOX1 | Target gene of miR-134-5p | |
| RAB11FIP5 | Target gene of miR-134-5p | |
| RAB21 | Target gene of miR-134-5p | |
| RAB22A | Target gene of miR-134-5p | |
| RAB27A | Target gene of miR-134-5p | |
| RAB29 | Target gene of miR-134-5p | |
| RAB30 | Target gene of miR-134-5p | |
| RAB33B | Target gene of miR-134-5p | |
| RAB36 | Target gene of miR-134-5p | |
| RAB3A | Target gene of miR-134-5p | |
| RAB3B | Target gene of miR-134-5p | |
| RAB40A | Target gene of miR-134-5p | |
| RAB7B | Target gene of miR-134-5p | |
| RABGAP1L | Target gene of miR-134-5p | |
| RABIF | Target gene of miR-134-5p | |
| RABL2A | Target gene of miR-134-5p | |
| RABL2B | Target gene of miR-134-5p | |
| RACGAP1 | Target gene of miR-134-5p | |
| RAD23B | Target gene of miR-134-5p | |
| RAD51 | Target gene of miR-134-5p | |
| RAD51D | Target gene of miR-134-5p | |
| RANBP3 | Target gene of miR-134-5p | |
| RAP1A | Target gene of miR-134-5p | |
| RAP1B | Target gene of miR-134-5p | |
| RAP2B | Target gene of miR-134-5p | |
| RAPGEF3 | Target gene of miR-134-5p | |
| RAPGEF6 | Target gene of miR-134-5p | |
| RARA | Target gene of miR-134-5p | |
| RASAL1 | Target gene of miR-134-5p | |
| RASGRF2 | Target gene of miR-134-5p | |
| RASSF3 | Target gene of miR-134-5p | |
| RASSF4 | Target gene of miR-134-5p | |
| RBBP4 | Target gene of miR-134-5p | |
| RBBP5 | Target gene of miR-134-5p | |
| RBM12 | Target gene of miR-134-5p | |
| RBM12B | Target gene of miR-134-5p | |
| RBM28 | Target gene of miR-134-5p | |
| RBMS1 | Target gene of miR-134-5p | |
| RBMS2 | Target gene of miR-134-5p | |
| RBMS3 | Target gene of miR-134-5p | |
| RC3H1 | Target gene of miR-134-5p | |
| RCAN1 | Target gene of miR-134-5p | |
| RCAN2 | Target gene of miR-134-5p | |
| RCBTB1 | Target gene of miR-134-5p | |
| RCOR1 | Target gene of miR-134-5p | |
| RCOR3 | Target gene of miR-134-5p | |
| RD3 | Target gene of miR-134-5p | |
| REST | Target gene of miR-134-5p | |
| REXO2 | Target gene of miR-134-5p | |
| RFC2 | Target gene of miR-134-5p | |
| RFC5 | Target gene of miR-134-5p | |
| RFFL | Target gene of miR-134-5p | |
| RFXANK | Target gene of miR-134-5p | |
| RGL1 | Target gene of miR-134-5p | |
| RGR | Target gene of miR-134-5p | |
| RGS11 | Target gene of miR-134-5p | |
| RGS8 | Target gene of miR-134-5p | |
| RHBDD1 | Target gene of miR-134-5p | |
| RHBDF2 | Target gene of miR-134-5p | |
| RHOA | Target gene of miR-134-5p | |
| RHOBTB2 | Target gene of miR-134-5p | |
| RHOU | Target gene of miR-134-5p | |
| RIIAD1 | Target gene of miR-134-5p | |
| RIMS4 | Target gene of miR-134-5p | |
| RIOK1 | Target gene of miR-134-5p | |
| RIPOR2 | Target gene of miR-134-5p | |
| RMND5A | Target gene of miR-134-5p | |
| RNASE7 | Target gene of miR-134-5p | |
| RNF115 | Target gene of miR-134-5p | |
| RNF125 | Target gene of miR-134-5p | |
| RNF144A | Target gene of miR-134-5p | |
| RNF152 | Target gene of miR-134-5p | |
| RNF157 | Target gene of miR-134-5p | |
| RNF19A | Target gene of miR-134-5p | |
| RNF214 | Target gene of miR-134-5p | |
| RNF217 | Target gene of miR-134-5p | |
| RNF24 | Target gene of miR-134-5p | |
| RNF38 | Target gene of miR-134-5p | |
| RNF41 | Target gene of miR-134-5p | |
| RNF8 | Target gene of miR-134-5p | |
| RO60 | Target gene of miR-134-5p | |
| RP1 | Target gene of miR-134-5p | |
| RPGRIP1L | Target gene of miR-134-5p | |
| RPL10 | Target gene of miR-134-5p | |
| RPL14 | Target gene of miR-134-5p | |
| RPP14 | Target gene of miR-134-5p | |
| RPS29 | Target gene of miR-134-5p | |
| RPS4Y1 | Target gene of miR-134-5p | |
| RPS6KA1 | Target gene of miR-134-5p | |
| RRP8 | Target gene of miR-134-5p | |
| RSAD2 | Target gene of miR-134-5p | |
| RSPH1 | Target gene of miR-134-5p | |
| RSPO4 | Target gene of miR-134-5p | |
| RTL3 | Target gene of miR-134-5p | |
| RTL5 | Target gene of miR-134-5p | |
| RTL6 | Target gene of miR-134-5p | |
| RTL8B | Target gene of miR-134-5p | |
| RTL8C | Target gene of miR-134-5p | |
| RTP2 | Target gene of miR-134-5p | |
| RUBCNL | Target gene of miR-134-5p | |
| RUNX1 | Target gene of miR-134-5p | |
| RUNX3 | Target gene of miR-134-5p | |
| RUVBL1 | Target gene of miR-134-5p | |
| RXFP1 | Target gene of miR-134-5p | |
| RXRA | Target gene of miR-134-5p | |
| S100PBP | Target gene of miR-134-5p | |
| SACM1L | Target gene of miR-134-5p | |
| SAMD12 | Target gene of miR-134-5p | |
| SAMD7 | Target gene of miR-134-5p | |
| SANBR | Target gene of miR-134-5p | |
| SAP30L | Target gene of miR-134-5p | |
| SARM1 | Target gene of miR-134-5p | |
| SC5D | Target gene of miR-134-5p | |
| SCAI | Target gene of miR-134-5p | |
| SCAMP5 | Target gene of miR-134-5p | |
| SCARA5 | Target gene of miR-134-5p | |
| SCIMP | Target gene of miR-134-5p | |
| SCN2A | Target gene of miR-134-5p | |
| SCRN2 | Target gene of miR-134-5p | |
| SCRT2 | Target gene of miR-134-5p | |
| SDCBP | Target gene of miR-134-5p | |
| SDK1 | Target gene of miR-134-5p | |
| SEC14L2 | Target gene of miR-134-5p | |
| SEC14L3 | Target gene of miR-134-5p | |
| SELENOI | Target gene of miR-134-5p | |
| SELENON | Target gene of miR-134-5p | |
| SELENOP | Target gene of miR-134-5p | |
| SELENOT | Target gene of miR-134-5p | |
| SEMA3D | Target gene of miR-134-5p | |
| SEMA4F | Target gene of miR-134-5p | |
| SEMA5B | Target gene of miR-134-5p | |
| SEPSECS | Target gene of miR-134-5p | |
| SEPTIN10 | Target gene of miR-134-5p | |
| SEPTIN2 | Target gene of miR-134-5p | |
| SEPTIN6 | Target gene of miR-134-5p | |
| SERPINB8 | Target gene of miR-134-5p | |
| SERTM2 | Target gene of miR-134-5p | |
| SESN3 | Target gene of miR-134-5p | |
| SESTD1 | Target gene of miR-134-5p | |
| SET | Target gene of miR-134-5p | |
| SFMBT2 | Target gene of miR-134-5p | |
| SFRP1 | Target gene of miR-134-5p | |
| SFXN2 | Target gene of miR-134-5p | |
| SGCD | Target gene of miR-134-5p | |
| SGMS1 | Target gene of miR-134-5p | |
| SGO1 | Target gene of miR-134-5p | |
| SH2B1 | Target gene of miR-134-5p | |
| SH3BP5 | Target gene of miR-134-5p | |
| SHISA7 | Target gene of miR-134-5p | |
| SHOC1 | Target gene of miR-134-5p | |
| SHPRH | Target gene of miR-134-5p | |
| SHQ1 | Target gene of miR-134-5p | |
| SHTN1 | Target gene of miR-134-5p | |
| SIAH3 | Target gene of miR-134-5p | |
| SIDT2 | Target gene of miR-134-5p | |
| SIGLECL1 | Target gene of miR-134-5p | |
| SIKE1 | Target gene of miR-134-5p | |
| SIMC1 | Target gene of miR-134-5p | |
| SIRPB2 | Target gene of miR-134-5p | |
| SIRT5 | Target gene of miR-134-5p | |
| SLAMF8 | Target gene of miR-134-5p | |
| SLBP | Target gene of miR-134-5p | |
| SLC10A7 | Target gene of miR-134-5p | |
| SLC12A6 | Target gene of miR-134-5p | |
| SLC13A2 | Target gene of miR-134-5p | |
| SLC16A12 | Target gene of miR-134-5p | |
| SLC17A1 | Target gene of miR-134-5p | |
| SLC17A6 | Target gene of miR-134-5p | |
| SLC17A9 | Target gene of miR-134-5p | |
| SLC1A2 | Target gene of miR-134-5p | |
| SLC22A25 | Target gene of miR-134-5p | |
| SLC22A7 | Target gene of miR-134-5p | |
| SLC24A2 | Target gene of miR-134-5p | |
| SLC25A21 | Target gene of miR-134-5p | |
| SLC25A25 | Target gene of miR-134-5p | |
| SLC25A3 | Target gene of miR-134-5p | |
| SLC29A4 | Target gene of miR-134-5p | |
| SLC2A13 | Target gene of miR-134-5p | |
| SLC2A8 | Target gene of miR-134-5p | |
| SLC30A1 | Target gene of miR-134-5p | |
| SLC30A2 | Target gene of miR-134-5p | |
| SLC30A4 | Target gene of miR-134-5p | |
| SLC35A2 | Target gene of miR-134-5p | |
| SLC35A3 | Target gene of miR-134-5p | |
| SLC35B2 | Target gene of miR-134-5p | |
| SLC35B4 | Target gene of miR-134-5p | |
| SLC35D1 | Target gene of miR-134-5p | |
| SLC35E3 | Target gene of miR-134-5p | |
| SLC35F5 | Target gene of miR-134-5p | |
| SLC35F6 | Target gene of miR-134-5p | |
| SLC36A4 | Target gene of miR-134-5p | |
| SLC38A4 | Target gene of miR-134-5p | |
| SLC38A7 | Target gene of miR-134-5p | |
| SLC41A3 | Target gene of miR-134-5p | |
| SLC43A1 | Target gene of miR-134-5p | |
| SLC43A2 | Target gene of miR-134-5p | |
| SLC5A3 | Target gene of miR-134-5p | |
| SLC6A11 | Target gene of miR-134-5p | |
| SLC6A17 | Target gene of miR-134-5p | |
| SLC8A3 | Target gene of miR-134-5p | |
| SLC9A7 | Target gene of miR-134-5p | |
| SLC9B2 | Target gene of miR-134-5p | |
| SLF2 | Target gene of miR-134-5p | |
| SLFN12L | Target gene of miR-134-5p | |
| SLFN13 | Target gene of miR-134-5p | |
| SLFN14 | Target gene of miR-134-5p | |
| SLIT3 | Target gene of miR-134-5p | |
| SLITRK1 | Target gene of miR-134-5p | |
| SLITRK3 | Target gene of miR-134-5p | |
| SLK | Target gene of miR-134-5p | |
| SMDT1 | Target gene of miR-134-5p | |
| SMIM14 | Target gene of miR-134-5p | |
| SMIM20 | Target gene of miR-134-5p | |
| SMIM33 | Target gene of miR-134-5p | |
| SMIM34A | Target gene of miR-134-5p | |
| SMIM34B | Target gene of miR-134-5p | |
| SMIM38 | Target gene of miR-134-5p | |
| SMIM40 | Target gene of miR-134-5p | |
| SMIM8 | Target gene of miR-134-5p | |
| SMPD4 | Target gene of miR-134-5p | |
| SMTNL2 | Target gene of miR-134-5p | |
| SMURF1 | Target gene of miR-134-5p | |
| SMURF2 | Target gene of miR-134-5p | |
| SNAP29 | Target gene of miR-134-5p | |
| SNED1 | Target gene of miR-134-5p | |
| SNX11 | Target gene of miR-134-5p | |
| SNX30 | Target gene of miR-134-5p | |
| SNX9 | Target gene of miR-134-5p | |
| SOCS1 | Target gene of miR-134-5p | |
| SON | Target gene of miR-134-5p | |
| SOWAHC | Target gene of miR-134-5p | |
| SOX17 | Target gene of miR-134-5p | |
| SP110 | Target gene of miR-134-5p | |
| SPA17 | Target gene of miR-134-5p | |
| SPATA12 | Target gene of miR-134-5p | |
| SPATA18 | Target gene of miR-134-5p | |
| SPATA2 | Target gene of miR-134-5p | |
| SPATA6 | Target gene of miR-134-5p | |
| SPATA6L | Target gene of miR-134-5p | |
| SPEF2 | Target gene of miR-134-5p | |
| SPINK5 | Target gene of miR-134-5p | |
| SPINK9 | Target gene of miR-134-5p | |
| SPPL2A | Target gene of miR-134-5p | |
| SPRR2A | Target gene of miR-134-5p | |
| SPRR2B | Target gene of miR-134-5p | |
| SPRR2D | Target gene of miR-134-5p | |
| SPRY3 | Target gene of miR-134-5p | |
| SPRYD4 | Target gene of miR-134-5p | |
| SPTAN1 | Target gene of miR-134-5p | |
| SPTBN2 | Target gene of miR-134-5p | |
| SRGAP2 | Target gene of miR-134-5p | |
| SRL | Target gene of miR-134-5p | |
| SRSF2 | Target gene of miR-134-5p | |
| SRSF3 | Target gene of miR-134-5p | |
| SRSF4 | Target gene of miR-134-5p | |
| SSBP2 | Target gene of miR-134-5p | |
| SSNA1 | Target gene of miR-134-5p | |
| SSTR3 | Target gene of miR-134-5p | |
| SSUH2 | Target gene of miR-134-5p | |
| SSX2IP | Target gene of miR-134-5p | |
| SSX4 | Target gene of miR-134-5p | |
| SSX4B | Target gene of miR-134-5p | |
| SSX5 | Target gene of miR-134-5p | |
| ST3GAL3 | Target gene of miR-134-5p | |
| ST3GAL4 | Target gene of miR-134-5p | |
| ST6GAL1 | Target gene of miR-134-5p | |
| ST8SIA1 | Target gene of miR-134-5p | |
| ST8SIA2 | Target gene of miR-134-5p | |
| ST8SIA3 | Target gene of miR-134-5p | |
| STAC2 | Target gene of miR-134-5p | |
| STAMBP | Target gene of miR-134-5p | |
| STAMBPL1 | Target gene of miR-134-5p | |
| STEAP3 | Target gene of miR-134-5p | |
| STIM1 | Target gene of miR-134-5p | |
| STING1 | Target gene of miR-134-5p | |
| STK24 | Target gene of miR-134-5p | |
| STK4 | Target gene of miR-134-5p | |
| STMN4 | Target gene of miR-134-5p | |
| STN1 | Target gene of miR-134-5p | |
| STON1 | Target gene of miR-134-5p | |
| STPG1 | Target gene of miR-134-5p | |
| STRIP1 | Target gene of miR-134-5p | |
| STRIP2 | Target gene of miR-134-5p | |
| STRN | Target gene of miR-134-5p | |
| STS | Target gene of miR-134-5p | |
| STX16 | Target gene of miR-134-5p | |
| STX2 | Target gene of miR-134-5p | |
| STX7 | Target gene of miR-134-5p | |
| STXBP5L | Target gene of miR-134-5p | |
| SUCNR1 | Target gene of miR-134-5p | |
| SULT1C2 | Target gene of miR-134-5p | |
| SUMF1 | Target gene of miR-134-5p | |
| SUMO2 | Target gene of miR-134-5p | |
| SUMO3 | Target gene of miR-134-5p | |
| SUPT20HL1 | Target gene of miR-134-5p | |
| SUPT20HL2 | Target gene of miR-134-5p | |
| SUPT3H | Target gene of miR-134-5p | |
| SUSD4 | Target gene of miR-134-5p | |
| SYK | Target gene of miR-134-5p | |
| SYN2 | Target gene of miR-134-5p | |
| SYN3 | Target gene of miR-134-5p | |
| SYNGR3 | Target gene of miR-134-5p | |
| SYNJ2BP-COX16 | Target gene of miR-134-5p | |
| SYNPO2 | Target gene of miR-134-5p | |
| SYNRG | Target gene of miR-134-5p | |
| SYPL2 | Target gene of miR-134-5p | |
| SYS1 | Target gene of miR-134-5p | |
| SYT13 | Target gene of miR-134-5p | |
| SYT14 | Target gene of miR-134-5p | |
| SYT15 | Target gene of miR-134-5p | |
| SYT16 | Target gene of miR-134-5p | |
| SYT2 | Target gene of miR-134-5p | |
| SYT3 | Target gene of miR-134-5p | |
| SYTL4 | Target gene of miR-134-5p | |
| TAB3 | Target gene of miR-134-5p | |
| TADA3 | Target gene of miR-134-5p | |
| TAF10 | Target gene of miR-134-5p | |
| TAF1C | Target gene of miR-134-5p | |
| TAF4B | Target gene of miR-134-5p | |
| TAF7L | Target gene of miR-134-5p | |
| TAFA5 | Target gene of miR-134-5p | |
| TANGO2 | Target gene of miR-134-5p | |
| TANGO6 | Target gene of miR-134-5p | |
| TAT | Target gene of miR-134-5p | |
| TBC1D1 | Target gene of miR-134-5p | |
| TBC1D14 | Target gene of miR-134-5p | |
| TBC1D20 | Target gene of miR-134-5p | |
| TBC1D26 | Target gene of miR-134-5p | |
| TBC1D5 | Target gene of miR-134-5p | |
| TBC1D7 | Target gene of miR-134-5p | |
| TCAF1 | Target gene of miR-134-5p | |
| TCF12 | Target gene of miR-134-5p | |
| TCF19 | Target gene of miR-134-5p | |
| TCF4 | Target gene of miR-134-5p | |
| TCFL5 | Target gene of miR-134-5p | |
| TCP11 | Target gene of miR-134-5p | |
| TCP11L1 | Target gene of miR-134-5p | |
| TCTA | Target gene of miR-134-5p | |
| TDRD15 | Target gene of miR-134-5p | |
| TDRKH | Target gene of miR-134-5p | |
| TENT5C | Target gene of miR-134-5p | |
| TEPSIN | Target gene of miR-134-5p | |
| TERF2 | Target gene of miR-134-5p | |
| TESPA1 | Target gene of miR-134-5p | |
| TEX2 | Target gene of miR-134-5p | |
| TEX46 | Target gene of miR-134-5p | |
| TFCP2L1 | Target gene of miR-134-5p | |
| TFDP2 | Target gene of miR-134-5p | |
| TGFBR2 | Target gene of miR-134-5p | |
| TGM2 | Target gene of miR-134-5p | |
| TGM4 | Target gene of miR-134-5p | |
| THOC3 | Target gene of miR-134-5p | |
| THRAP3 | Target gene of miR-134-5p | |
| THRSP | Target gene of miR-134-5p | |
| TIGIT | Target gene of miR-134-5p | |
| TIMP3 | Target gene of miR-134-5p | |
| TINAG | Target gene of miR-134-5p | |
| TK1 | Target gene of miR-134-5p | |
| TK2 | Target gene of miR-134-5p | |
| TLCD5 | Target gene of miR-134-5p | |
| TLK2 | Target gene of miR-134-5p | |
| TMBIM6 | Target gene of miR-134-5p | |
| TMED8 | Target gene of miR-134-5p | |
| TMEFF2 | Target gene of miR-134-5p | |
| TMEM106A | Target gene of miR-134-5p | |
| TMEM131L | Target gene of miR-134-5p | |
| TMEM144 | Target gene of miR-134-5p | |
| TMEM154 | Target gene of miR-134-5p | |
| TMEM178B | Target gene of miR-134-5p | |
| TMEM182 | Target gene of miR-134-5p | |
| TMEM192 | Target gene of miR-134-5p | |
| TMEM216 | Target gene of miR-134-5p | |
| TMEM233 | Target gene of miR-134-5p | |
| TMEM234 | Target gene of miR-134-5p | |
| TMEM245 | Target gene of miR-134-5p | |
| TMEM263 | Target gene of miR-134-5p | |
| TMEM269 | Target gene of miR-134-5p | |
| TMEM273 | Target gene of miR-134-5p | |
| TMEM275 | Target gene of miR-134-5p | |
| TMEM44 | Target gene of miR-134-5p | |
| TMEM52B | Target gene of miR-134-5p | |
| TMEM59 | Target gene of miR-134-5p | |
| TMEM8B | Target gene of miR-134-5p | |
| TMEM9 | Target gene of miR-134-5p | |
| TNFRSF10B | Target gene of miR-134-5p | |
| TNFRSF13C | Target gene of miR-134-5p | |
| TNFSF8 | Target gene of miR-134-5p | |
| TNFSF9 | Target gene of miR-134-5p | |
| TNMD | Target gene of miR-134-5p | |
| TNNI1 | Target gene of miR-134-5p | |
| TNR | Target gene of miR-134-5p | |
| TNRC6B | Target gene of miR-134-5p | |
| TOMM40L | Target gene of miR-134-5p | |
| TOMM6 | Target gene of miR-134-5p | |
| TOR1AIP2 | Target gene of miR-134-5p | |
| TOX4 | Target gene of miR-134-5p | |
| TP53I11 | Target gene of miR-134-5p | |
| TPD52L3 | Target gene of miR-134-5p | |
| TPH1 | Target gene of miR-134-5p | |
| TPM1 | Target gene of miR-134-5p | |
| TPM3 | Target gene of miR-134-5p | |
| TPTEP2-CSNK1E | Target gene of miR-134-5p | |
| TRAPPC10 | Target gene of miR-134-5p | |
| TRAPPC11 | Target gene of miR-134-5p | |
| TRAPPC5 | Target gene of miR-134-5p | |
| TRH | Target gene of miR-134-5p | |
| TRIM17 | Target gene of miR-134-5p | |
| TRIM22 | Target gene of miR-134-5p | |
| TRIM46 | Target gene of miR-134-5p | |
| TRIM48 | Target gene of miR-134-5p | |
| TRIM65 | Target gene of miR-134-5p | |
| TRIM67 | Target gene of miR-134-5p | |
| TRIML1 | Target gene of miR-134-5p | |
| TRMT61A | Target gene of miR-134-5p | |
| TSHZ2 | Target gene of miR-134-5p | |
| TSPAN18 | Target gene of miR-134-5p | |
| TSPAN31 | Target gene of miR-134-5p | |
| TSPEAR | Target gene of miR-134-5p | |
| TSPYL4 | Target gene of miR-134-5p | |
| TTC14 | Target gene of miR-134-5p | |
| TTC16 | Target gene of miR-134-5p | |
| TTC21B | Target gene of miR-134-5p | |
| TTC39B | Target gene of miR-134-5p | |
| TTC7B | Target gene of miR-134-5p | |
| TTC9 | Target gene of miR-134-5p | |
| TTLL11 | Target gene of miR-134-5p | |
| TULP1 | Target gene of miR-134-5p | |
| TUT7 | Target gene of miR-134-5p | |
| TXNDC8 | Target gene of miR-134-5p | |
| TXNL4A | Target gene of miR-134-5p | |
| TXNL4B | Target gene of miR-134-5p | |
| TYRO3 | Target gene of miR-134-5p | |
| U2AF2 | Target gene of miR-134-5p | |
| UBA52 | Target gene of miR-134-5p | |
| UBALD1 | Target gene of miR-134-5p | |
| UBE2L3 | Target gene of miR-134-5p | |
| UBE2Q2 | Target gene of miR-134-5p | |
| UBE2QL1 | Target gene of miR-134-5p | |
| UBIAD1 | Target gene of miR-134-5p | |
| UBP1 | Target gene of miR-134-5p | |
| UBTF | Target gene of miR-134-5p | |
| UBXN6 | Target gene of miR-134-5p | |
| UEVLD | Target gene of miR-134-5p | |
| UFSP2 | Target gene of miR-134-5p | |
| UHMK1 | Target gene of miR-134-5p | |
| ULK3 | Target gene of miR-134-5p | |
| UMAD1 | Target gene of miR-134-5p | |
| UQCC2 | Target gene of miR-134-5p | |
| UQCC3 | Target gene of miR-134-5p | |
| URI1 | Target gene of miR-134-5p | |
| USB1 | Target gene of miR-134-5p | |
| USF3 | Target gene of miR-134-5p | |
| USP12 | Target gene of miR-134-5p | |
| USP13 | Target gene of miR-134-5p | |
| USP17L2 | Target gene of miR-134-5p | |
| USP22 | Target gene of miR-134-5p | |
| USP33 | Target gene of miR-134-5p | |
| USP46 | Target gene of miR-134-5p | |
| USP49 | Target gene of miR-134-5p | |
| UVSSA | Target gene of miR-134-5p | |
| VAMP1 | Target gene of miR-134-5p | |
| VAMP7 | Target gene of miR-134-5p | |
| VANGL1 | Target gene of miR-134-5p | |
| VASP | Target gene of miR-134-5p | |
| VAX1 | Target gene of miR-134-5p | |
| VCL | Target gene of miR-134-5p | |
| VEZF1 | Target gene of miR-134-5p | |
| VIPAS39 | Target gene of miR-134-5p | |
| VIPR1 | Target gene of miR-134-5p | |
| VKORC1L1 | Target gene of miR-134-5p | |
| VPS29 | Target gene of miR-134-5p | |
| VPS37C | Target gene of miR-134-5p | |
| VPS39 | Target gene of miR-134-5p | |
| VPS4A | Target gene of miR-134-5p | |
| VPS52 | Target gene of miR-134-5p | |
| VPS53 | Target gene of miR-134-5p | |
| VSTM2A | Target gene of miR-134-5p | |
| VSTM5 | Target gene of miR-134-5p | |
| VTA1 | Target gene of miR-134-5p | |
| VWA5B1 | Target gene of miR-134-5p | |
| VWC2 | Target gene of miR-134-5p | |
| VXN | Target gene of miR-134-5p | |
| WAC | Target gene of miR-134-5p | |
| WDCP | Target gene of miR-134-5p | |
| WDR26 | Target gene of miR-134-5p | |
| WDR33 | Target gene of miR-134-5p | |
| WDR37 | Target gene of miR-134-5p | |
| WDR72 | Target gene of miR-134-5p | |
| WDR77 | Target gene of miR-134-5p | |
| WDR82 | Target gene of miR-134-5p | |
| WDR93 | Target gene of miR-134-5p | |
| WFDC13 | Target gene of miR-134-5p | |
| WFIKKN2 | Target gene of miR-134-5p | |
| WHRN | Target gene of miR-134-5p | |
| WNK3 | Target gene of miR-134-5p | |
| WSCD2 | Target gene of miR-134-5p | |
| WTIP | Target gene of miR-134-5p | |
| XIRP1 | Target gene of miR-134-5p | |
| XKR5 | Target gene of miR-134-5p | |
| XPNPEP3 | Target gene of miR-134-5p | |
| XPR1 | Target gene of miR-134-5p | |
| XRCC6 | Target gene of miR-134-5p | |
| YIPF6 | Target gene of miR-134-5p | |
| YME1L1 | Target gene of miR-134-5p | |
| YRDC | Target gene of miR-134-5p | |
| YWHAE | Target gene of miR-134-5p | |
| YWHAZ | Target gene of miR-134-5p | |
| ZBED3 | Target gene of miR-134-5p | |
| ZBTB10 | Target gene of miR-134-5p | |
| ZBTB26 | Target gene of miR-134-5p | |
| ZBTB34 | Target gene of miR-134-5p | |
| ZBTB37 | Target gene of miR-134-5p | |
| ZBTB40 | Target gene of miR-134-5p | |
| ZBTB8A | Target gene of miR-134-5p | |
| ZBTB8B | Target gene of miR-134-5p | |
| ZC2HC1A | Target gene of miR-134-5p | |
| ZC3H10 | Target gene of miR-134-5p | |
| ZC3H12B | Target gene of miR-134-5p | |
| ZC3H13 | Target gene of miR-134-5p | |
| ZC3H14 | Target gene of miR-134-5p | |
| ZC3H4 | Target gene of miR-134-5p | |
| ZC3H7B | Target gene of miR-134-5p | |
| ZCCHC24 | Target gene of miR-134-5p | |
| ZDHHC18 | Target gene of miR-134-5p | |
| ZDHHC2 | Target gene of miR-134-5p | |
| ZDHHC23 | Target gene of miR-134-5p | |
| ZDHHC24 | Target gene of miR-134-5p | |
| ZDHHC9 | Target gene of miR-134-5p | |
| ZFP2 | Target gene of miR-134-5p | |
| ZFP36L1 | Target gene of miR-134-5p | |
| ZFYVE1 | Target gene of miR-134-5p | |
| ZHX3 | Target gene of miR-134-5p | |
| ZKSCAN1 | Target gene of miR-134-5p | |
| ZKSCAN2 | Target gene of miR-134-5p | |
| ZMIZ2 | Target gene of miR-134-5p | |
| ZMYND8 | Target gene of miR-134-5p | |
| ZNF106 | Target gene of miR-134-5p | |
| ZNF112 | Target gene of miR-134-5p | |
| ZNF135 | Target gene of miR-134-5p | |
| ZNF142 | Target gene of miR-134-5p | |
| ZNF148 | Target gene of miR-134-5p | |
| ZNF169 | Target gene of miR-134-5p | |
| ZNF202 | Target gene of miR-134-5p | |
| ZNF214 | Target gene of miR-134-5p | |
| ZNF233 | Target gene of miR-134-5p | |
| ZNF264 | Target gene of miR-134-5p | |
| ZNF268 | Target gene of miR-134-5p | |
| ZNF280B | Target gene of miR-134-5p | |
| ZNF3 | Target gene of miR-134-5p | |
| ZNF302 | Target gene of miR-134-5p | |
| ZNF304 | Target gene of miR-134-5p | |
| ZNF320 | Target gene of miR-134-5p | |
| ZNF33A | Target gene of miR-134-5p | |
| ZNF346 | Target gene of miR-134-5p | |
| ZNF365 | Target gene of miR-134-5p | |
| ZNF385A | Target gene of miR-134-5p | |
| ZNF398 | Target gene of miR-134-5p | |
| ZNF407 | Target gene of miR-134-5p | |
| ZNF423 | Target gene of miR-134-5p | |
| ZNF440 | Target gene of miR-134-5p | |
| ZNF445 | Target gene of miR-134-5p | |
| ZNF449 | Target gene of miR-134-5p | |
| ZNF451 | Target gene of miR-134-5p | |
| ZNF471 | Target gene of miR-134-5p | |
| ZNF473 | Target gene of miR-134-5p | |
| ZNF507 | Target gene of miR-134-5p | |
| ZNF512 | Target gene of miR-134-5p | |
| ZNF517 | Target gene of miR-134-5p | |
| ZNF518B | Target gene of miR-134-5p | |
| ZNF534 | Target gene of miR-134-5p | |
| ZNF544 | Target gene of miR-134-5p | |
| ZNF556 | Target gene of miR-134-5p | |
| ZNF559-ZNF177 | Target gene of miR-134-5p | |
| ZNF561 | Target gene of miR-134-5p | |
| ZNF562 | Target gene of miR-134-5p | |
| ZNF577 | Target gene of miR-134-5p | |
| ZNF606 | Target gene of miR-134-5p | |
| ZNF611 | Target gene of miR-134-5p | |
| ZNF615 | Target gene of miR-134-5p | |
| ZNF619 | Target gene of miR-134-5p | |
| ZNF621 | Target gene of miR-134-5p | |
| ZNF655 | Target gene of miR-134-5p | |
| ZNF660 | Target gene of miR-134-5p | |
| ZNF662 | Target gene of miR-134-5p | |
| ZNF678 | Target gene of miR-134-5p | |
| ZNF680 | Target gene of miR-134-5p | |
| ZNF70 | Target gene of miR-134-5p | |
| ZNF711 | Target gene of miR-134-5p | |
| ZNF717 | Target gene of miR-134-5p | |
| ZNF723 | Target gene of miR-134-5p | |
| ZNF726 | Target gene of miR-134-5p | |
| ZNF74 | Target gene of miR-134-5p | |
| ZNF749 | Target gene of miR-134-5p | |
| ZNF75A | Target gene of miR-134-5p | |
| ZNF778 | Target gene of miR-134-5p | |
| ZNF84 | Target gene of miR-134-5p | |
| ZNF844 | Target gene of miR-134-5p | |
| ZNF850 | Target gene of miR-134-5p | |
| ZNRF1 | Target gene of miR-134-5p | |
| ZSCAN20 | Target gene of miR-134-5p | |
| ZSCAN22 | Target gene of miR-134-5p | |
| ZSWIM4 | Target gene of miR-134-5p | |
| ZYG11A | Target gene of miR-134-5p | |

**Table S6: Functional annotation (KEGG pathways) of the 7 candidate target genes of miR-134-5p.**

| ID | Description | GeneRatio | BgRatio | pvalue | p.adjust | qvalue | geneID | Count |
| --- | --- | --- | --- | --- | --- | --- | --- | --- |
| hsa05205 | Proteoglycans in cancer | 5/6 | 205/8158 | 5.61E-08 | 4.10E-06 | 2.72E-06 | MMP2/PTK2/WNT5A/VEGFA/ITGB1 | 5 |
| hsa05165 | Human papillomavirus infection | 5/6 | 331/8158 | 6.19E-07 | 2.26E-05 | 1.50E-05 | PTK2/WNT5A/VEGFA/PDGFRB/ITGB1 | 5 |
| hsa04510 | Focal adhesion | 4/6 | 201/8158 | 5.16E-06 | 0.000125625 | 8.33E-05 | PTK2/VEGFA/PDGFRB/ITGB1 | 4 |
| hsa04151 | PI3K-Akt signaling pathway | 4/6 | 354/8158 | 4.88E-05 | 0.000752717 | 0.000499279 | PTK2/VEGFA/PDGFRB/ITGB1 | 4 |
| hsa04670 | Leukocyte transendothelial migration | 3/6 | 114/8158 | 5.16E-05 | 0.000752717 | 0.000499279 | MMP2/PTK2/ITGB1 | 3 |
| hsa05418 | Fluid shear stress and atherosclerosis | 3/6 | 139/8158 | 9.33E-05 | 0.001134588 | 0.000752575 | MMP2/PTK2/VEGFA | 3 |
| hsa04360 | Axon guidance | 3/6 | 182/8158 | 0.000207901 | 0.002168109 | 0.001438111 | PTK2/WNT5A/ITGB1 | 3 |
| hsa04015 | Rap1 signaling pathway | 3/6 | 210/8158 | 0.000317581 | 0.002664402 | 0.001767303 | VEGFA/PDGFRB/ITGB1 | 3 |
| hsa04810 | Regulation of actin cytoskeleton | 3/6 | 218/8158 | 0.000354667 | 0.002664402 | 0.001767303 | PTK2/PDGFRB/ITGB1 | 3 |
| hsa05219 | Bladder cancer | 2/6 | 41/8158 | 0.000364987 | 0.002664402 | 0.001767303 | MMP2/VEGFA | 2 |
| hsa04370 | VEGF signaling pathway | 2/6 | 59/8158 | 0.000757095 | 0.005024358 | 0.003332667 | PTK2/VEGFA | 2 |
| hsa05100 | Bacterial invasion of epithelial cells | 2/6 | 77/8158 | 0.001287093 | 0.007605392 | 0.005044673 | PTK2/ITGB1 | 2 |
| hsa01521 | EGFR tyrosine kinase inhibitor resistance | 2/6 | 79/8158 | 0.001354385 | 0.007605392 | 0.005044673 | VEGFA/PDGFRB | 2 |
| hsa05222 | Small cell lung cancer | 2/6 | 92/8158 | 0.001832296 | 0.009554114 | 0.006337264 | PTK2/ITGB1 | 2 |
| hsa01522 | Endocrine resistance | 2/6 | 98/8158 | 0.002076386 | 0.009859681 | 0.006539947 | MMP2/PTK2 | 2 |
| hsa04933 | AGE-RAGE signaling pathway in diabetic complications | 2/6 | 100/8158 | 0.002161026 | 0.009859681 | 0.006539947 | MMP2/VEGFA | 2 |
| hsa04926 | Relaxin signaling pathway | 2/6 | 129/8158 | 0.003570117 | 0.015330502 | 0.010168754 | MMP2/VEGFA | 2 |
| hsa05135 | Yersinia infection | 2/6 | 137/8158 | 0.004017895 | 0.016294795 | 0.010808371 | PTK2/ITGB1 | 2 |
| hsa05208 | Chemical carcinogenesis - reactive oxygen species | 2/6 | 223/8158 | 0.010377031 | 0.038534651 | 0.025560115 | PTK2/VEGFA | 2 |
| hsa05163 | Human cytomegalovirus infection | 2/6 | 225/8158 | 0.010557439 | 0.038534651 | 0.025560115 | PTK2/VEGFA | 2 |
| hsa04014 | Ras signaling pathway | 2/6 | 235/8158 | 0.01148088 | 0.039671827 | 0.026314406 | VEGFA/PDGFRB | 2 |
| hsa04020 | Calcium signaling pathway | 2/6 | 240/8158 | 0.011955893 | 0.039671827 | 0.026314406 | VEGFA/PDGFRB | 2 |
| hsa05131 | Shigellosis | 2/6 | 247/8158 | 0.012635681 | 0.040104553 | 0.026601434 | PTK2/ITGB1 | 2 |
| hsa04010 | MAPK signaling pathway | 2/6 | 294/8158 | 0.017636647 | 0.053644801 | 0.035582708 | VEGFA/PDGFRB | 2 |
| hsa05206 | MicroRNAs in cancer | 2/6 | 310/8158 | 0.019508078 | 0.056963589 | 0.037784067 | VEGFA/PDGFRB | 2 |

**Table S7: Functional annotation (Top ten of Go terms) of the 7 candidate target genes of miR-134-5p.**

| ONTOLOGY | ID | Description | GeneRatio | BgRatio | pvalue | p.adjust | qvalue | geneID | Count |
| --- | --- | --- | --- | --- | --- | --- | --- | --- | --- |
| BP | GO:0042060 | wound healing | 6/7 | 422/18723 | 8.69E-10 | 1.00E-06 | 2.32E-07 | LOX/PTK2/WNT5A/VEGFA/PDGFRB/ITGB1 | 6 |
| BP | GO:0060326 | cell chemotaxis | 5/7 | 310/18723 | 2.46E-08 | 1.42E-05 | 3.28E-06 | LOX/PTK2/WNT5A/VEGFA/PDGFRB | 5 |
| BP | GO:0014706 | striated muscle tissue development | 5/7 | 384/18723 | 7.18E-08 | 2.44E-05 | 5.65E-06 | LOX/WNT5A/VEGFA/PDGFRB/ITGB1 | 5 |
| BP | GO:0060537 | muscle tissue development | 5/7 | 403/18723 | 9.14E-08 | 2.44E-05 | 5.65E-06 | LOX/WNT5A/VEGFA/PDGFRB/ITGB1 | 5 |
| BP | GO:0050921 | positive regulation of chemotaxis | 4/7 | 141/18723 | 1.06E-07 | 2.44E-05 | 5.65E-06 | PTK2/WNT5A/VEGFA/PDGFRB | 4 |
| BP | GO:0030324 | lung development | 4/7 | 177/18723 | 2.64E-07 | 4.44E-05 | 1.03E-05 | LOX/WNT5A/VEGFA/PDGFRB | 4 |
| BP | GO:0030323 | respiratory tube development | 4/7 | 181/18723 | 2.89E-07 | 4.44E-05 | 1.03E-05 | LOX/WNT5A/VEGFA/PDGFRB | 4 |
| BP | GO:0055001 | muscle cell development | 4/7 | 184/18723 | 3.09E-07 | 4.44E-05 | 1.03E-05 | LOX/VEGFA/PDGFRB/ITGB1 | 4 |
| BP | GO:0060541 | respiratory system development | 4/7 | 203/18723 | 4.58E-07 | 5.85E-05 | 1.35E-05 | LOX/WNT5A/VEGFA/PDGFRB | 4 |
| BP | GO:0050920 | regulation of chemotaxis | 4/7 | 223/18723 | 6.67E-07 | 7.67E-05 | 1.78E-05 | PTK2/WNT5A/VEGFA/PDGFRB | 4 |
| CC | GO:0005925 | focal adhesion | 3/7 | 418/19550 | 0.00031861 | 0.011876957 | 0.006339073 | PTK2/PDGFRB/ITGB1 | 3 |
| CC | GO:0030055 | cell-substrate junction | 3/7 | 425/19550 | 0.000334562 | 0.011876957 | 0.006339073 | PTK2/PDGFRB/ITGB1 | 3 |
| CC | GO:0043197 | dendritic spine | 2/7 | 183/19550 | 0.001774372 | 0.03183576 | 0.016991658 | PTK2/ITGB1 | 2 |
| CC | GO:0044309 | neuron spine | 2/7 | 184/19550 | 0.001793564 | 0.03183576 | 0.016991658 | PTK2/ITGB1 | 2 |
| CC | GO:0098978 | glutamatergic synapse | 2/7 | 338/19550 | 0.005909344 | 0.082590557 | 0.044080949 | WNT5A/ITGB1 | 2 |
| CC | GO:0062023 | collagen-containing extracellular matrix | 2/7 | 425/19550 | 0.009210074 | 0.082590557 | 0.044080949 | MMP2/WNT5A | 2 |
| CC | GO:0008305 | integrin complex | 5/7 | 31/19550 | 0.011048769 | 0.082590557 | 0.044080949 | ITGB1 | 1 |
| CC | GO:0097386 | glial cell projection | 5/7 | 33/19550 | 0.011757985 | 0.082590557 | 0.044080949 | ITGB1 | 1 |
| CC | GO:0005911 | cell-cell junction | 2/7 | 494/19550 | 0.012300788 | 0.082590557 | 0.044080949 | VEGFA/ITGB1 | 2 |
| CC | GO:0098636 | protein complex involved in cell adhesion | 4/7 | 36/19550 | 0.012820992 | 0.082590557 | 0.044080949 | ITGB1 | 1 |
| MF | GO:0005161 | platelet-derived growth factor receptor binding | 2/7 | 15/18368 | 1.30E-05 | 0.000652054 | 0.000260822 | VEGFA/PDGFRB | 2 |
| MF | GO:0001968 | fibronectin binding | 2/7 | 28/18368 | 4.68E-05 | 0.00117093 | 0.000468372 | VEGFA/ITGB1 | 2 |
| MF | GO:0042056 | chemoattractant activity | 2/7 | 37/18368 | 8.24E-05 | 0.001373132 | 0.000549253 | WNT5A/VEGFA | 2 |
| MF | GO:0050840 | extracellular matrix binding | 2/7 | 56/18368 | 0.00018985 | 0.002373128 | 0.000949251 | VEGFA/ITGB1 | 2 |
| MF | GO:0005518 | collagen binding | 2/7 | 69/18368 | 0.000288531 | 0.00288531 | 0.001154124 | LOX/ITGB1 | 2 |
| MF | GO:0004714 | transmembrane receptor protein tyrosine kinase activity | 2/7 | 124/18368 | 0.000928578 | 0.00624568 | 0.002498272 | PTK2/PDGFRB | 2 |
| MF | GO:0004713 | protein tyrosine kinase activity | 2/7 | 136/18368 | 0.001115363 | 0.00624568 | 0.002498272 | PTK2/PDGFRB | 2 |
| MF | GO:0070851 | growth factor receptor binding | 2/7 | 141/18368 | 0.001198106 | 0.00624568 | 0.002498272 | VEGFA/PDGFRB | 2 |
| MF | GO:0019199 | transmembrane receptor protein kinase activity | 2/7 | 143/18368 | 0.001232011 | 0.00624568 | 0.002498272 | PTK2/PDGFRB | 2 |
| MF | GO:0005178 | integrin binding | 2/7 | 144/18368 | 0.001249136 | 0.00624568 | 0.002498272 | PTK2/ITGB1 | 2 |
