# Supplementary material for: MiR-134-5p inhibits the malignant phenotypes of osteosarcoma via ITGB1/MMP2/PI3K/Akt pathway
Source: Cell Death Discov. 2024 Apr 25;10:193. doi: 10.1038/s41420-024-01946-z (PMC11045734; doi:10.1038/s41420-024-01946-z)
Supplement: Supplementary file 2 — Supplemental Figures legends [file 41420_2024_1946_MOESM2_ESM.docx]

**Supplementary Figure Legends**

**Fig. S1** Knockdown of miR-134-5p promotes the VM, proliferation, migration and invasion of OS. (A) miR-134-5p mRNA in transfected cells, shown by qRT-PCR. (B) Tube formation assays to evaluate the effects of the miR-134-5p mimic on VM. Scale bar, 200 μm. (C-E) Colony formation, CCK-8, and EdU assays (scale bar, 400 μm) to evaluate the effects of miR-134-5p knockdown on proliferation. (F) Transwell assays to evaluate the effects of miR-134-5p knockdown on migration and invasion. (G) Wound-healing assays to evaluate the effects of miR-134-5p knockdown on migration. Statistics using one-way ANOVA with Tukey's test. The data represent means ± SD. *p < 0.05; **p < 0.01; ***p < 0.001.

**Fig. S2** Bioinformatics analysis of the characteristics of seven candidate target genes of miR-134-5p. (A) Chromosomal localization of the seven candidate target genes. (B) PPI network of seven candidate target genes. (C) ROC curve analysis of seven candidate target genes. (D) GO analysis of the seven candidate target genes. (E) Correlations between the seven candidate target genes. (F, G) The groups were divided by the median expression of MMP2 or ITGB1. Uniquely colored lines represent one gene set; upregulated genes are shown on the left and downregulated genes on the right. Significance was defined by NOM p < 0.05 and FDR q < 0.25. Only the top four gene sets are illustrated.

**Fig. S3** Gain-of-function analyses verifying the involvement of the miR-134-5p/ITGB1/MMP2 axis in OS VM, proliferation, migration, and invasion. (A) Tube formation assays to evaluate the effects of the miR-134-5p/ITGB1/MMP2 axis on VM. Scale bar, 200 μm. (B) CCK-8 assay to evaluate the effects of the miR-134-5p/ITGB1/MMP2 axis on cell proliferation. (C) Transwell assays to evaluate the effects of the miR-134-5p/ITGB1/MMP2 axis on migration and invasion. Scale bar, 100 μm. (D) Protein levels of p-PI3K, PI3K, p-Akt, and Akt, shown by western blotting. Statistics using one-way ANOVA with Tukey's test. The data represent means ± SD. *p < 0.05; **p < 0.01; ***p < 0.001.

**Fig. S4** Toxicity observation. H&E staining of heart, liver, spleen, lung and kidney.
