# Supplementary figures and images for: MiR-134-5p inhibits the malignant phenotypes of osteosarcoma via ITGB1/MMP2/PI3K/Akt pathway
Source: Cell Death Discov. 2024 Apr 25;10:193. doi: 10.1038/s41420-024-01946-z (PMC11045734; doi:10.1038/s41420-024-01946-z)

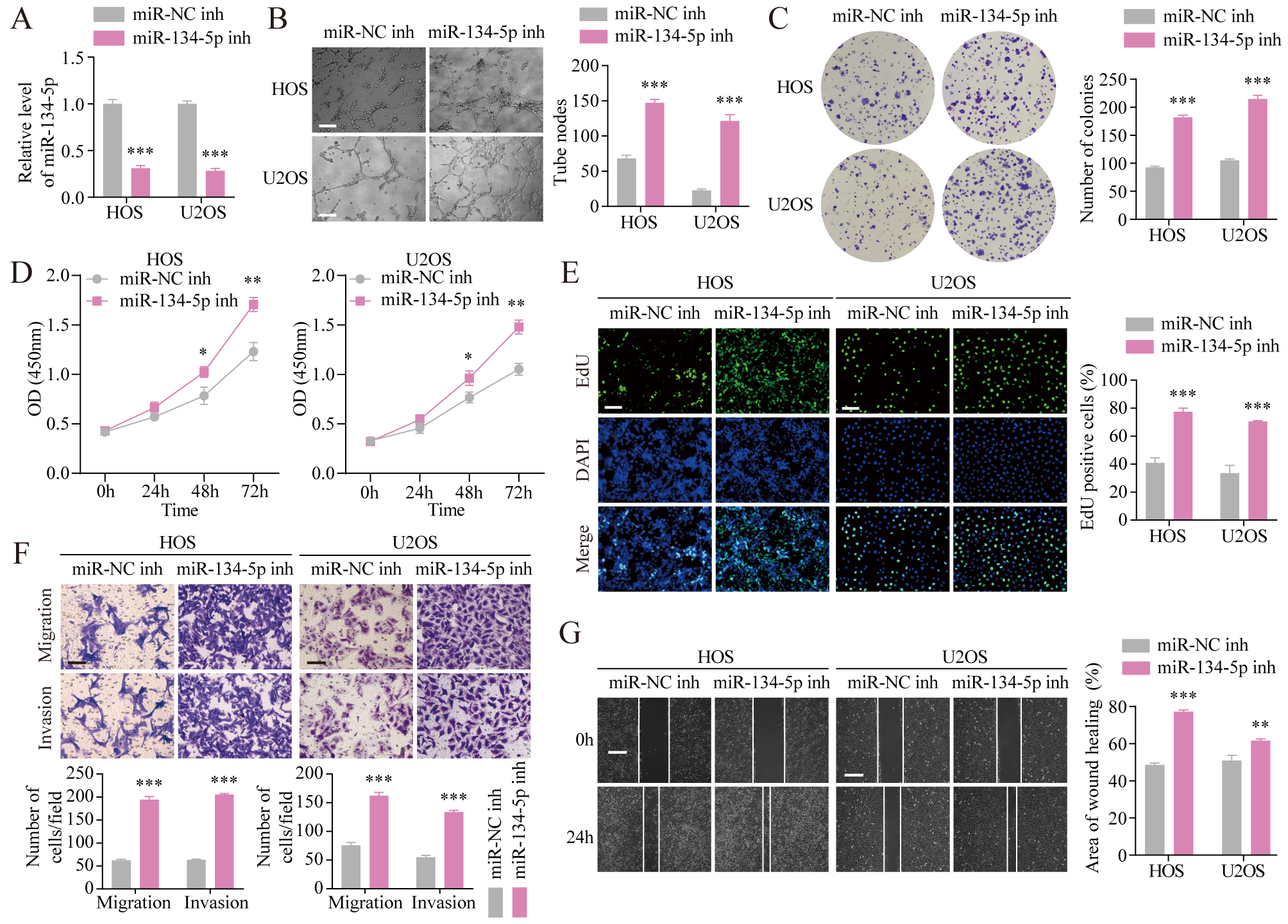

Supplement: Supplementary file 3 — Fig. S1 Knockdown of miR-134-5p promotes the VM, proliferation, migration and invasion of OS. [file 41420_2024_1946_MOESM3_ESM.tif]

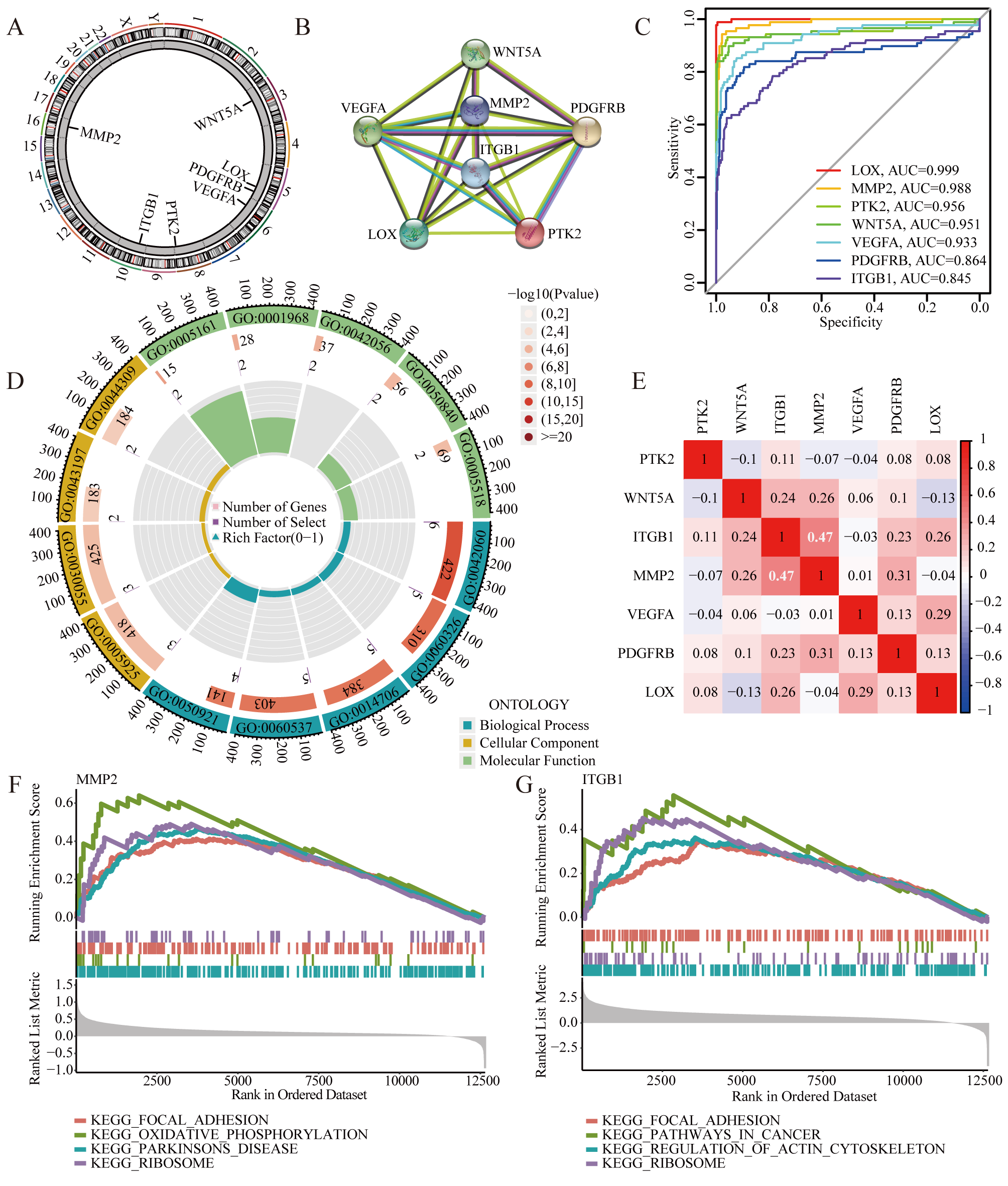

Supplement: Supplementary file 4 — Fig. S2 Bioinformatics analysis of the characteristics of seven candidate target genes of miR-134-5p. [file 41420_2024_1946_MOESM4_ESM.tif]

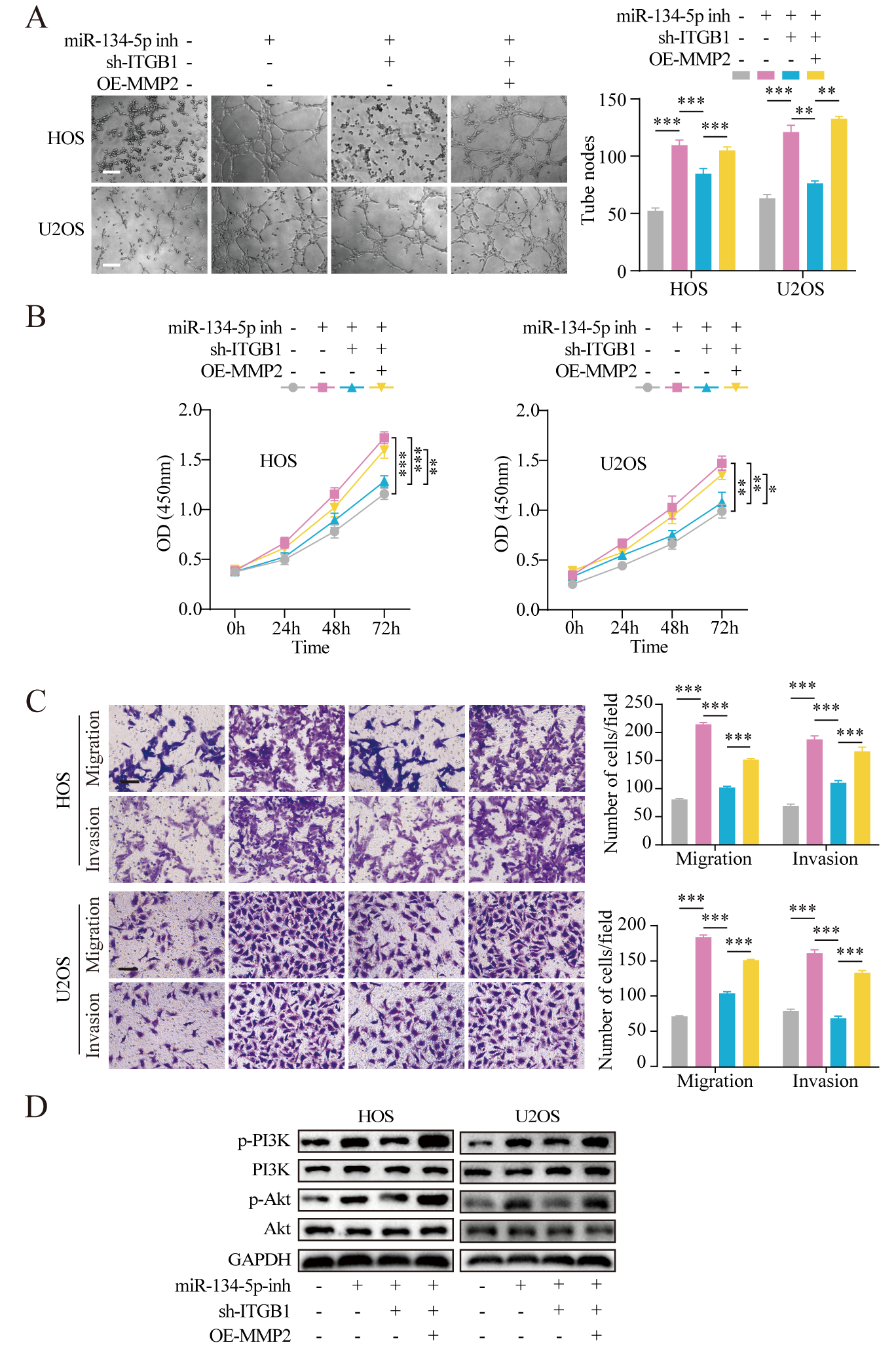

Supplement: Supplementary file 5 — Fig. S3 Gain-of-function analyses verifying the involvement of the miR-134-5p/ITGB1/MMP2 axis in OS VM, proliferation, migration, and invasion. [file 41420_2024_1946_MOESM5_ESM.tif]

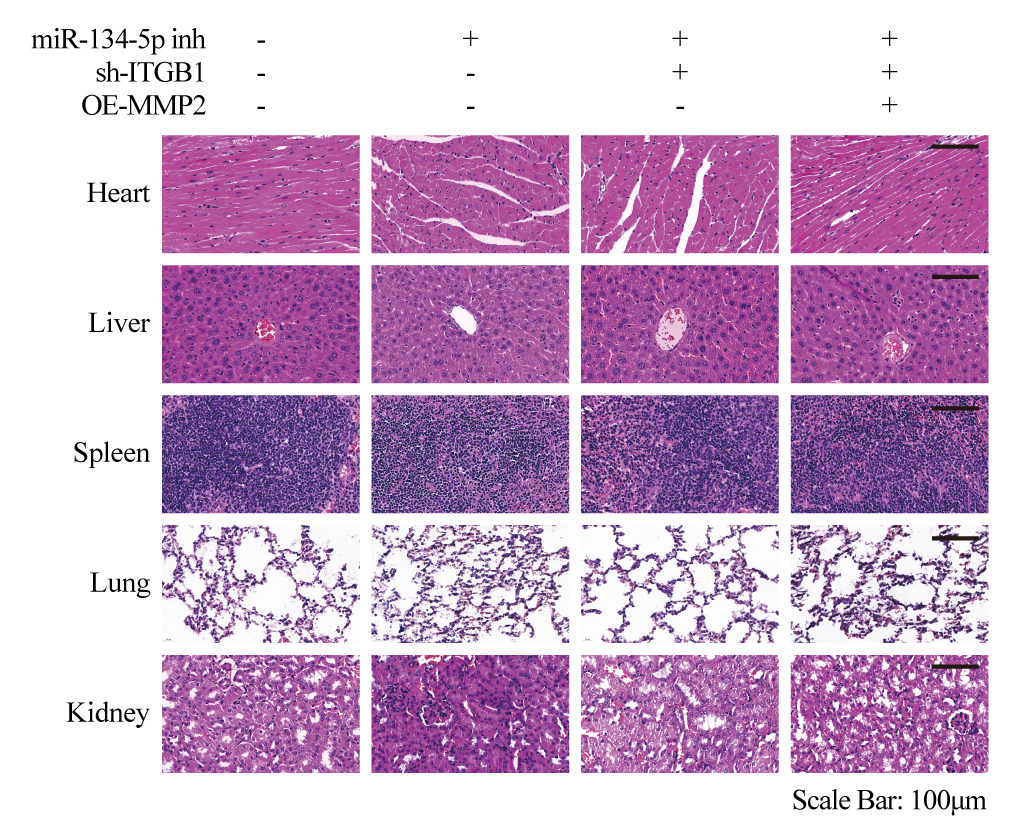

Supplement: Supplementary file 6 — Fig. S4 Toxicity observation. [file 41420_2024_1946_MOESM6_ESM.tif]
