# Supplementary material for: MiR-134-5p inhibits the malignant phenotypes of osteosarcoma via ITGB1/MMP2/PI3K/Akt pathway
Source: Cell Death Discov. 2024 Apr 25;10:193. doi: 10.1038/s41420-024-01946-z (PMC11045734; doi:10.1038/s41420-024-01946-z)

AKT-figure 2D-HOS


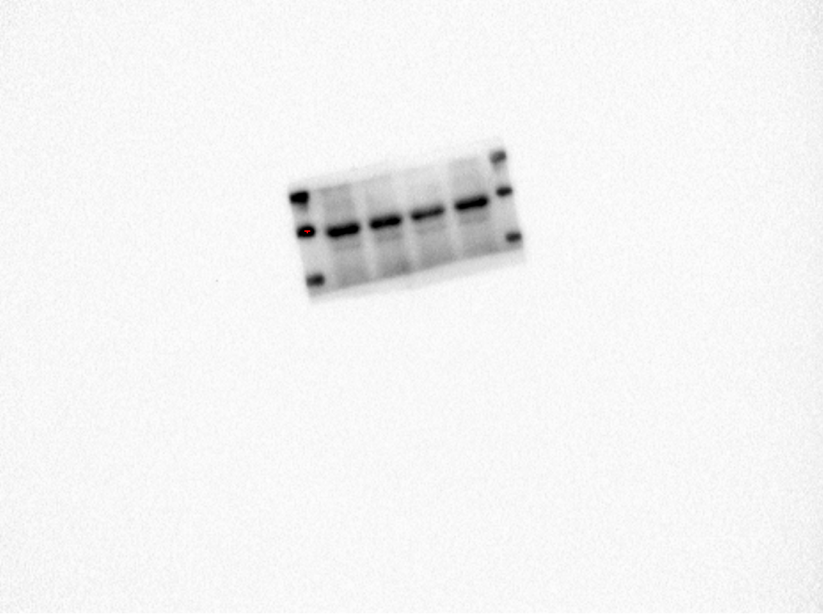


AKT-figure 2D-U2OS


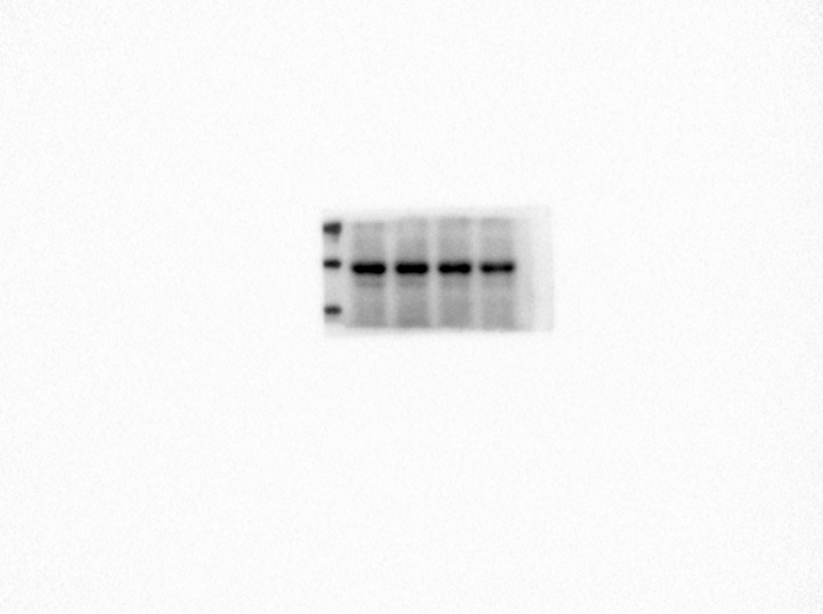


AKT-figure 4D-HOS


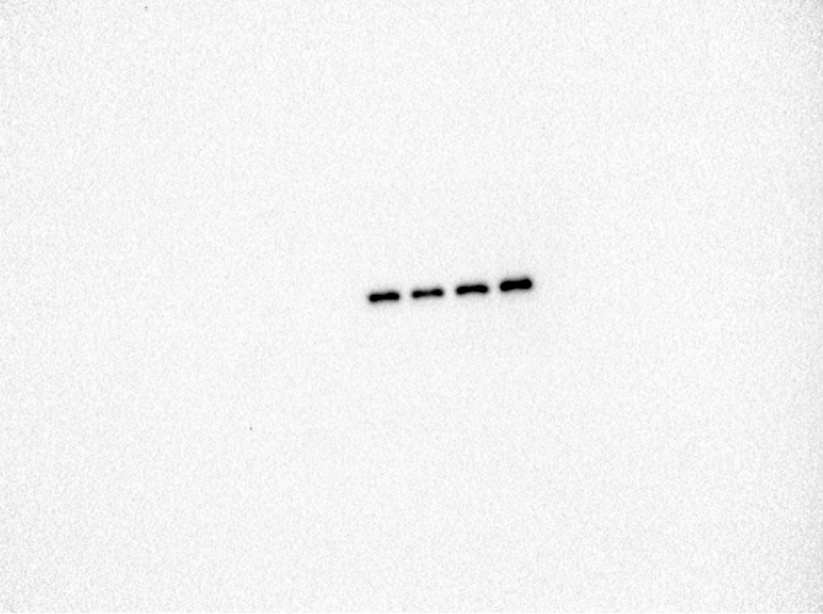


AKT-figure 4D-U2OS


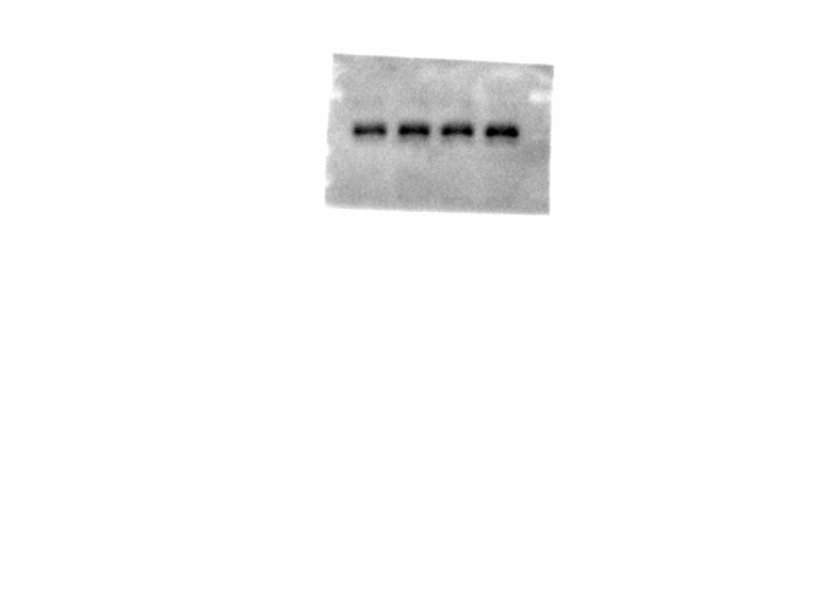


AKT-figure 5A-HOS


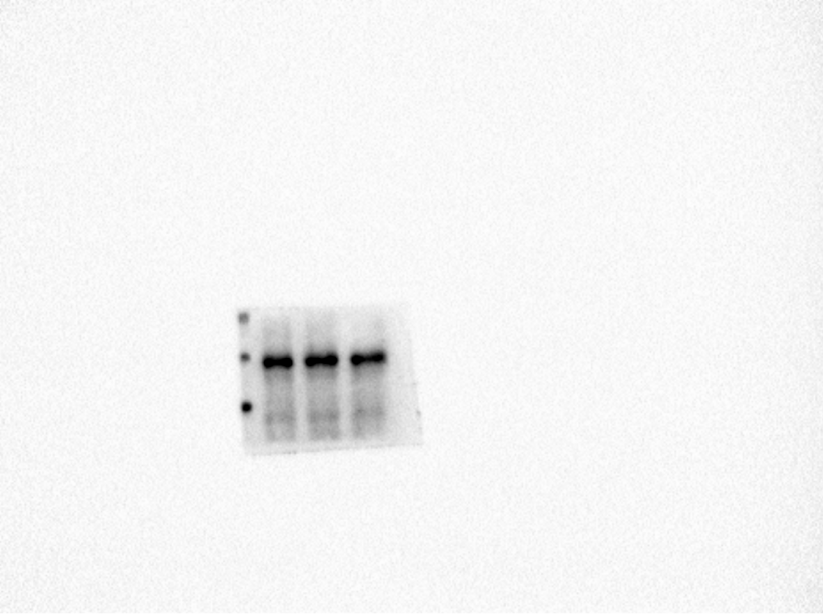


AKT-figure 5A-U2OS


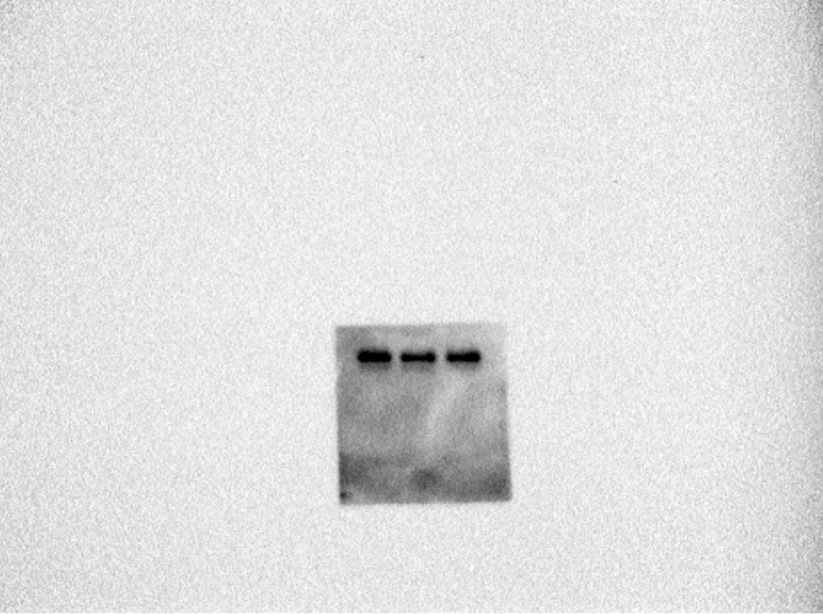


AKT-figure S3G-HOS


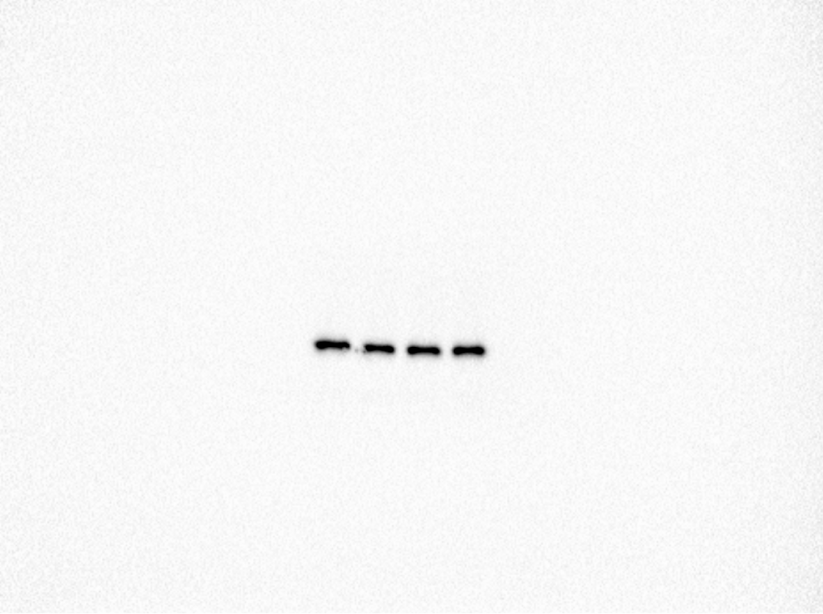


AKT-figure S3G-U2OS


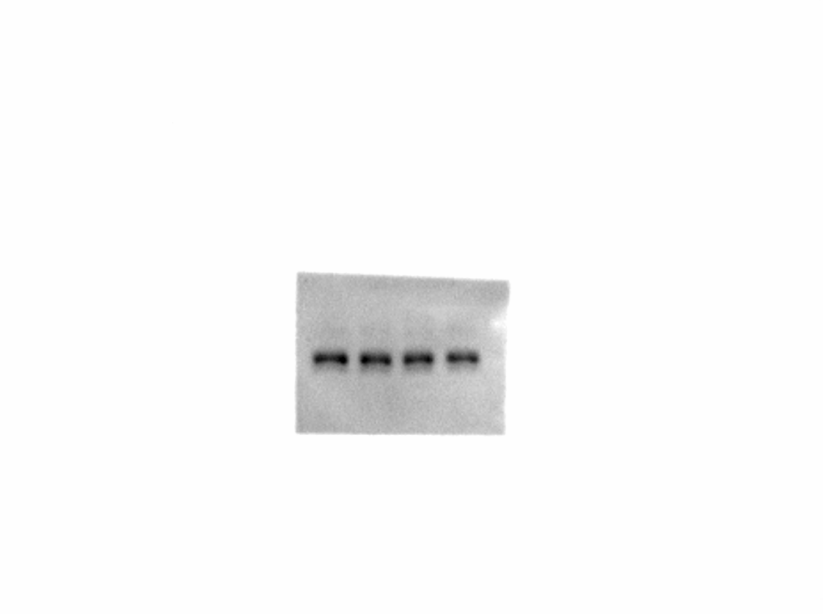


GAPDH-figure 2D-HOS


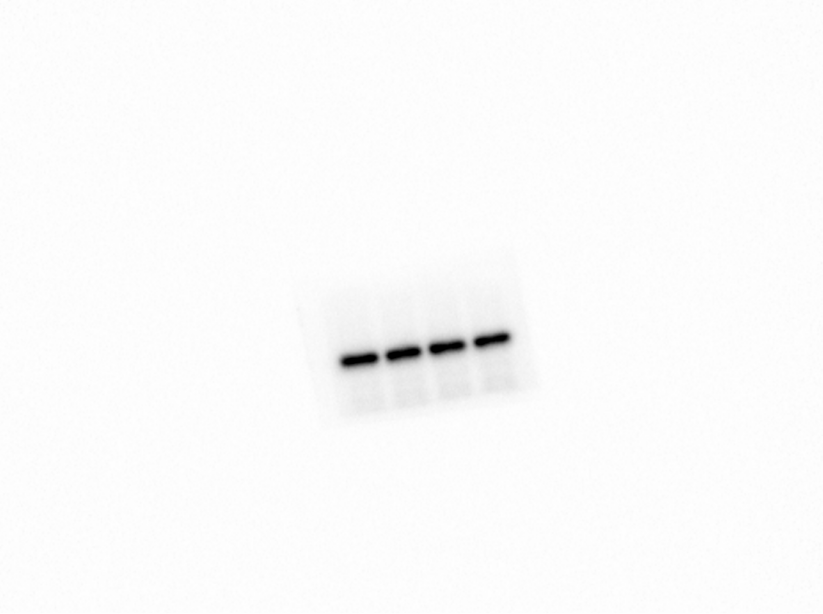


GAPDH-figure 2D-U2OS


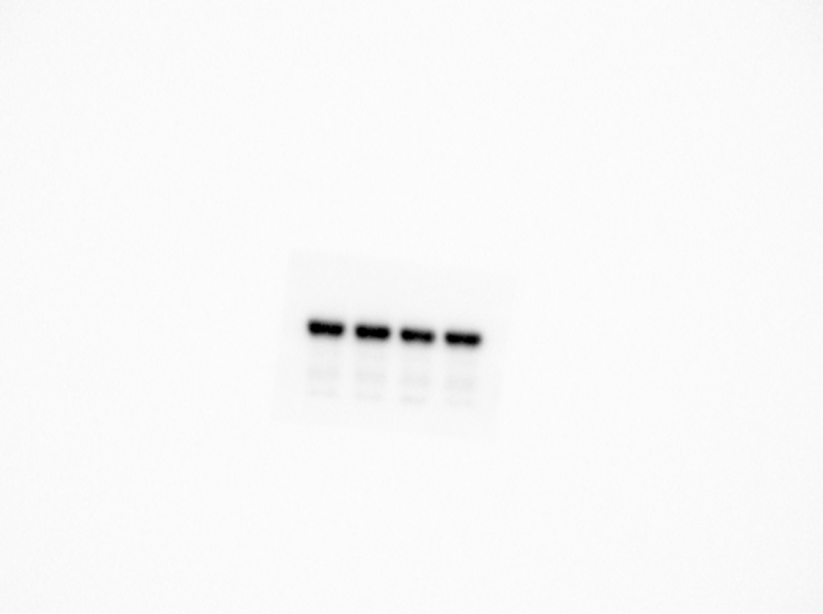


GAPDH-figure 2G-ITGB1


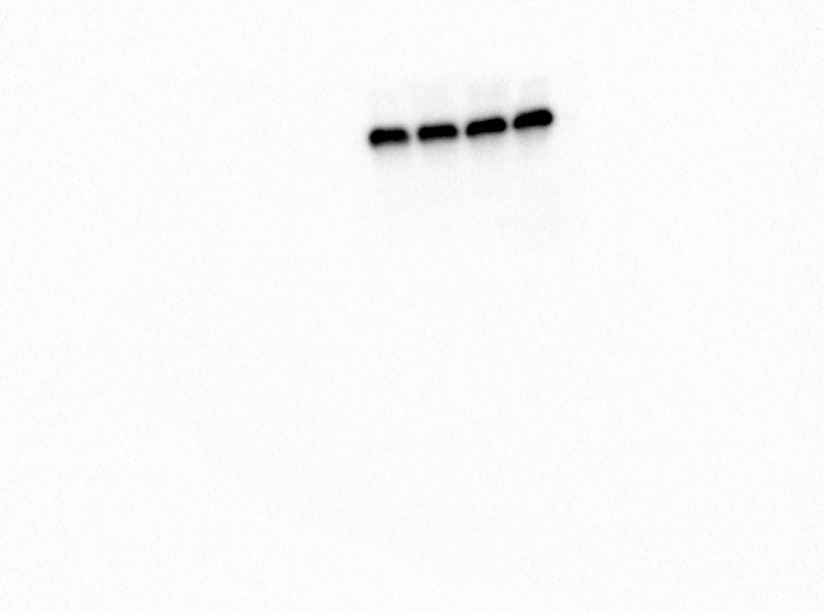


GAPDH-figure 2G-MMP2


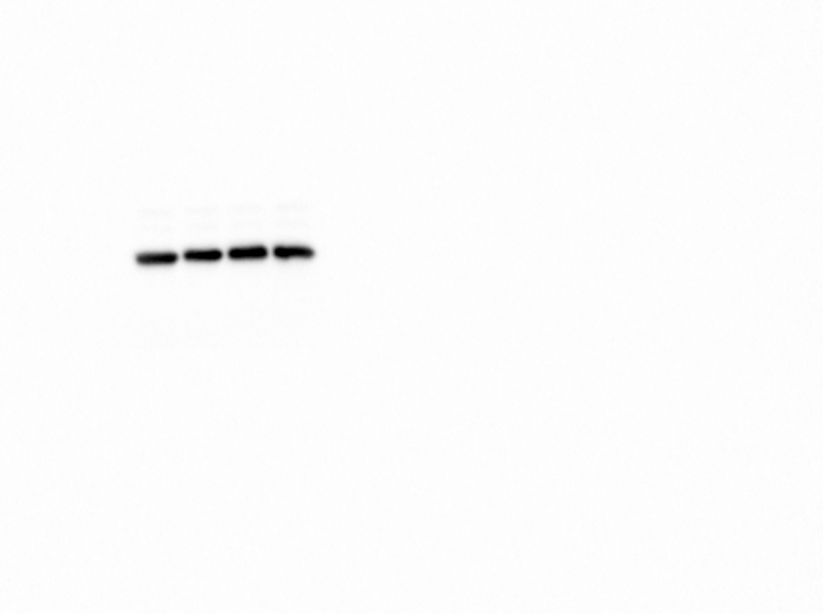


GAPDH-figure 2H-ITGB1


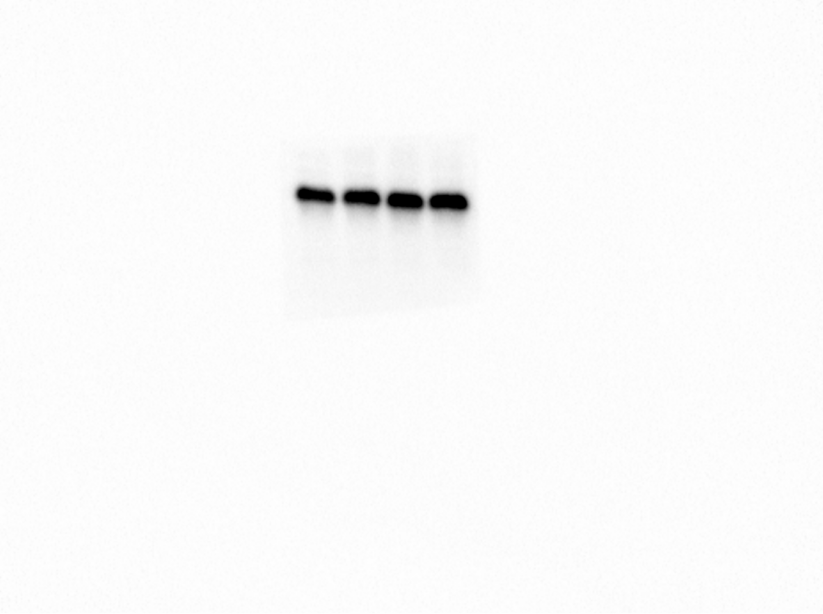


GAPDH-figure 2H-MMP2


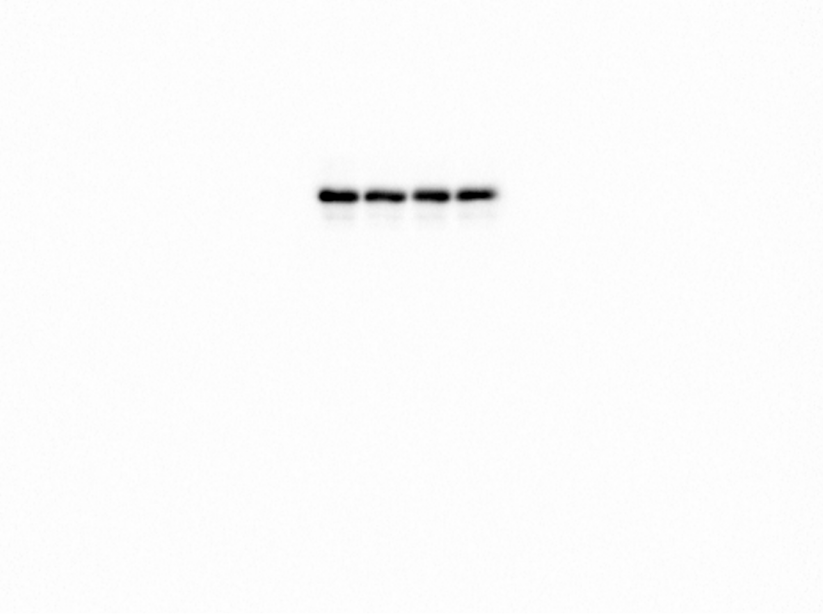


GAPDH-figure 2I-ITGB1


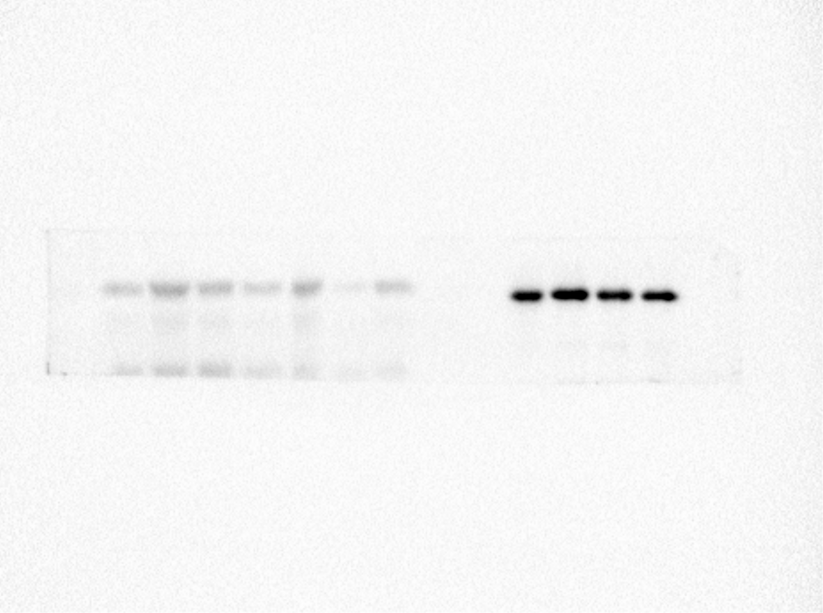


GAPDH-figure 2I-MMP2


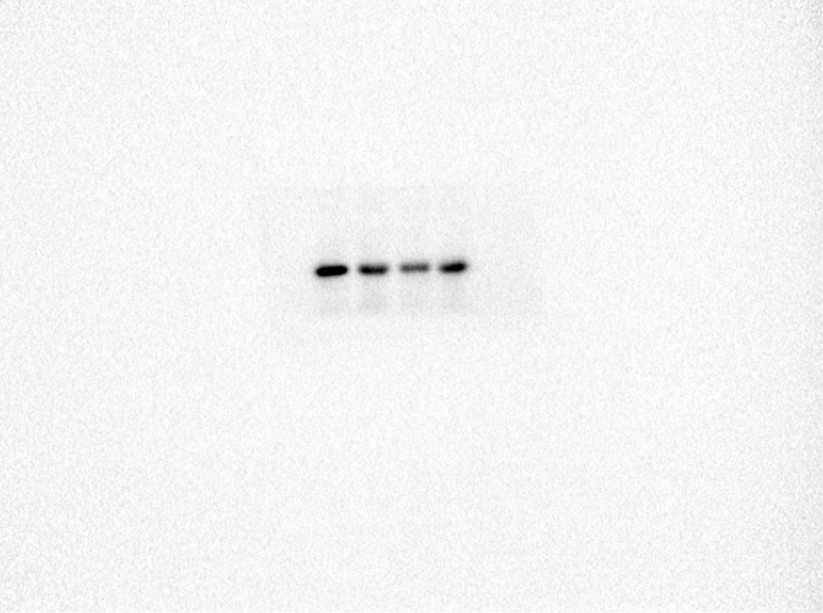


GAPDH-figure 2K


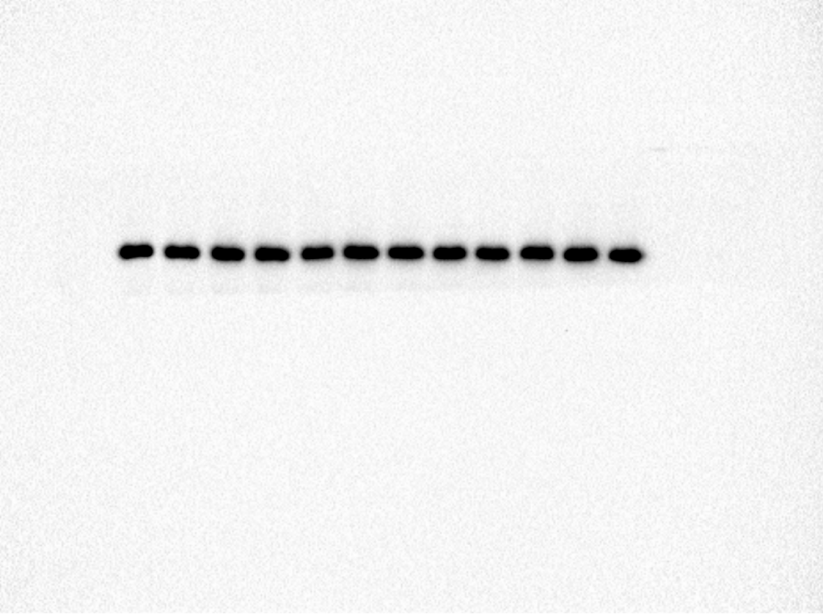


GAPDH-figure 3A-HOS


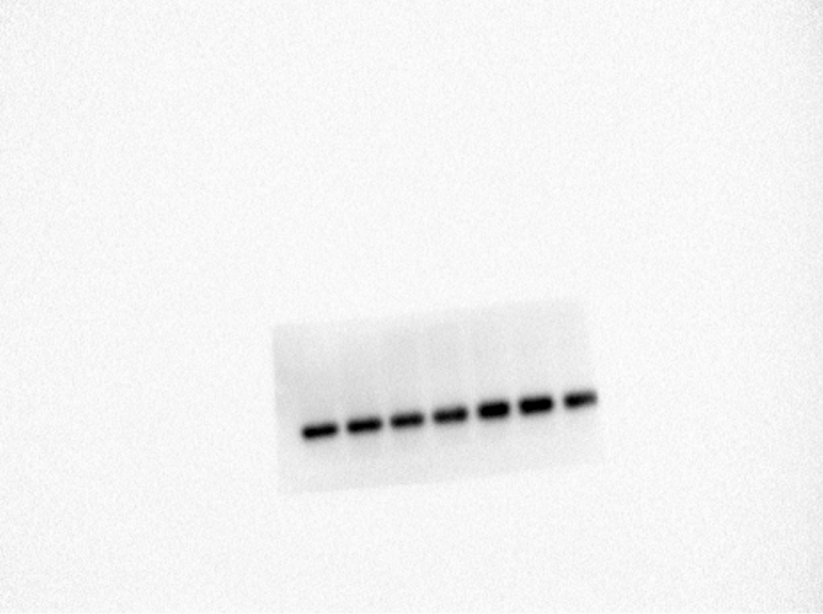


GAPDH-figure 3A-U2OS


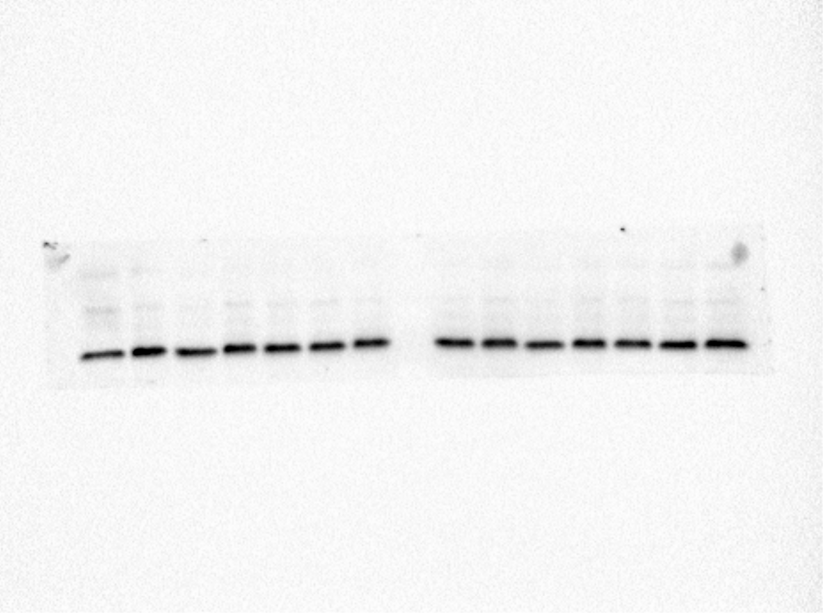


GAPDH-figure 3B-HOS


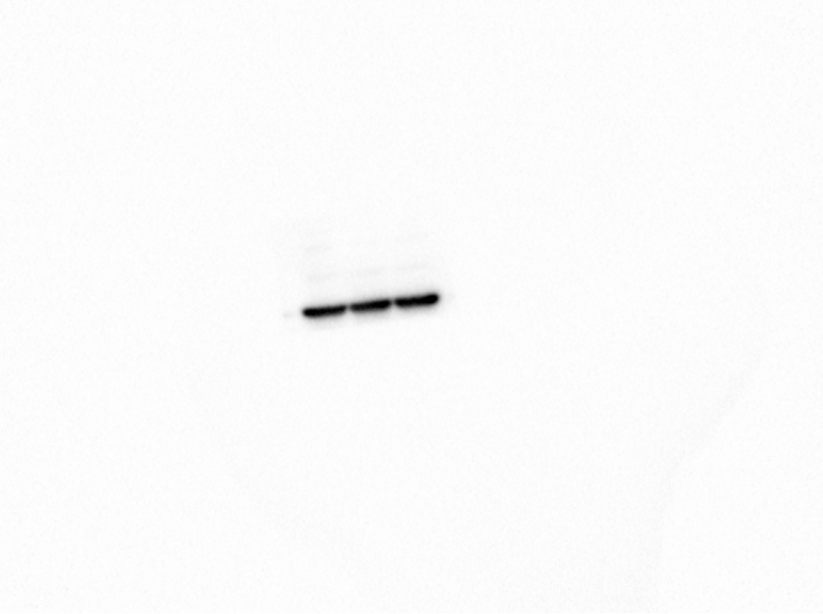


GAPDH-figure 3B-U2OS


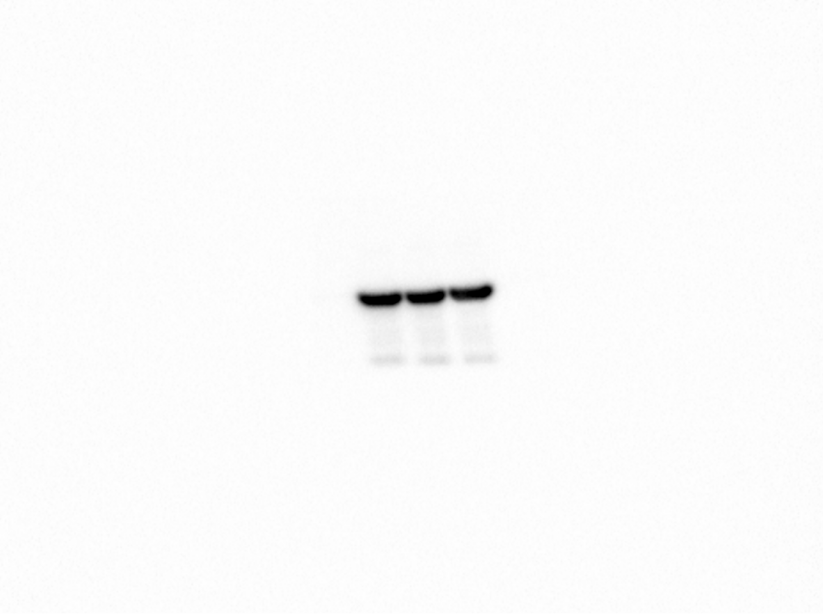


GAPDH-figure 3C-HOS


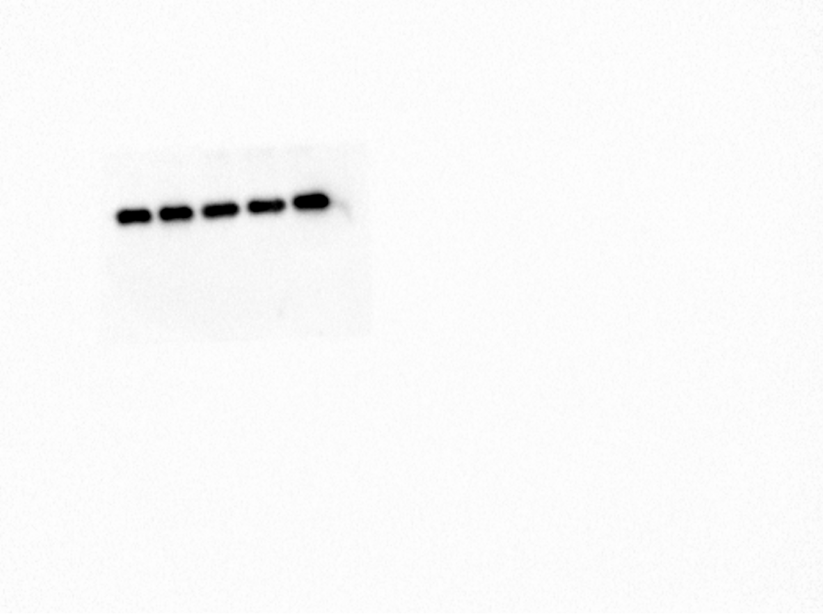


GAPDH-figure 3C-U2OS


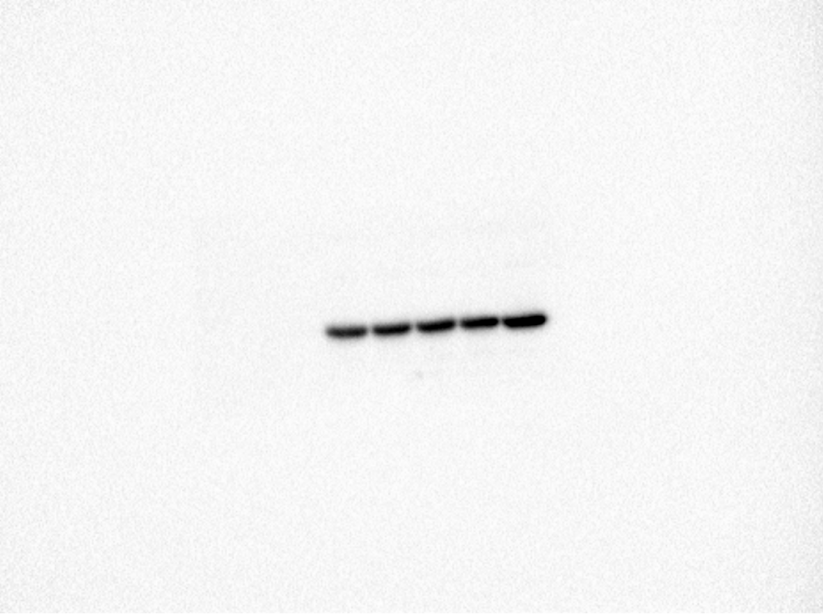


GAPDH-figure 3D-HOS


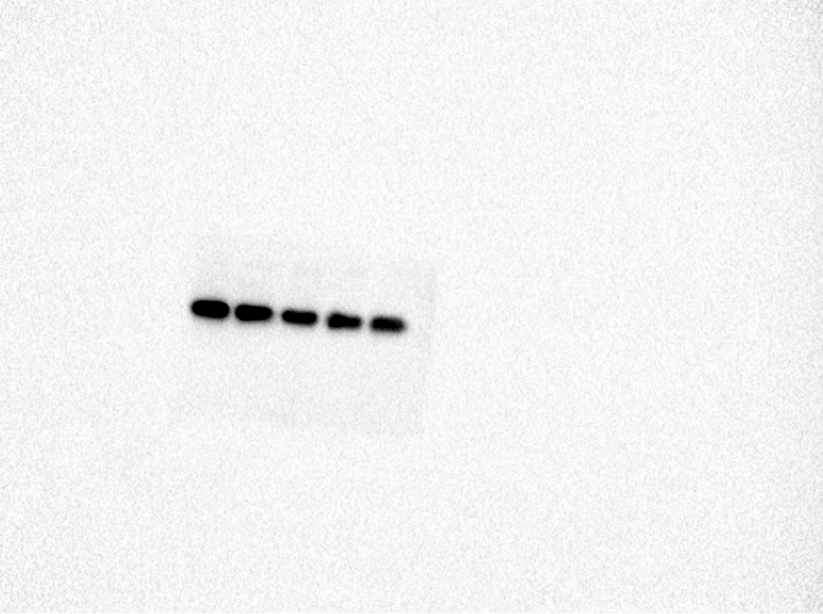


GAPDH-figure 3D-U2OS


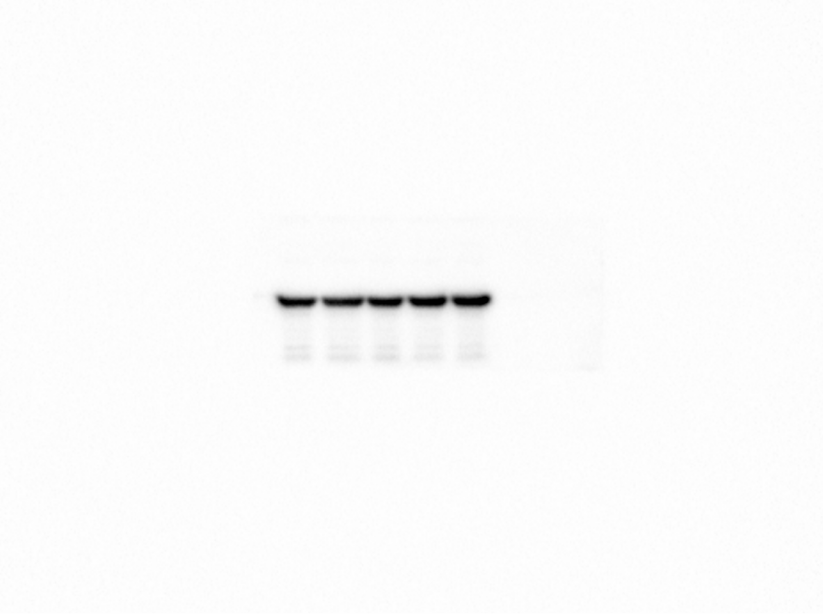


GAPDH-figure 4D-HOS


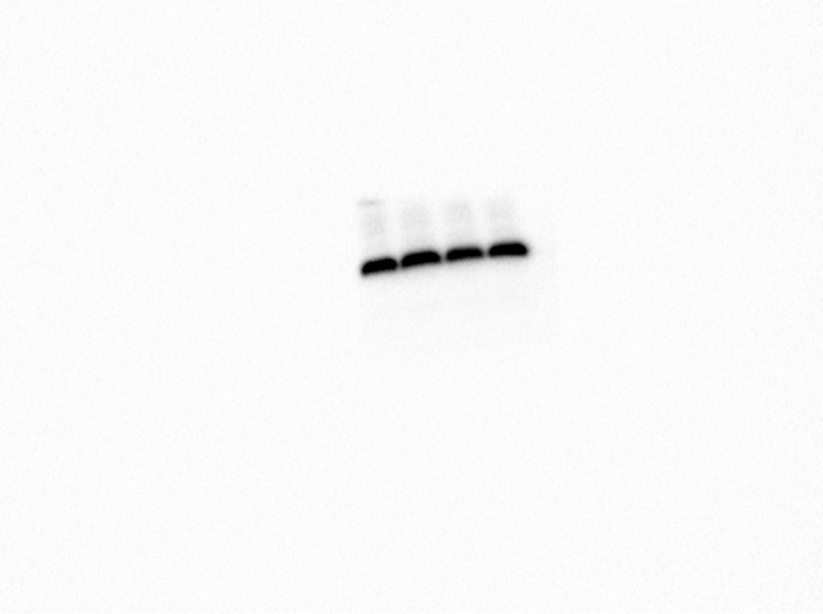


GAPDH-figure 4D-U2OS


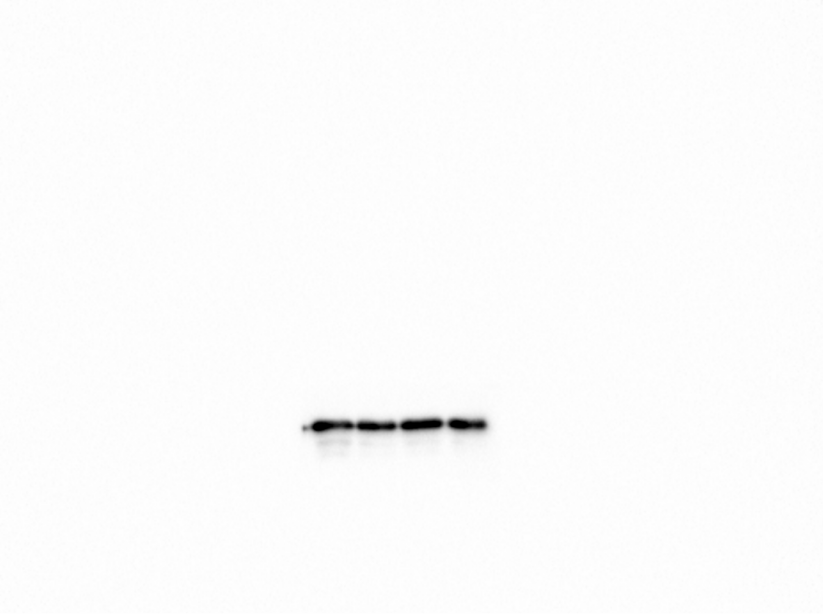


GAPDH-figure 5A-HOS


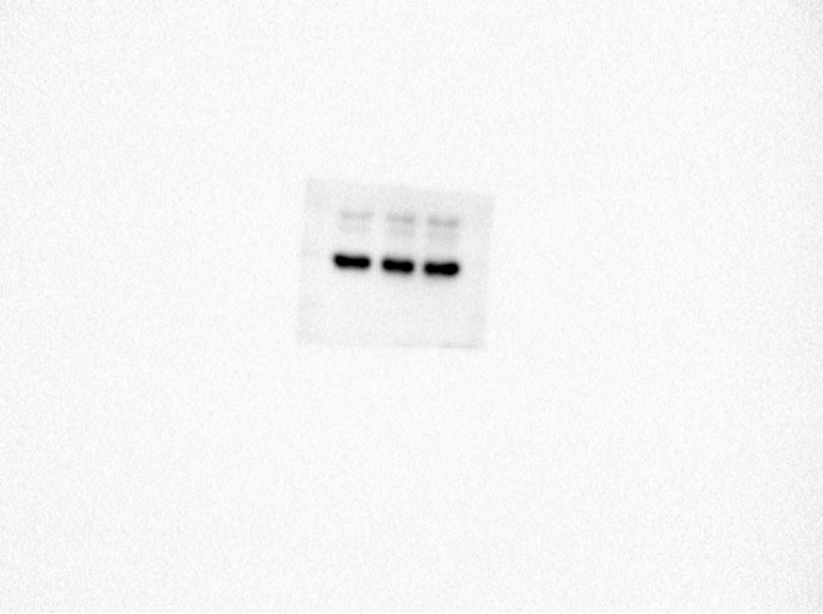


GAPDH-figure 5A-U2OS


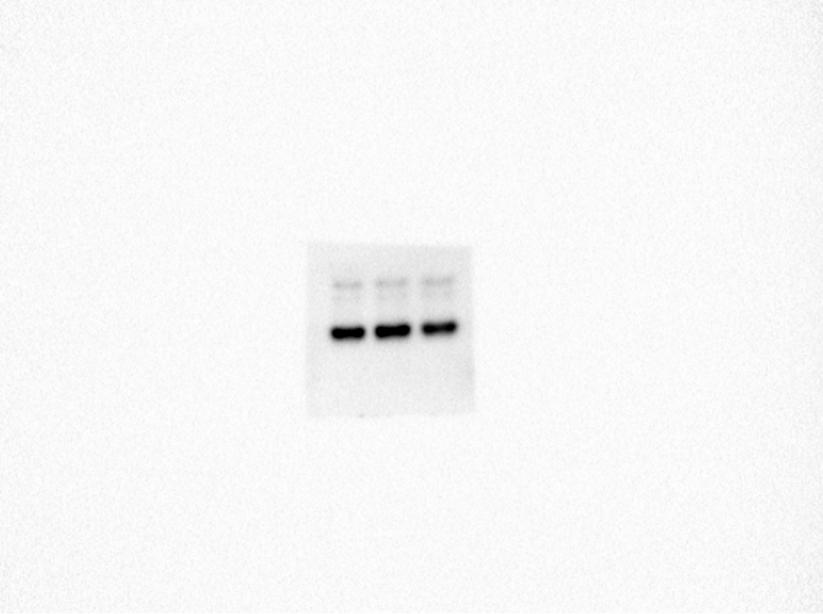


GAPDH-figure S3G-HOS


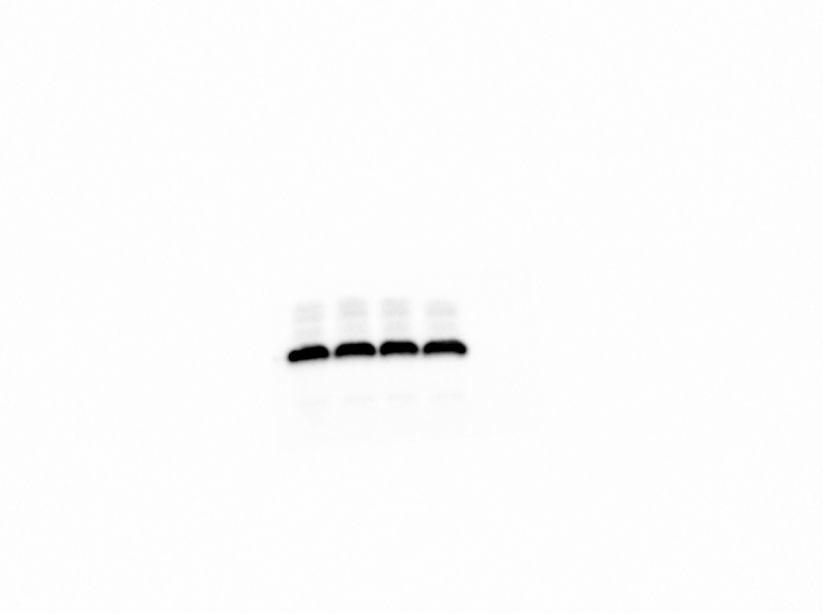


GAPDH-figure S3G-U2OS


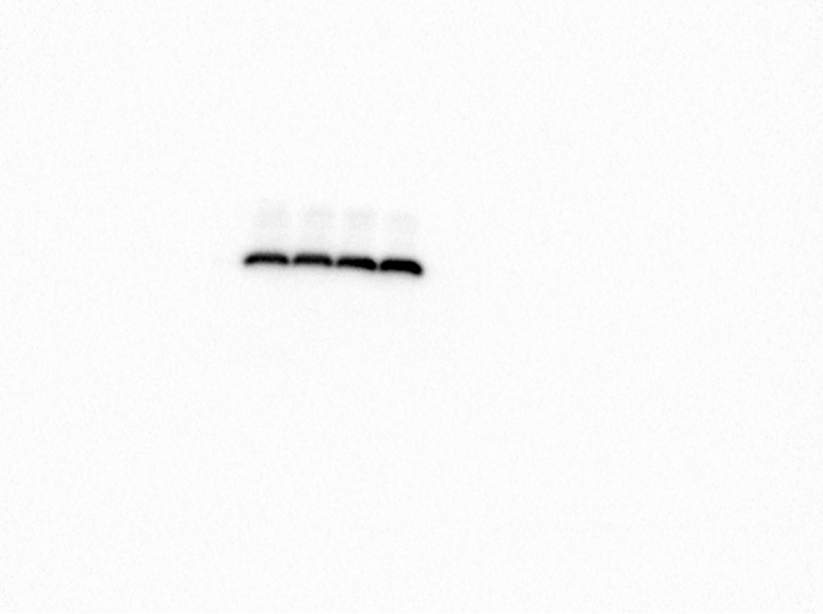


ITGB1-figure 2G


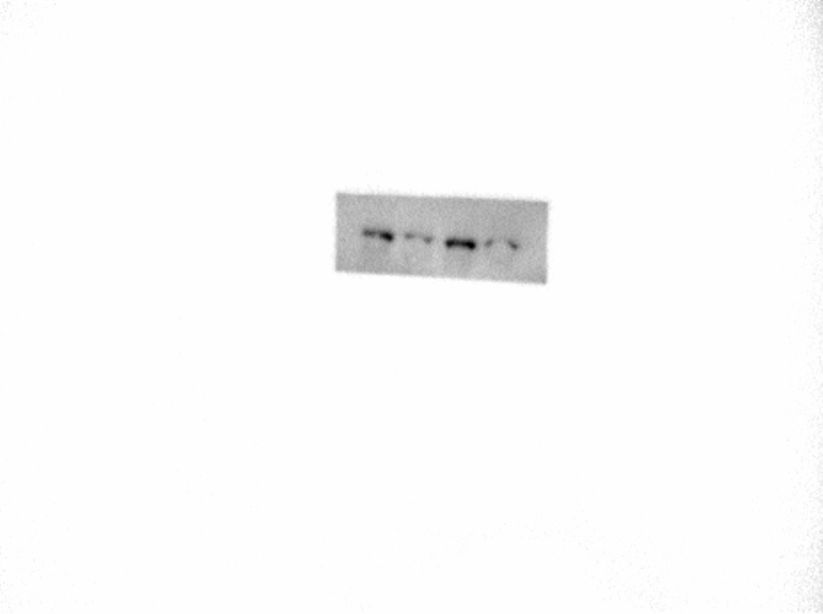


ITGB1-figure 2H


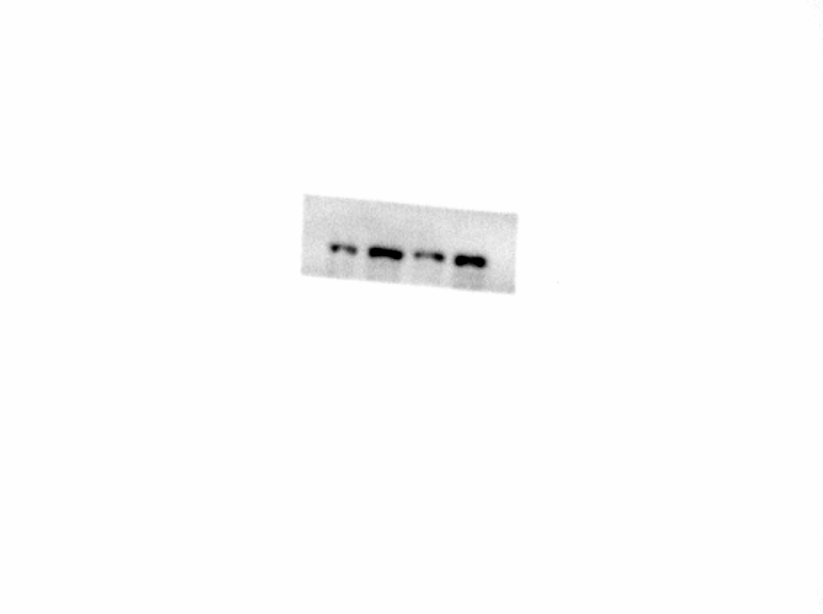


ITGB1-figure 2I


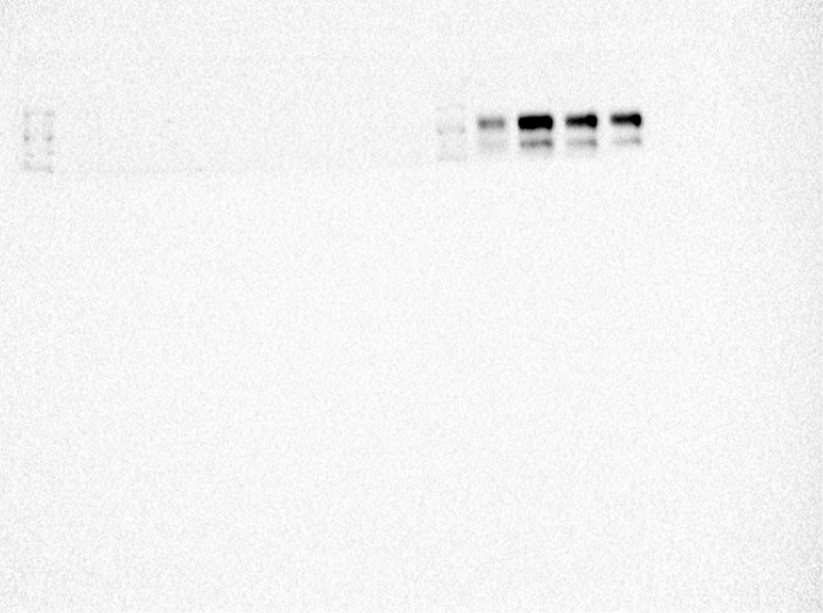


ITGB1-figure 2K


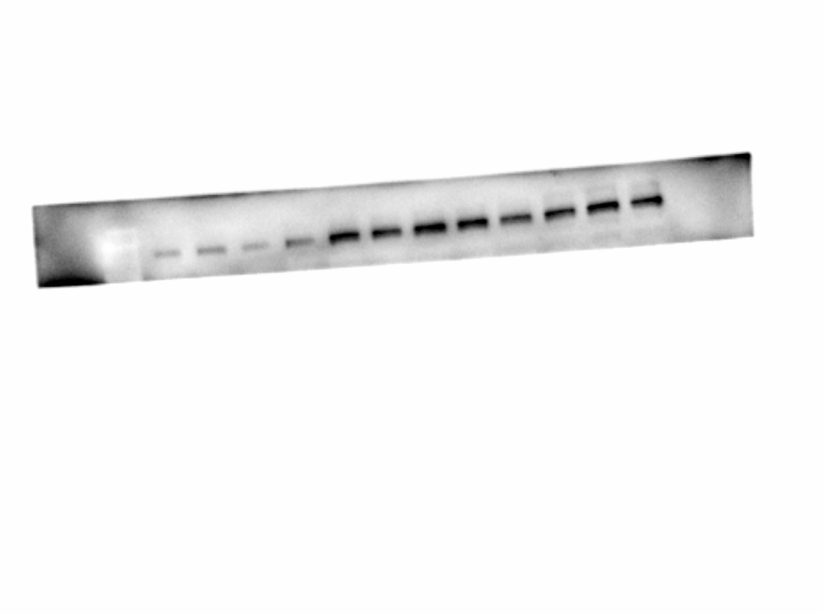


ITGB1-figure 3A-HOS


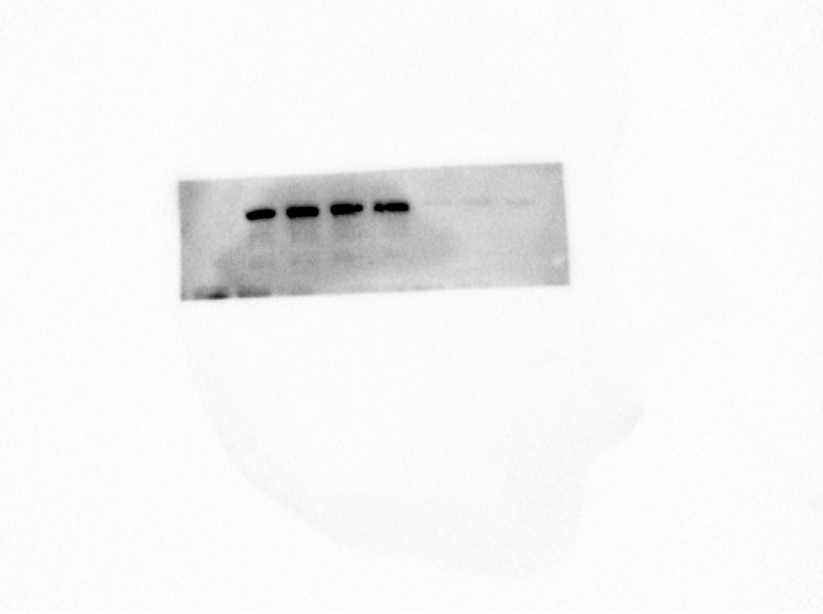


ITGB1-figure 3A-U2OS


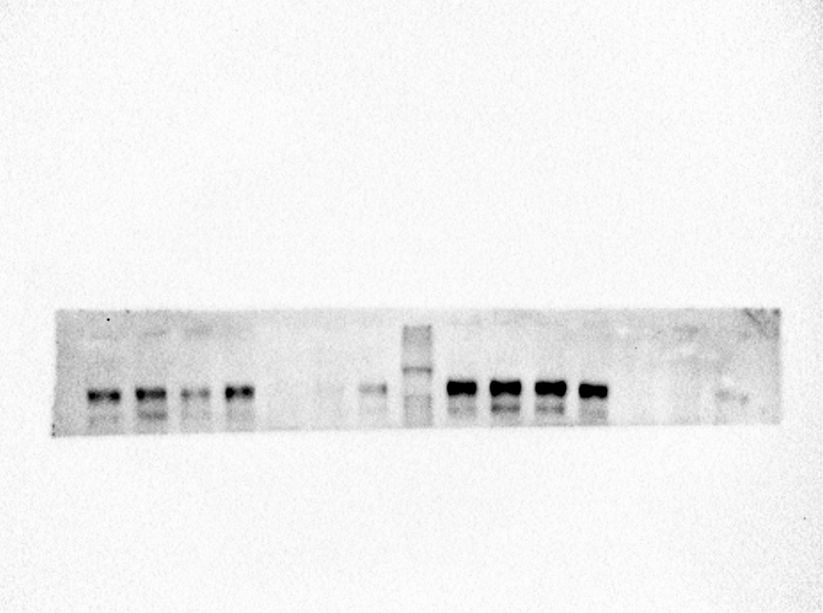


ITGB1-figure 3B-HOS


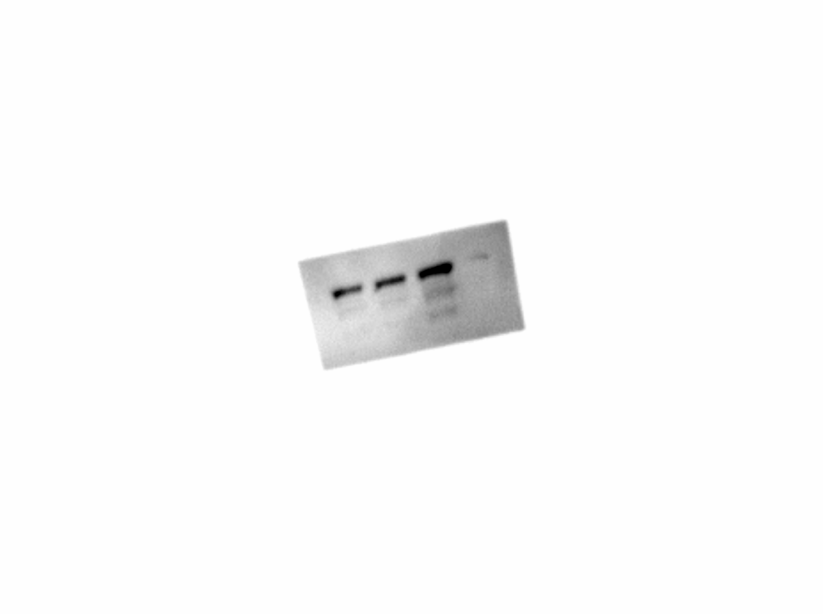


ITGB1-figure 3B-U2OS


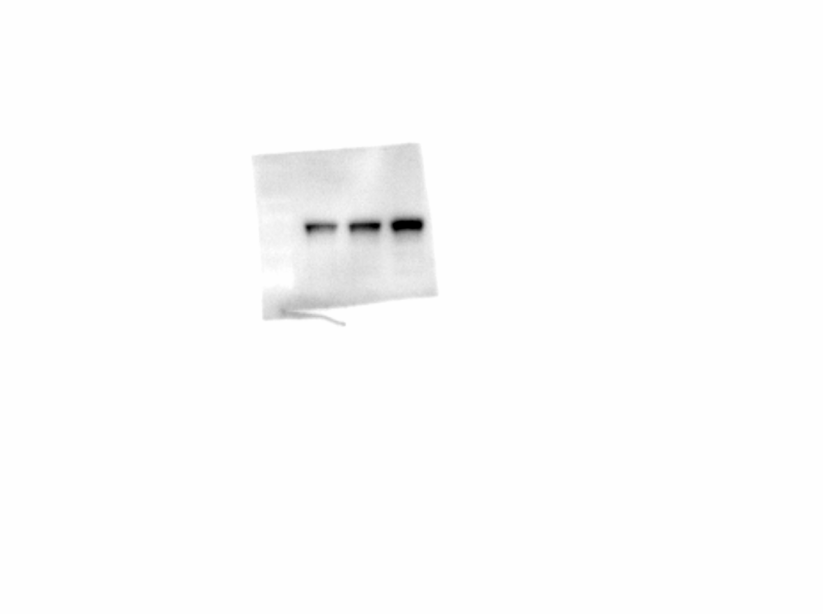


ITGB1-figure 3C-HOS


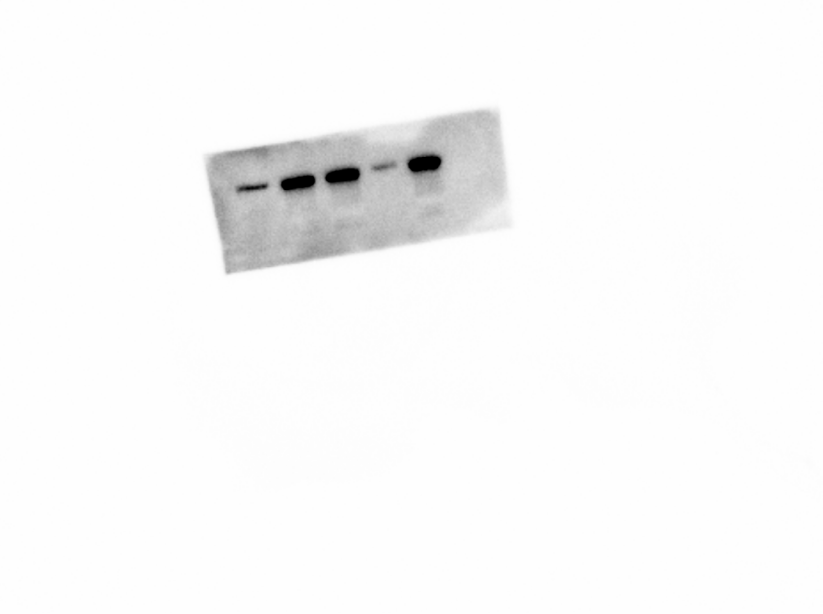


ITGB1-figure 3C-U2OS


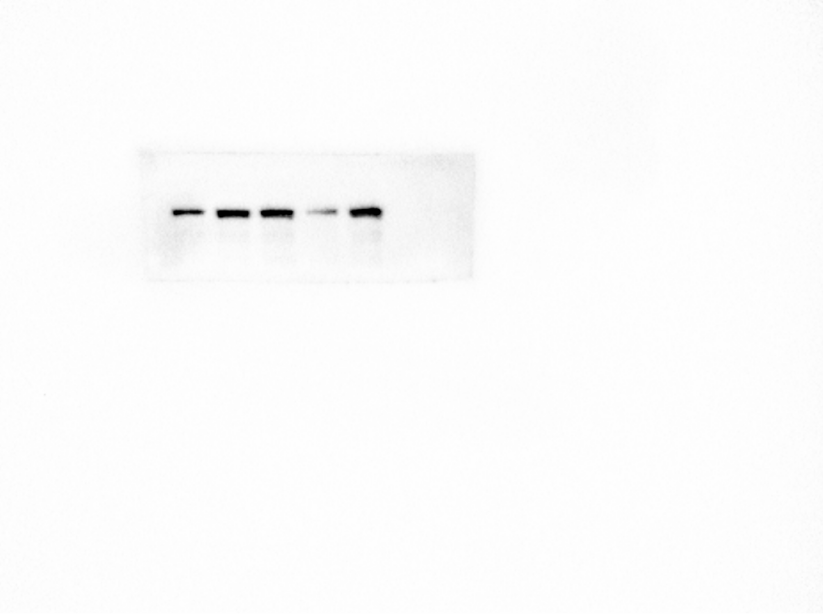


ITGB1-figure 3D-HOS


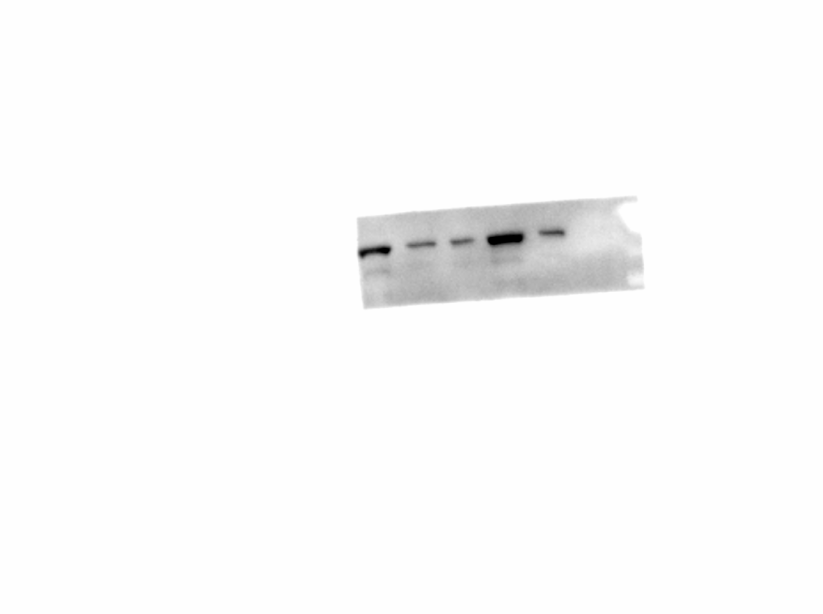


ITGB1-figure 3D-U2OS


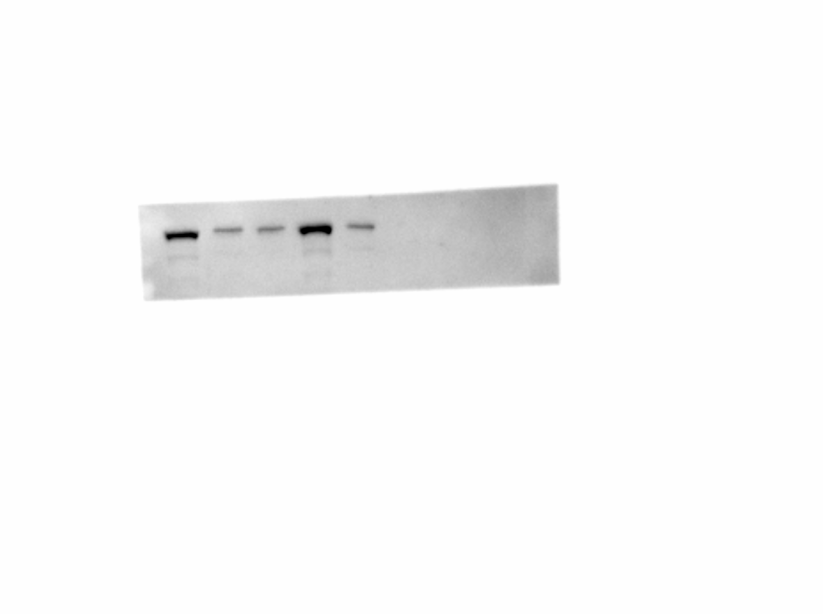


MMP2-figure 2G


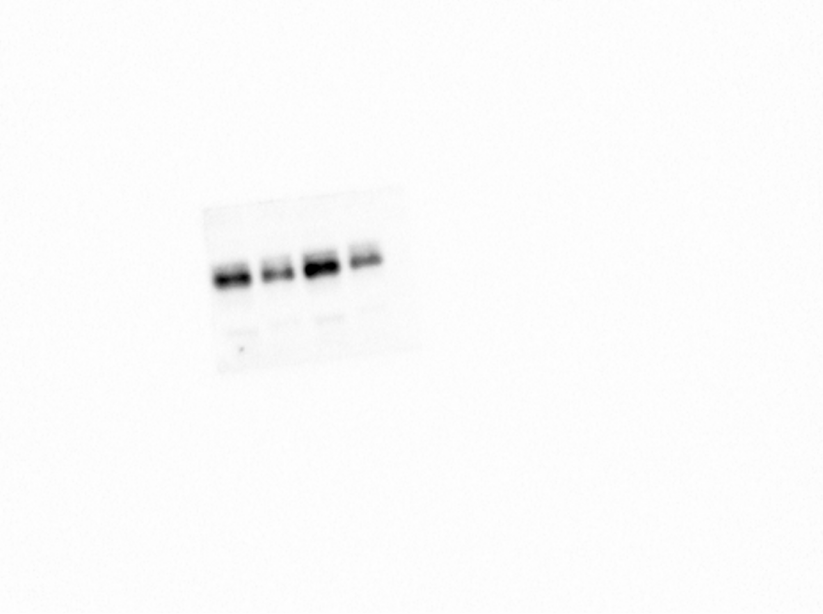


MMP2-figure 2H


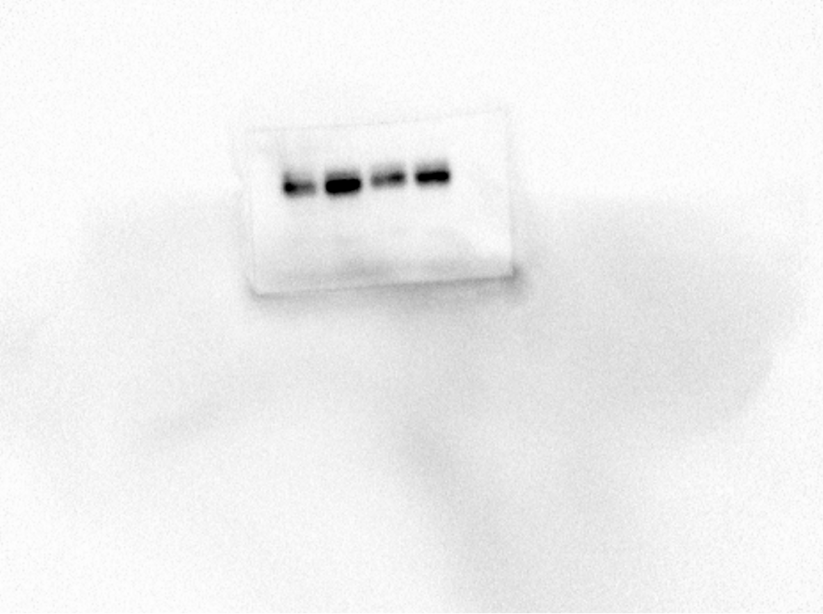


MMP2-figure 2I


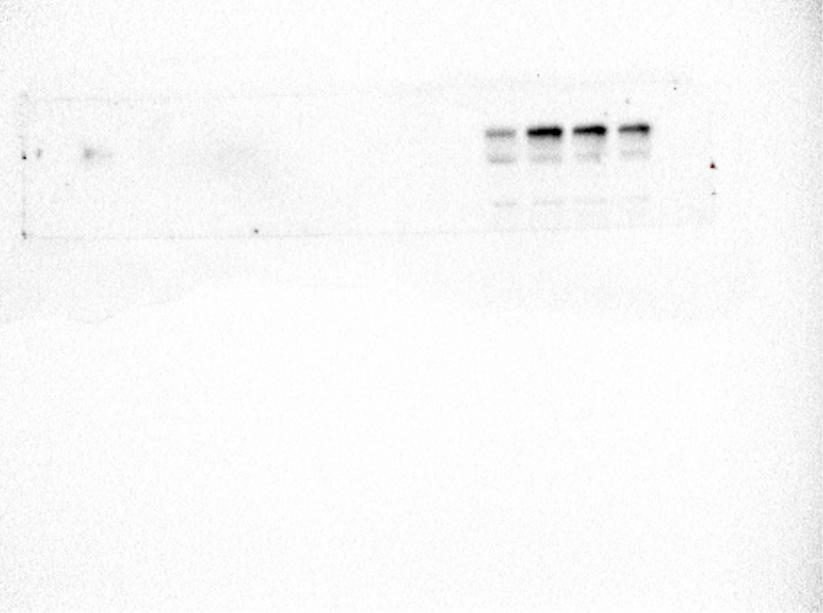


MMP2-figure 2K


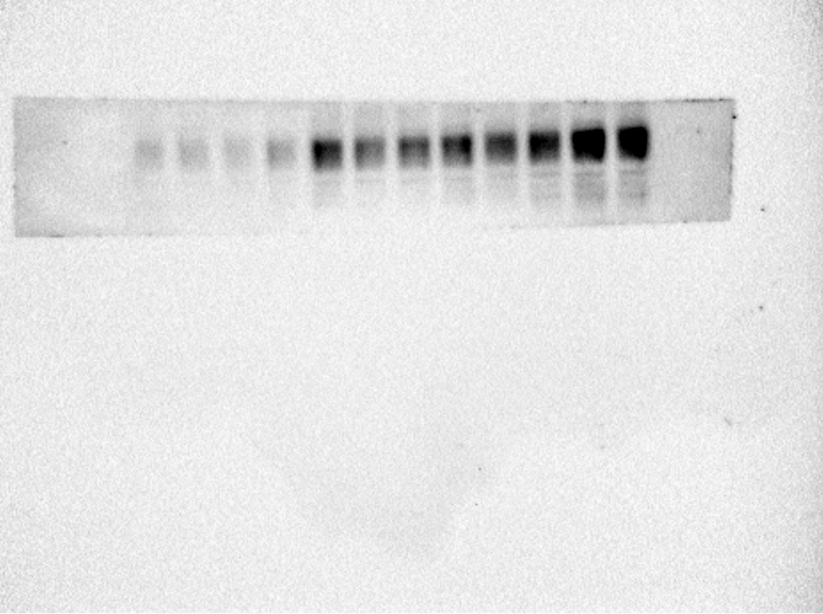


MMP2-figure 3A-HOS


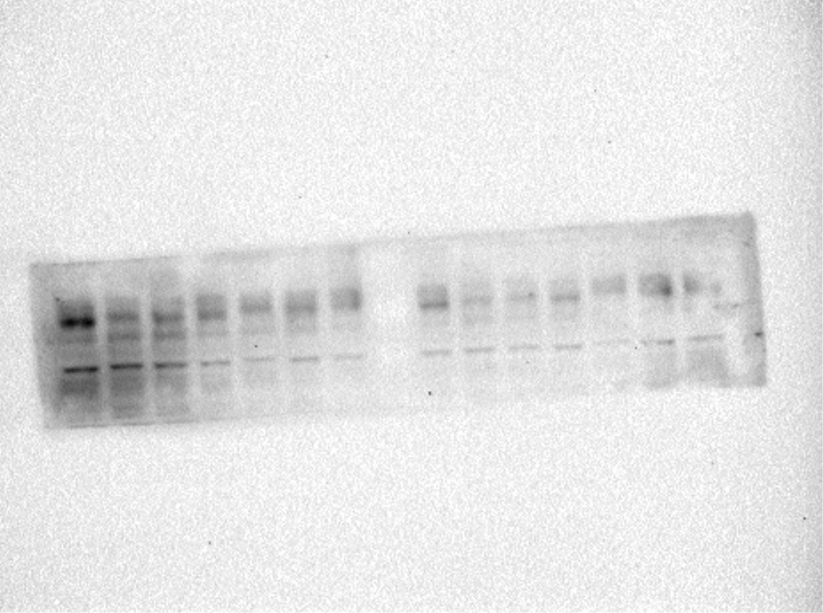


MMP2-figure 3A-U2OS


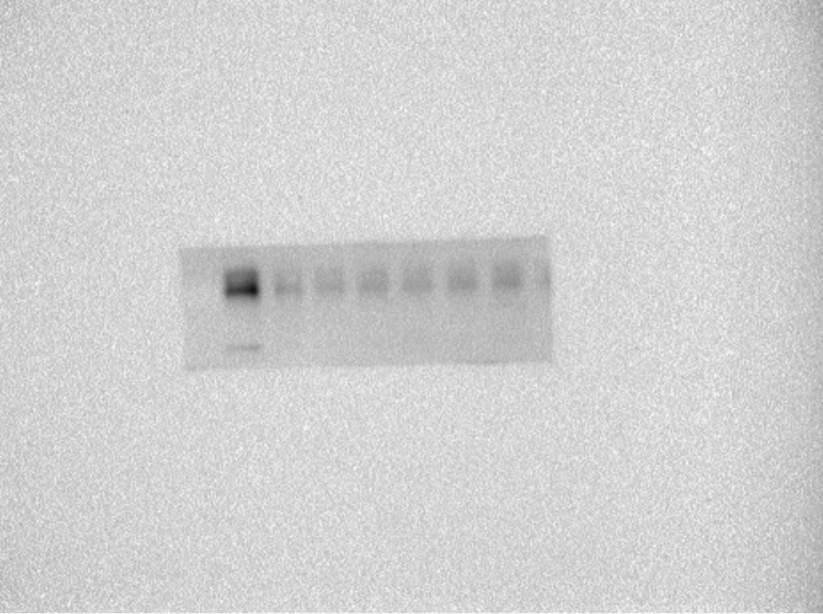


MMP2-figure 3B-HOS


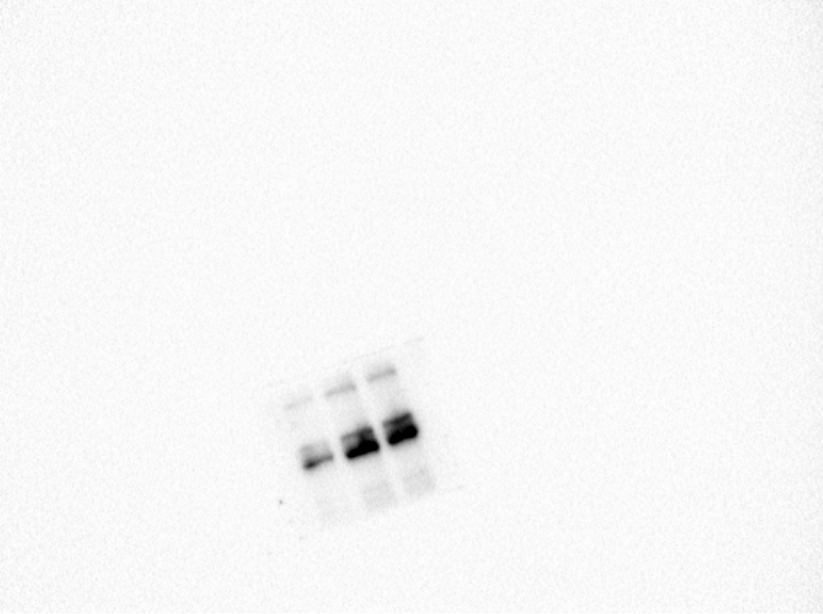


MMP2-figure 3B-U2OS


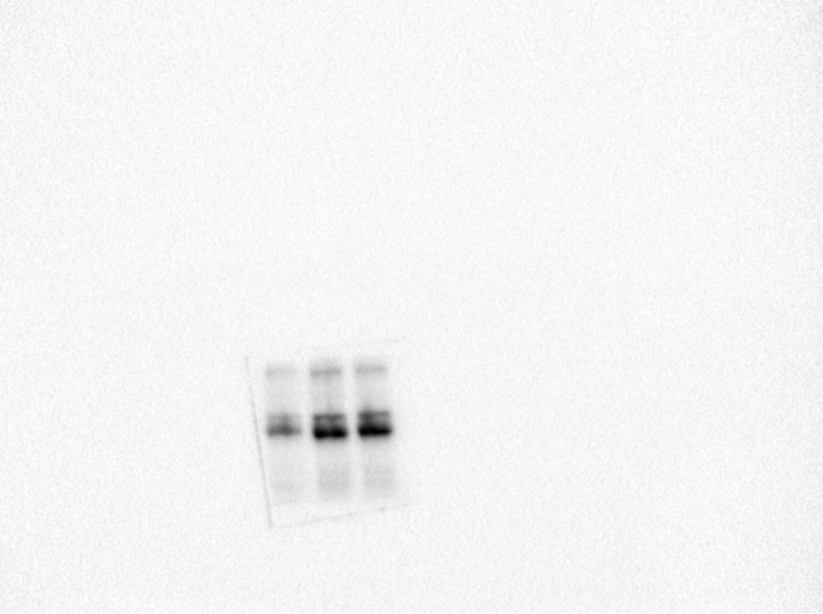


MMP2-figure 3C-HOS


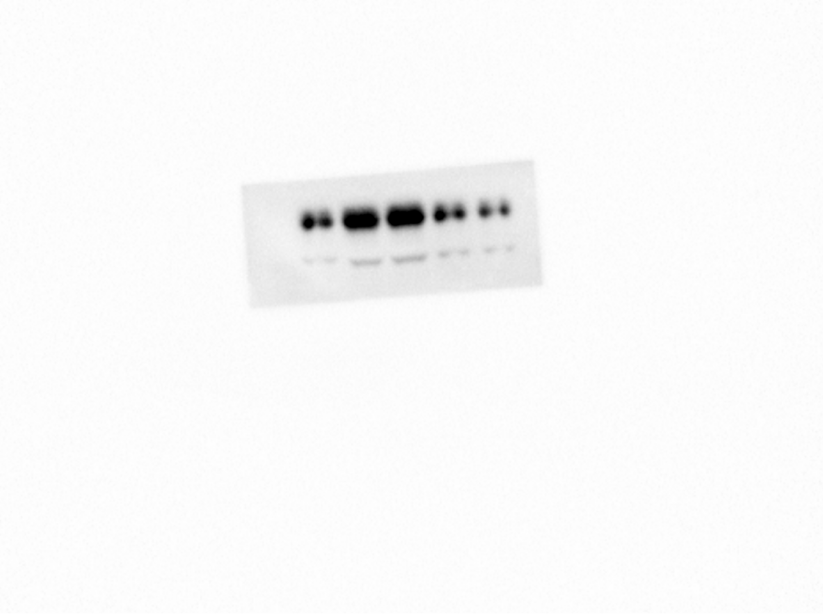


MMP2-figure 3C-U2OS


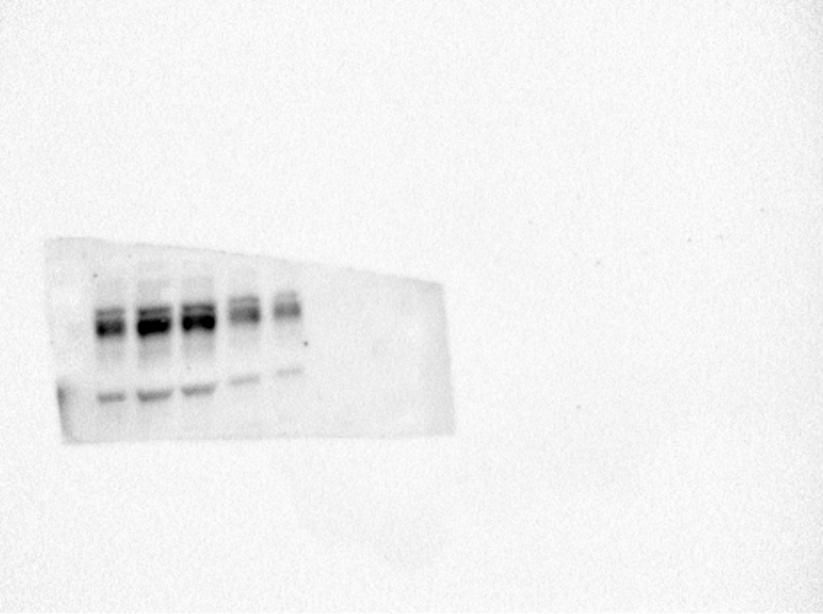


MMP2-figure 3D-HOS


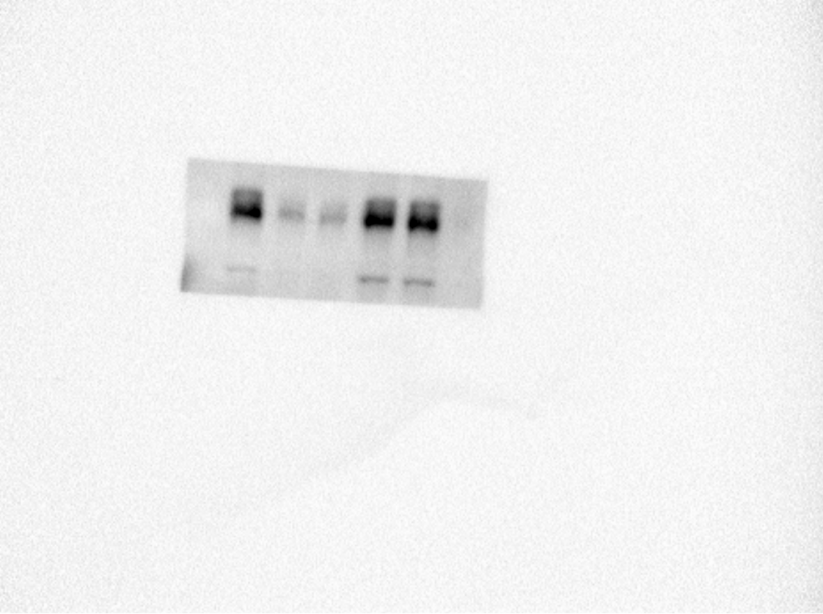


MMP2-figure 3D-U2OS


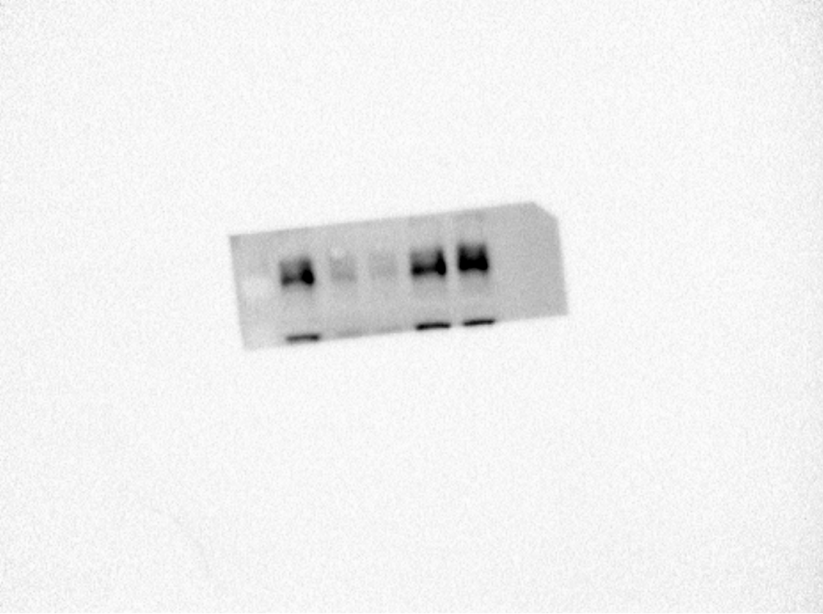


p-AKT-figure 2D-HOS


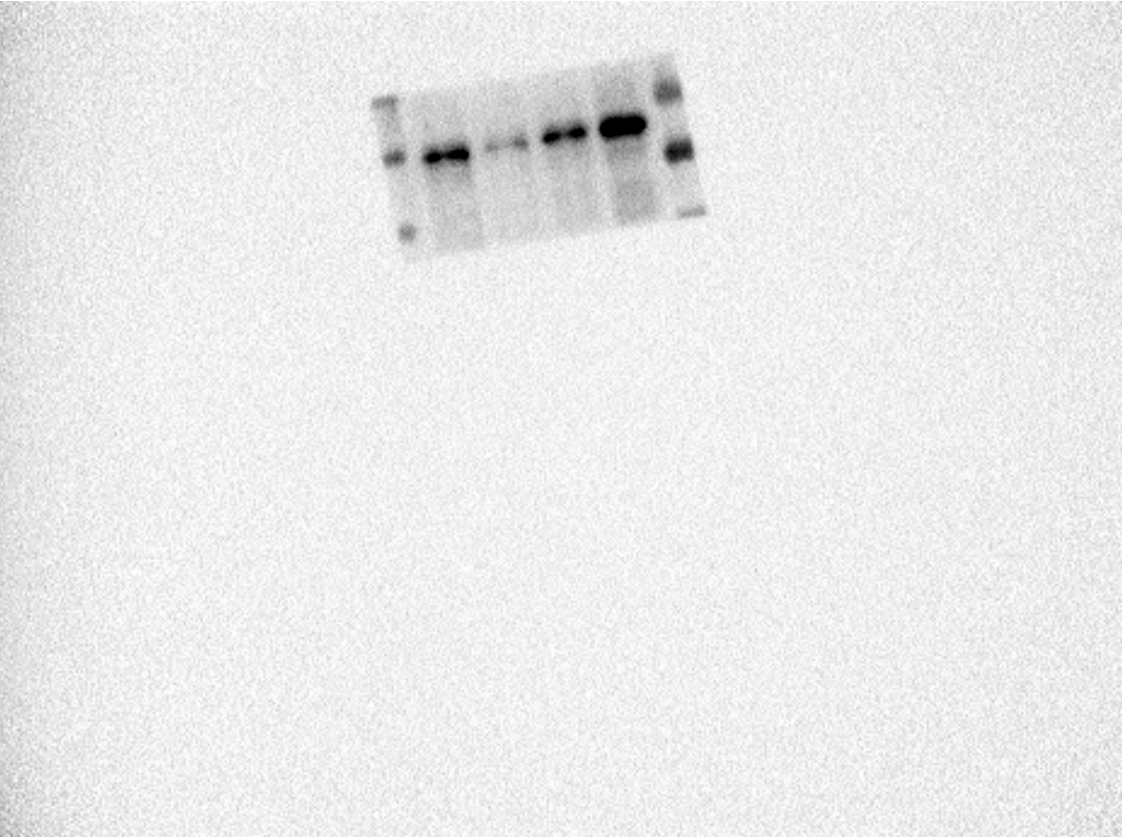


p-AKT-figure 2D-U2OS


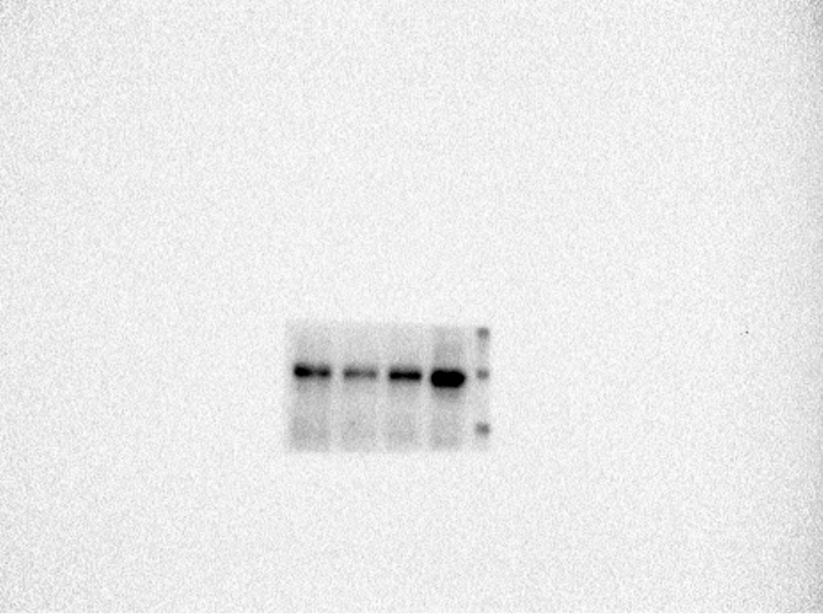


p-AKT-figure 4D-HOS


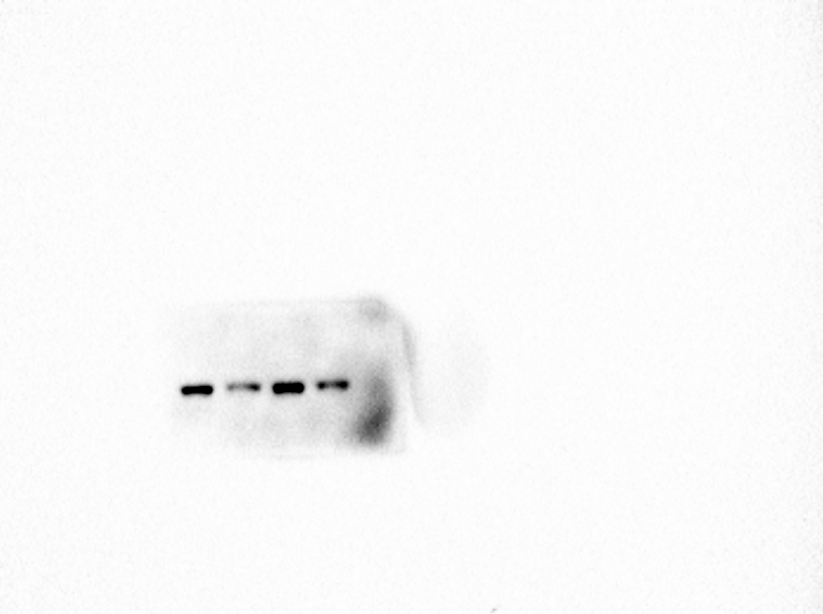


p-AKT-figure 4D-U2OS


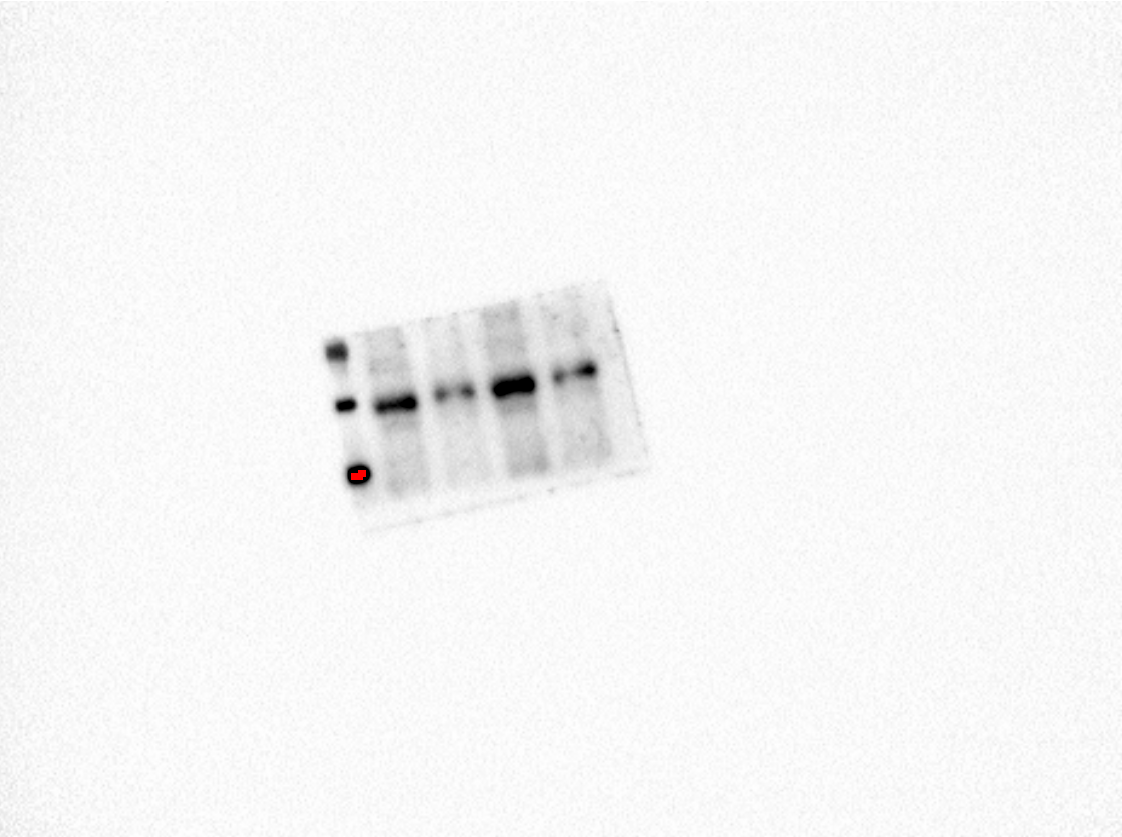


p-AKT-figure 5A-HOS


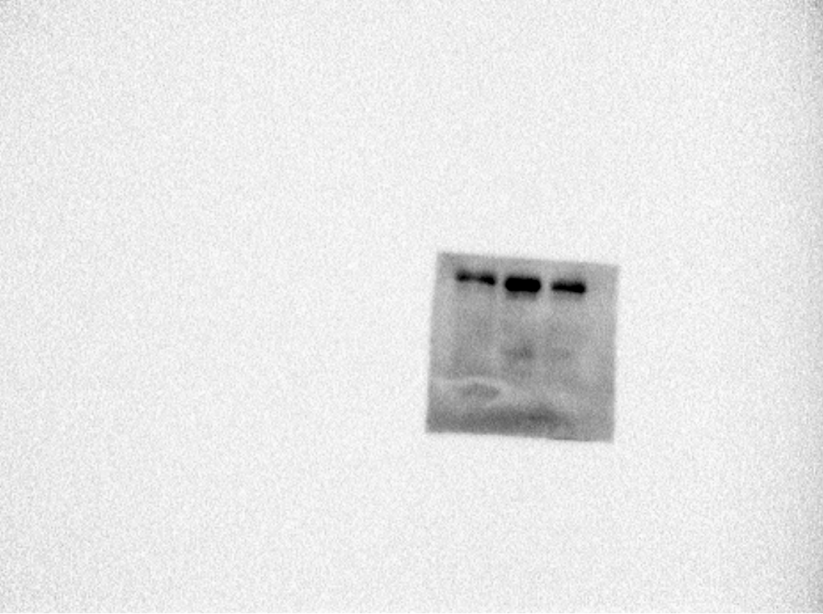


p-AKT-figure 5A-U2OS


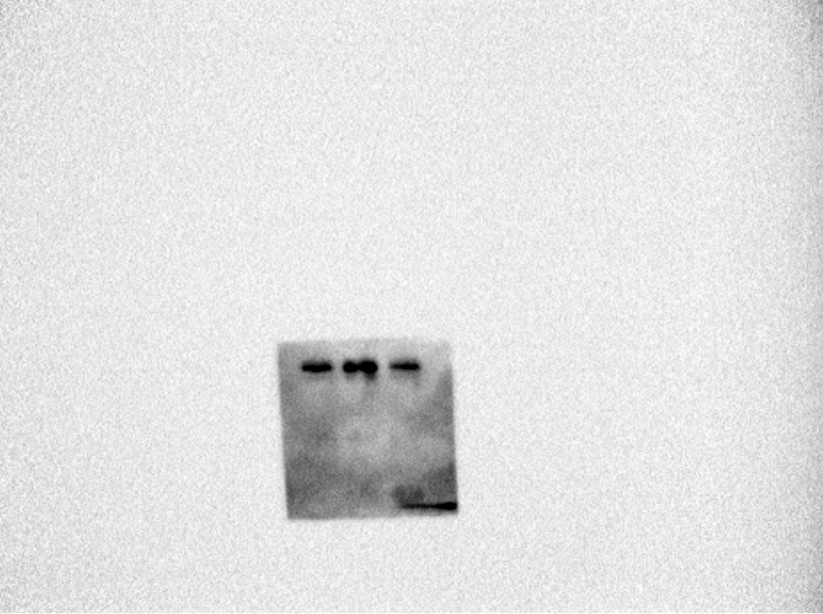


p-AKT-figure S3G-HOS


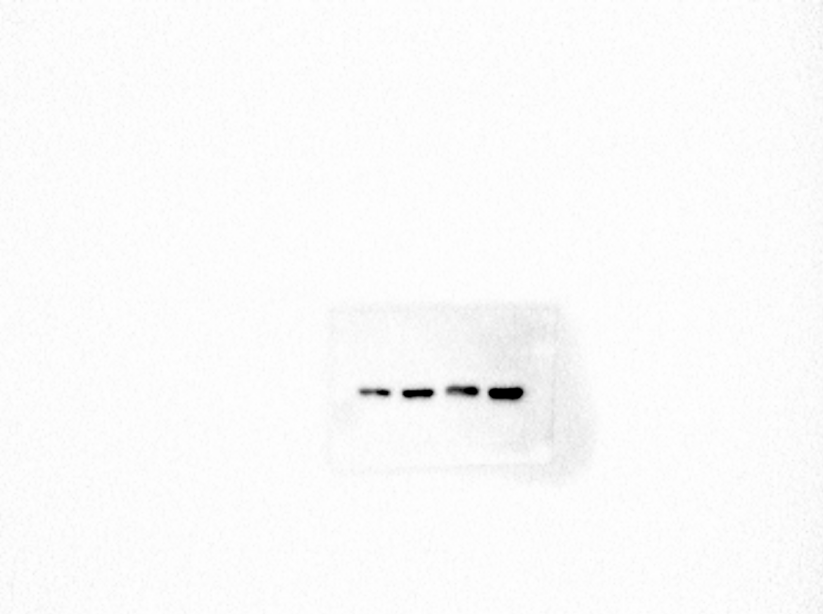


p-AKT-figure S3G-U2OS


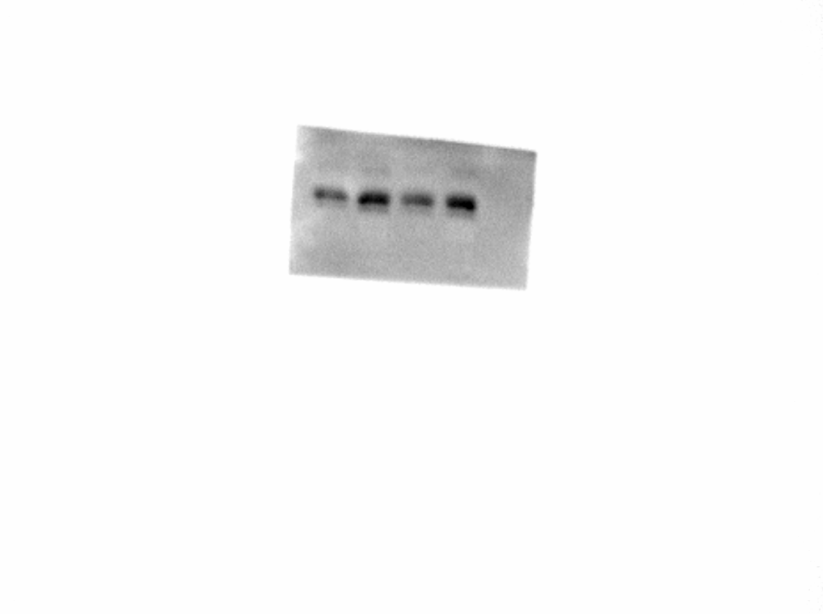


PI3K-figure 2D-HOS


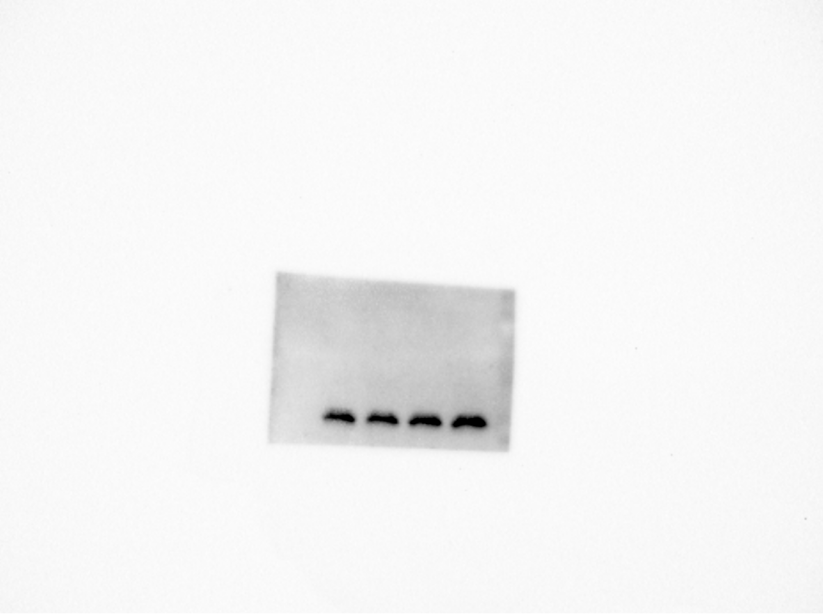


PI3K-figure 2D-U2OS


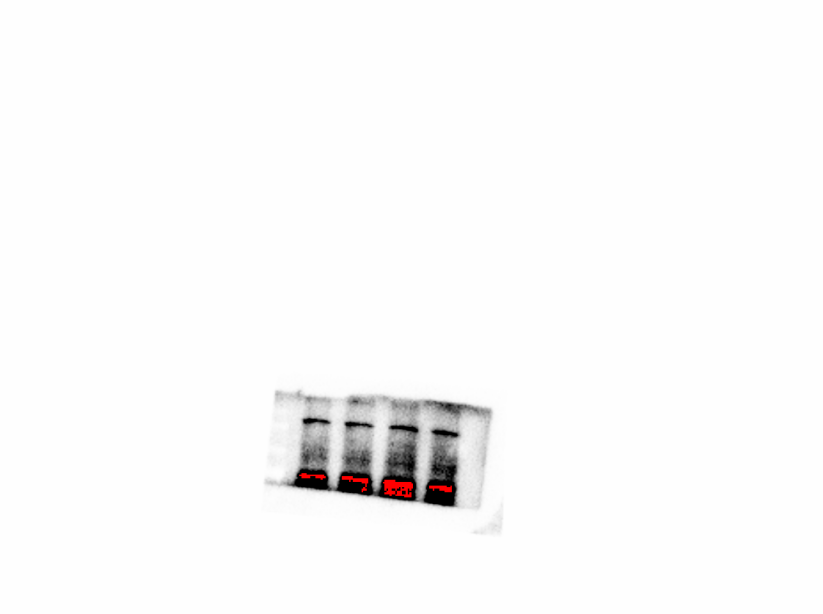


PI3K-figure 4D-HOS


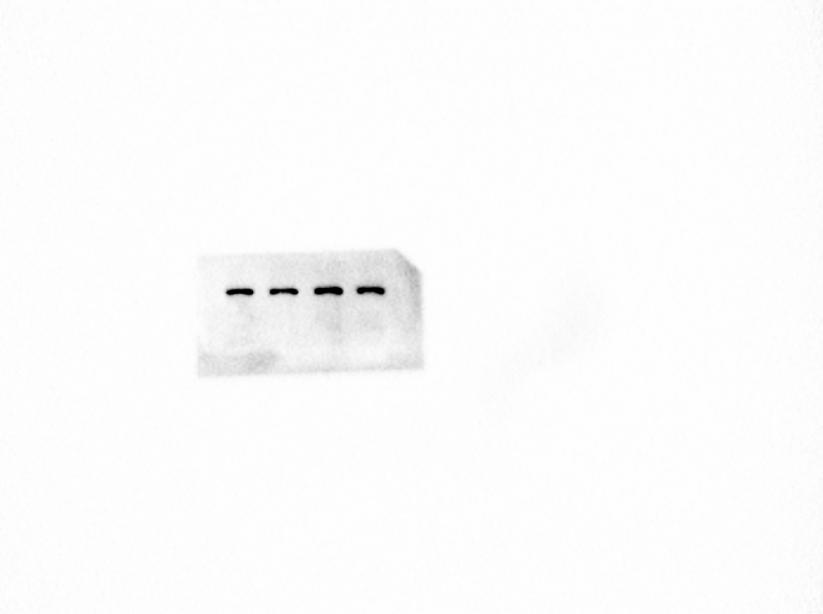


PI3K-figure 4D-U2OS


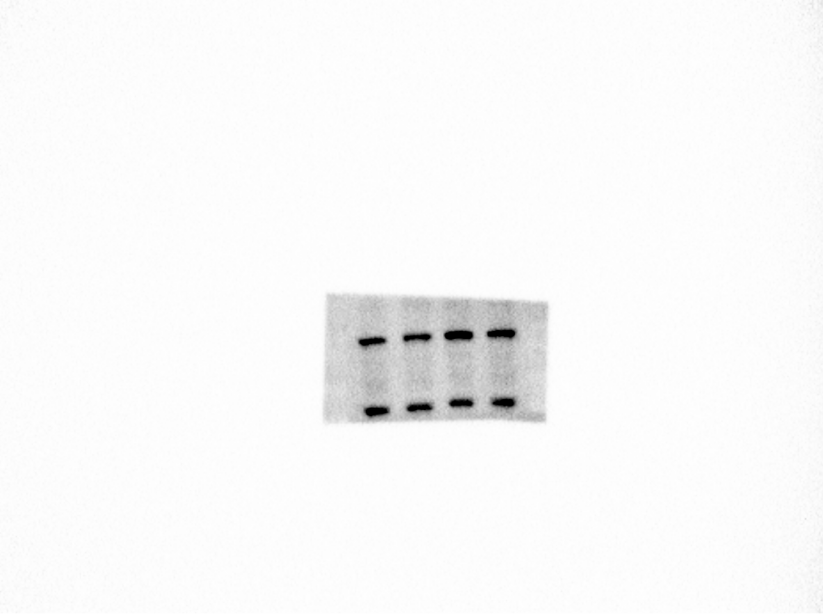


PI3K-figure 5A-HOS


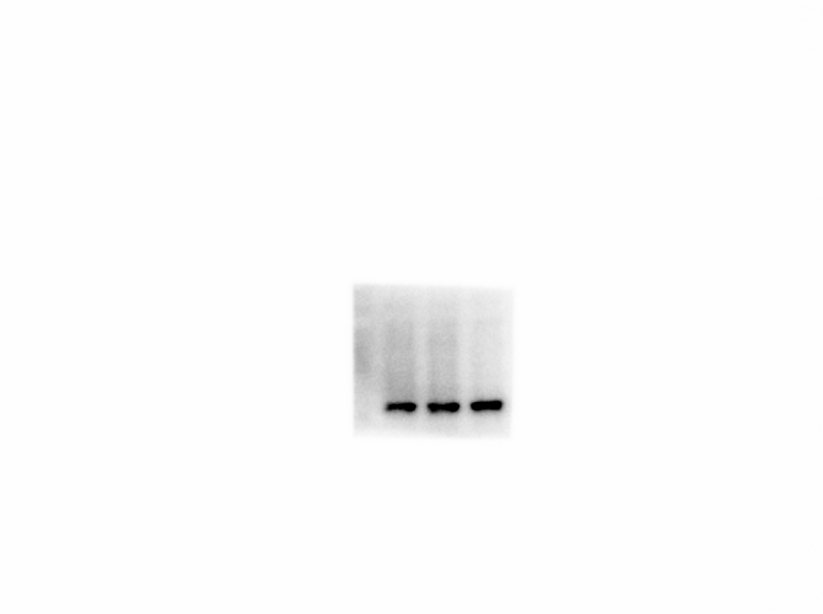


PI3K-figure 5A-U2OS


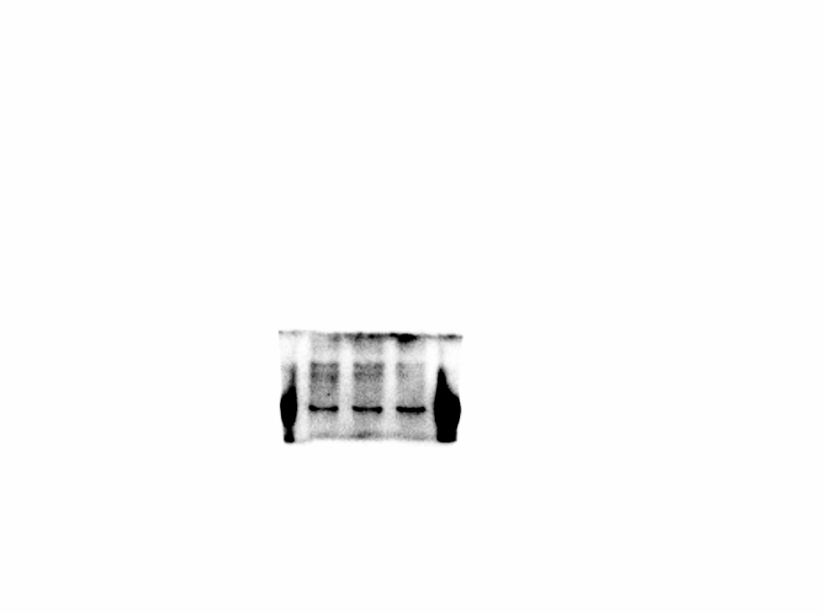


PI3K-figure S3G-HOS


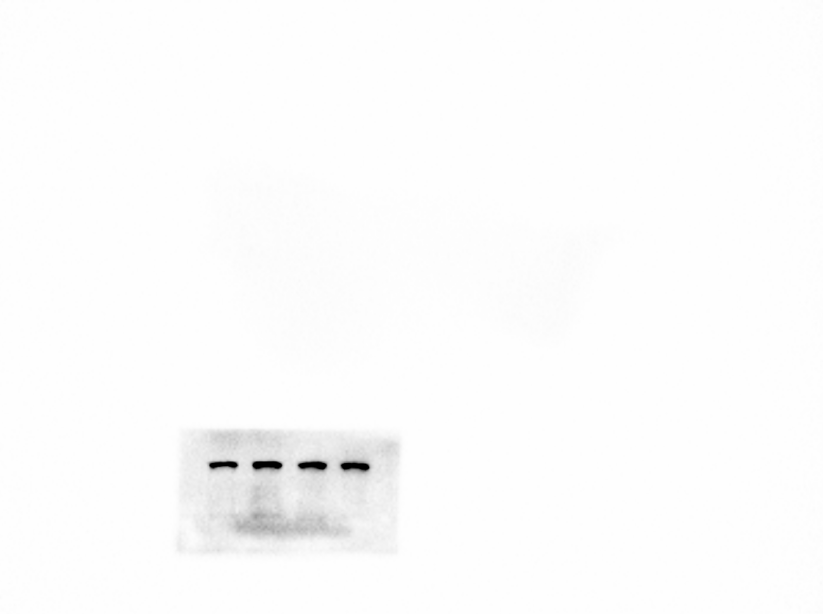


PI3K-figure S3G-U2OS


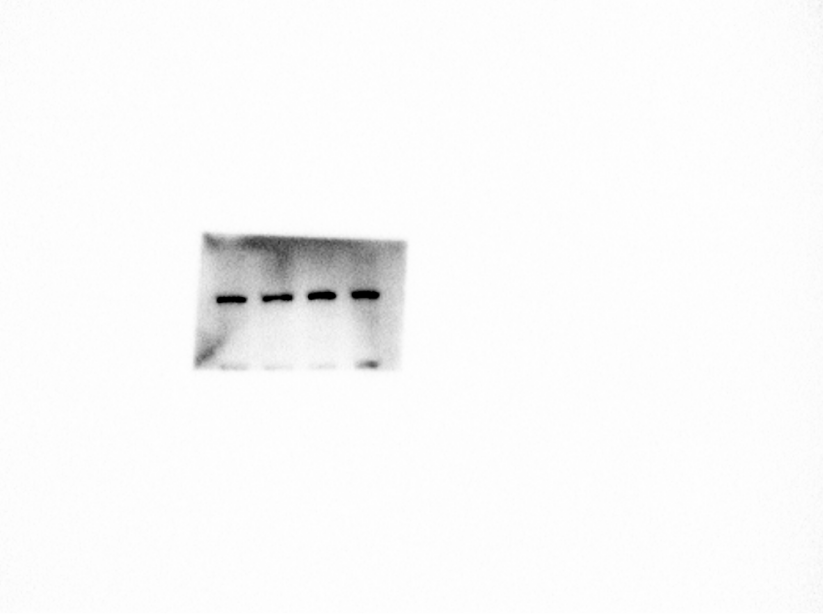


p-PI3K-figure 2D-HOS


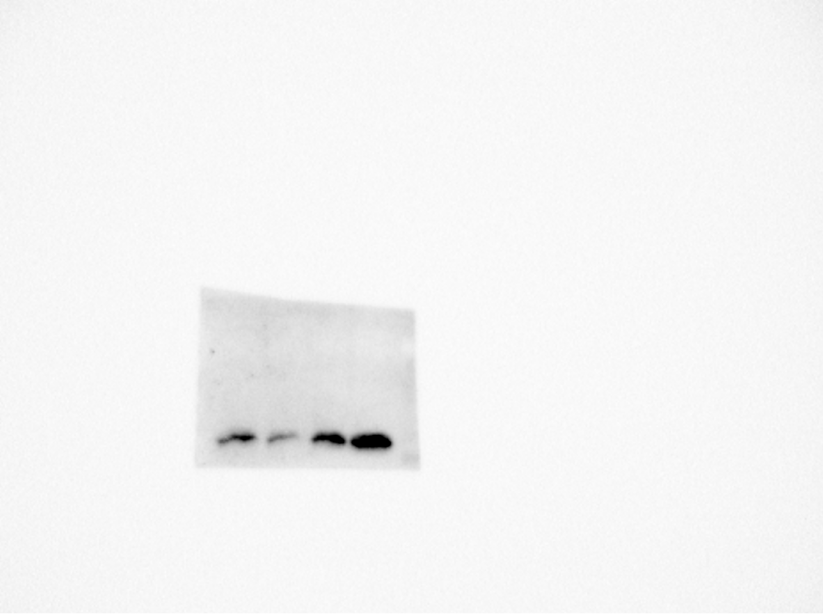


p-PI3K-figure 2D-U2OS


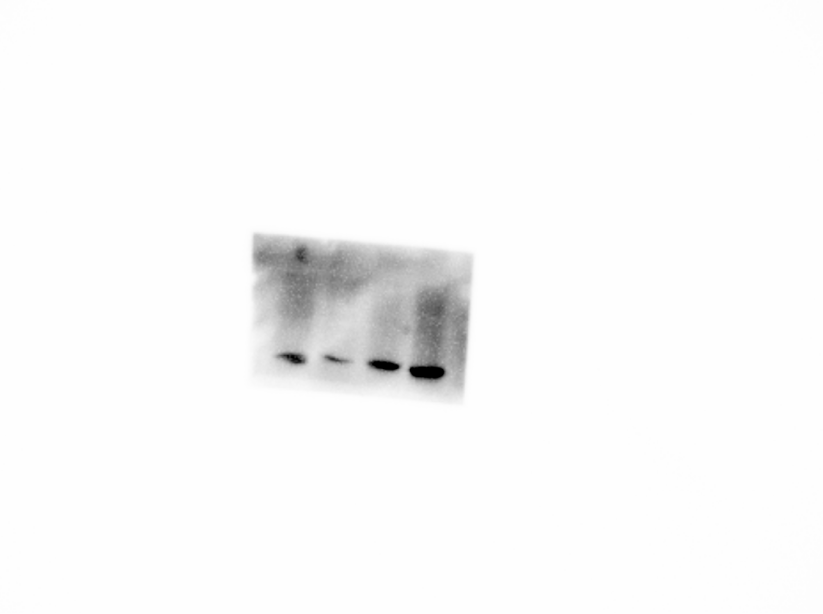


p-PI3K-figure 4D-HOS


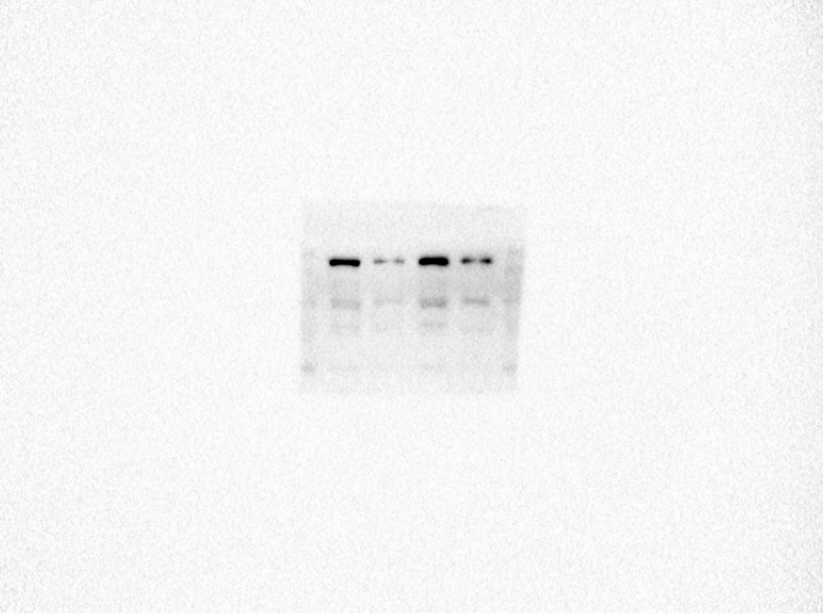


p-PI3K-figure 4D-U2OS


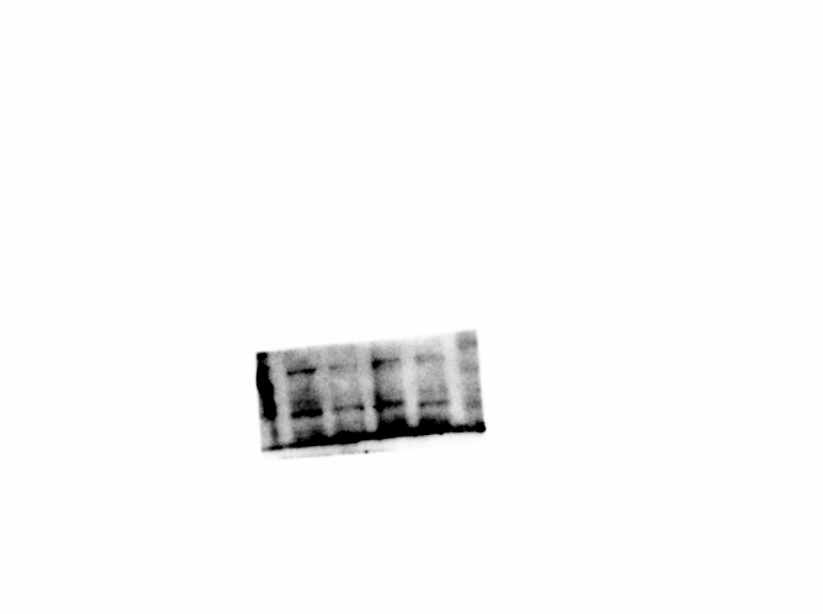


p-PI3K-figure 5A-HOS


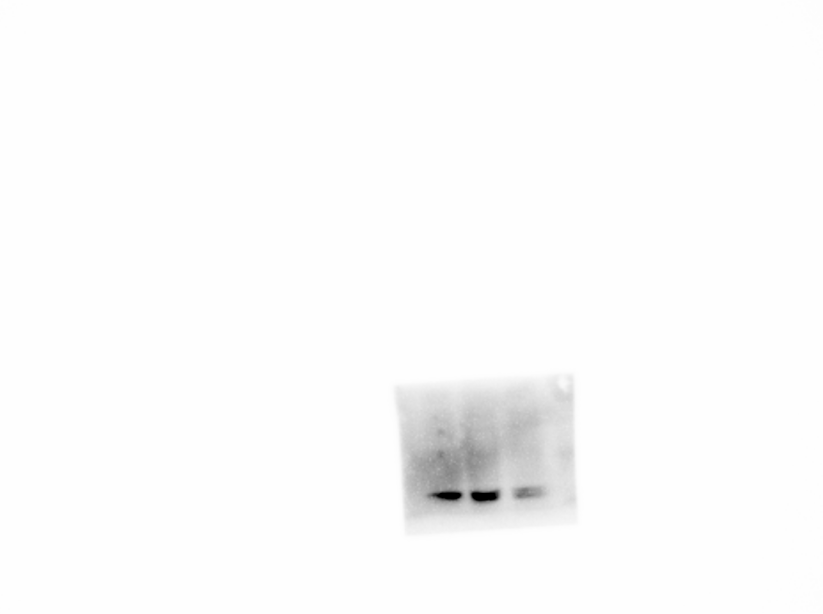


p-PI3K-figure 5A-U2OS


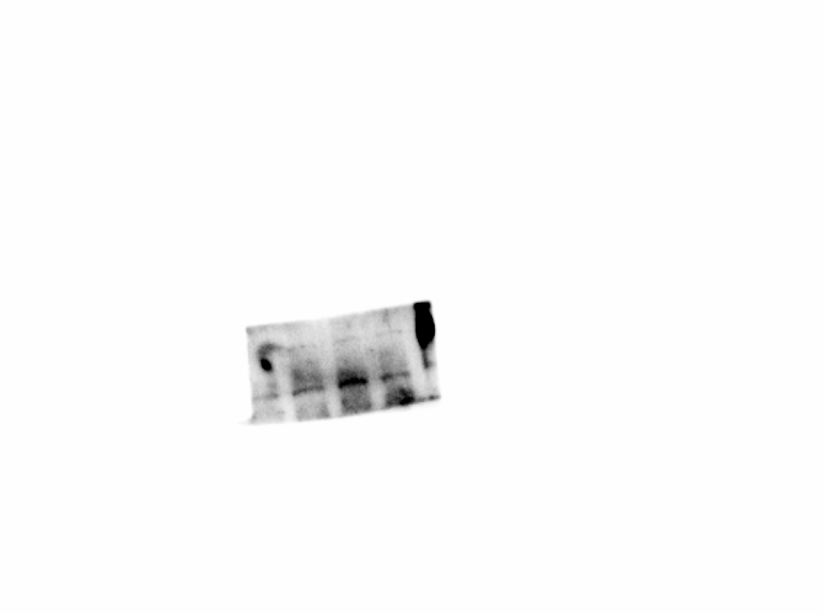


p-PI3K-figure S3G-HOS


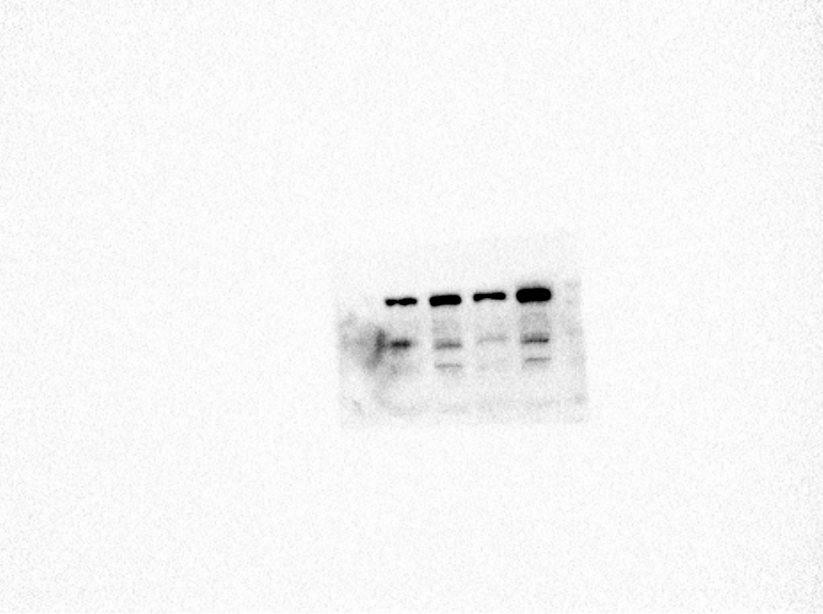


p-PI3K-figure S3G-U2OS


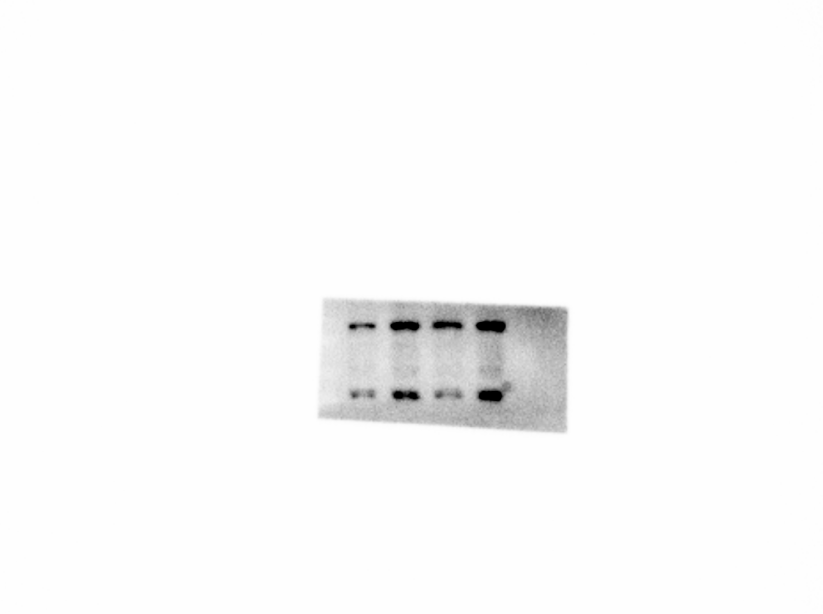

Supplement: Supplementary file 7 — Original western blots [file 41420_2024_1946_MOESM7_ESM.docx]
